# Straightforward Synthetic Approach to Aminoalcohols with 9-oxabicyclo[3.3.1]nonane or Cyclooctane Core via Nucleophilic Ring-Opening of Spirocyclic Bis(oxiranes)

**DOI:** 10.3390/molecules31020252

**Published:** 2026-01-12

**Authors:** Olga V. Ryzhikova, Daiana V. Savchenkova, Sergey V. Kositov, Yuri K. Grishin, Olga A. Maloshitskaya, Kseniya N. Sedenkova, Elena B. Averina

**Affiliations:** Department of Chemistry, Lomonosov Moscow State University, Leninskie Gory 1-3, 119991 Moscow, Russia; olga.ryzhikova@chemistry.msu.ru (O.V.R.); daiana.savchenkova@chemistry.msu.ru (D.V.S.); sergei.kositov@chemistry.msu.ru (S.V.K.); grishin@nmr.chem.msu.ru (Y.K.G.); maloshitskaya@org.chem.msu.ru (O.A.M.); sedenkova@med.chem.msu.ru (K.N.S.)

**Keywords:** oxiranes, amines, 9-oxabicyclo[3.3.1]nonanes, cyclooctanes, aminodiols, ring-opening, domino reaction, intramolecular cyclization

## Abstract

Nucleophilic ring-opening of bis(oxiranes), containing several reactive centers, can be used to elaborate straightforward atom-economy and stereoselective approaches to polyfunctionalized compounds. In the present work, ring-opening of cis- and trans-diastereomers of a spirocyclic bis(oxirane), containing a cyclooctane core (namely, 1,8-dioxadispiro[2.3.2.3]dodecane), upon treatment with various amines, was studied. Trans-isomer afforded aminoalcohols with 9-oxabicyclo[3.3.1]nonane moiety, formed via domino-process, including opening of an oxirane ring followed by intramolecular cyclization. Ring-opening of cis-isomer gave aminosubstituted cis-cyclooctane-1,5-diols, derived from independent reaction of two oxirane moieties. Activation of oxirane rings by the addition of LiClO_4_, acting as a Lewis acid, allowed the involvement of a number of primary and secondary aliphatic amines as well as aniline derivatives in the reaction. Scope and limitations of the reaction were studied and a series of aminoalcohols with a 9-oxabicyclo[3.3.1]nonane core and symmetric diaminodiols with a cyclooctane core were obtained.

## 1. Introduction

Oxiranes represent versatile intermediates, finding application in the synthesis of medicinal drugs, natural compounds, polymers, and other products with practicable properties [1,2,3,4,5]. Synthetically useful transformations of these strained rings are characterized by predictability, regio- and stereoselectivity [6,7], mild conditions, and broad reaction scope [8,9,10,11,12]. Bis(oxiranes), containing several reactive centers, are structures of particular interest. Generally, they are used to elaborate straightforward approaches to polyfunctionalized compounds [13,14,15], though examples of formation of saturated O-heterocycles via intramolecular cyclization are reported as well [16,17]. An eight-membered ring present in the molecule opens additional synthetic opportunities due to transannular transformations leading to polycyclic structures [18,19].

**Figure 1:**
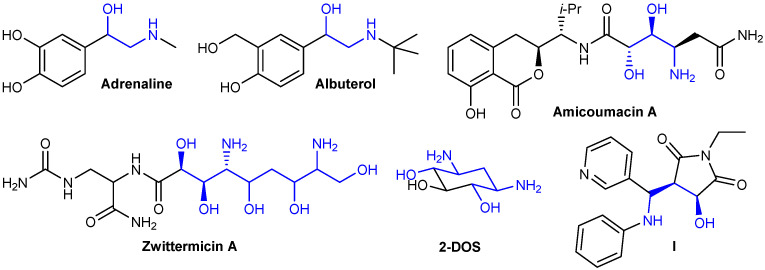
Examples of bioactive compounds containing aminoalcohol scaffolds.

Aminoalcohols and diaminodiols are of interest as scaffolds occurring in a number of bioactive and natural compounds (Figure 1). For instance, adrenalin represents a β-aminoalcohol, as well as a number of adrenergic drugs, such as a short-acting bronchodilator albuterol [20]. Linear aminodiol or aminopolyol moieties are present in natural antibiotics such as amicoumacin A [21] and zwittermicin A [22]. 2-Deoxystreptamine (2-DOS), containing a cyclohexane core, is of importance as a key aminocyclitol scaffold of a number of clinically used aminoglycoside antibiotics (gentamicin, neomycin, etc.) [23]. Conformationally constrained aminoalcohols, such as compound **I**, represent rigid peptidomimetics [24].

**Scheme 1:**
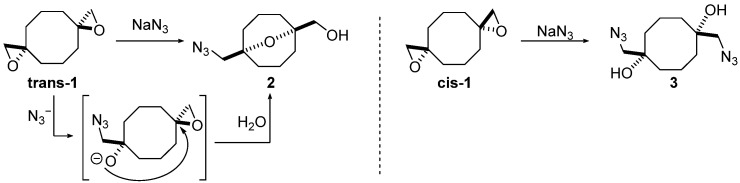
Ring-opening of bis(oxiranes) **trans-1** and **cis-1** with sodium azide [18].

Previously, we have reported the first example of transannular reactions of spirocyclic bis(oxirane) **trans-1** as a nucleophile (Scheme 1) [25], which was later expanded for a tetrakis(oxirane) [26]. The reactivity of two diastereomers of bis(oxirane) **trans-1** and **cis-1** towards NaN_3_ was investigated and it was shown to be determined by the configuration of oxirane moieties: while **trans-1** afforded 9-oxabicyclo[3.3.1]nonane **2**, formed via domino process, including opening of one oxirane ring followed by intramolecular cyclization, diastereomer **cis-1** gave the sole isomer of diazidodiol **3**, derived from independent ring-opening of two oxirane moieties.

**Figure 2:**
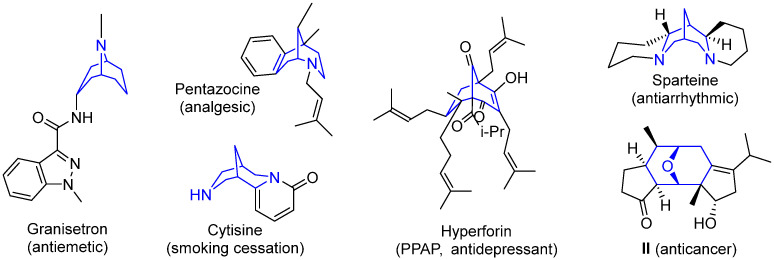
Examples of medicinal drugs and bioactive natural compounds containing bicyclo[3.3.1]nonane and related scaffolds.

It should be mentioned that molecules containing cleft-shaped frameworks such as bicyclo[3.3.1]nonanes and their aza-derivatives have found application as conformationally restricted molecules for the purposes of medicinal chemistry, metal complex catalysis, and the detection of metal ions and small molecules [27]. Natural and synthetic derivatives of aza- and diazabicyclo[3.3.1]nonanes (granisetron, pentazocine, cytisine) are used as medicinal drugs (Figure 2) [28,29,30]. Numerous natural compounds containing fragments of bicyclo[3.3.1]nonane (for example, polycyclic polyprenylated acylphloroglucinols (PPAPs) [31,32]), 2-azabicyclo[3.3.1]nonane (morphine alkaloids [33]), 9-azabicyclo[3.3.1]nonane (granate [34] and bis(indole) alkaloids [35]), 3,7-diazabicyclo[3.3.1]nonane (bispidine-based alkaloids [36]), as well as synthetic bicyclo[3.3.1]nonanes, reveal a broad spectrum of biological activity, including anticancer properties [37,38,39,40].

9-Oxabicyclo[3.3.1]nonane moiety, as well as similar frameworks, represents a conformationally restricted 3D-scaffold attractive for drug design and occurring in bioactive compound. For example, diterpenoid **II** (Figure 2) exhibits antiproliferative activity towards cancer cells with good selectivity compared to normal cell lines [41]. At the same time, in contrast to the above-mentioned bicyclononane derivatives, 9-oxabicyclo[3.3.1]nonanes are rather hard to synthesize and much less examples of them are described; that makes the search for straightforward approaches to these compounds a challenging task.

As for diazidodiol **3**, it represents an attractive structure to be used as a linker moiety for construction of conjugates for biomedical applications using azide–alkyne cycloaddition strategies [42,43]. It was successfully used to obtain bis(triazoles) [44] and bis(steroid) derivatives with anticancer activity [45].

In the present work, we aimed to study the nucleophilic ring-opening of bis(oxiranes) **trans-1** and **cis-1** upon treatment with various amines in order to elaborate preparative approaches to aminoalcohols and diaminodiols of previously unknown structural types.

## 2. Results and Discussion

**Scheme 2:**
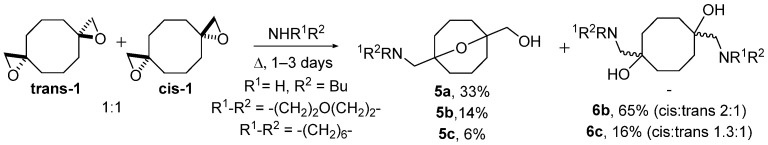
Ring-opening of bis(oxiranes) **trans-1** and **cis-1** with *n*-butylamine, morpholine, and azepane; amines used as solvent.

A diastereomeric mixture of bis(oxiranes) **trans-1** and **cis-1** was studied upon the treatment with *n*-butylamine, morpholine, azepane, and aniline (Scheme 2). The reactions were performed under reflux in the medium of an amine without a solvent. In the case of *n*-butylamine, 9-oxabicyclo[3.3.1]nonane **5a**, derived from transannular reaction of **trans-1**, was the only product. Cis-bis(oxirane) did not interact with butylamine. Reaction of **trans-1** and **cis-1** with more nucleophilic morpholine and azepane besides oxabicyclononane derivatives **5b**,**c** afforded symmetric diaminodiols **6b**,**c**, the products of independent ring-opening. Diaminodiols **6b**,**c** were obtained as diastereomeric mixtures where cis-isomers prevailed. The configuration of isomers **6b**,**c** was established based on different symmetries of molecules as previously performed for the starting bis(oxiranes) [25]. Aniline did not interact with bis(oxiranes) **trans-1** and **cis-1** in these conditions. The obtained preliminary results were in good correlation with the nucleophilicity of amines: secondary amines interacted with both **trans-1** and **cis-1**; less nucleophilic butylamine interacted only with **trans-1**, able to undergo quick intramolecular reaction; the least nucleophilic aniline reacted with neither of the diastereomers.

In order to involve a broader scope of amines into the reaction and to improve its yield and selectivity, the conditions of nucleophilic opening were optimized on the examples of morpholine and butylamine. The solvent, temperature, time, and reagent ratios were varied (see Table S1). In the absence of basic or acidic additives, the reaction proceeded slowly even in acetonitrile under reflux, and an attempt to employ K_2_CO_3_ as a base led to complete decomposition of organic material. The use of LiClO_4_, chosen because of its ability to promote oxiranes’ ring-opening by acting as Lewis acid with non-nucleophilic anion [46], provided almost complete conversion of **trans-1** and **cis-1** at room temperature, though it required three days in the case of morpholine. Optimal reaction conditions were found to be reflux in acetonitrile for 5 h. Various quantities of LiClO_4_ were required, depending on the structure of amine: reactions with butylamine required 10-fold excess per oxirane ring, while for more nucleophilic morpholine, 1.2-fold excess per oxirane ring was enough for the complete conversion of starting bis(oxiranes). It should be mentioned that in optimized conditions, only cis-isomers of diaminodiols **6** were formed, i.e., only transannular reaction proceeded for **trans-1a**.

Bis(oxiranes) **trans-1** and **cis-1** were treated with various amines under optimized conditions (Table 1). In most cases, the diastereomeric mixture of starting bis(oxiranes) was involved into the reaction, with the ratio of starting diastereomers **trans-1**:**cis-1** varying from 1:0.8 to 1:0.9; in some cases, when the products were hard to separate, either pure **trans-1** or **cis-1** was used as the starting material. Bis(oxirane) **trans-1** smoothly reacted with primary and secondary amines, affording aminoalcohols **5a**–**k** of 9-oxabicyclo[3.3.1]nonane series. Aniline, as well as EDG-substituted *p*-toluidine and *p*-anisidine, afforded the products of transannular reaction **5l**–**n** after reflux for 5–10 h, while the reaction with *p*-bromoaniline gave the product **5o** only after reflux for 30 h. Bis(oxirane) **cis-1** also interacted with aliphatic and aromatic amines; yet, unfortunately, the resulting diaminodiols **6** were hard to isolate due to low chromatographic mobility and the tendency to form salts. Nevertheless, diaminodiols **6a**–**f** could be isolated via column chromatography in moderate to high yields. Reaction of **cis-1** with *p*-bromoaniline even after 40 h afforded an equimolar mixture of diaminodiol **6o** and 7-{[(4-bromophenyl)amino]methyl}-1-oxaspiro[2.7]decan-7-ol (**7o**), which was the product of ring-opening of one oxirane moiety. Amines with stronger electron-acceptor substituents, namely, *p*-nitroaniline, sulfanilamide, and methanesulfonamide, did not act as nucleophiles towards bis(oxiranes) **trans-1** and **cis-1** in the described conditions.

To summarize, a preparative method of ring-opening of spirocyclic bis(oxiranes) **trans-1** and **cis-1** upon treatment with various amines in the presence of LiClO_4_ was elaborated to yield a series of aminoalcohols with 9-oxabicyclo[3.3.1]nonane core and substituted cis-cyclooctane-1,5-diols, containing two fragments of amine. The products of the ring-opening of bis(oxirane) **trans-1**, functionalized 9-oxabicyclo[3.3.1]nonanes, represent a valuable conformationally restricted scaffold for drug design, while the ring-opening of bis(oxirane) **cis-1** with bioactive amines may be used as a stereoselective approach to bivalent ligands with a hydrophobic linker.

## 3. Materials and Methods

### 3.1. General

^1^H and ^13^C NMR spectra were recorded on a 400 MHz spectrometer Agilent 400-MR (Agilent Technologies, Santa Clara, CA, USA; 400.0, 100.6 or 376.3 MHz for ^1^H, ^13^C or ^19^F, respectively) at r.t. in CDCl_3_, if not stated otherwise; chemical shifts *δ* were measured with reference to CDCl_3_ (*δ*_H_ = 7.26 ppm, *δ*_C_ = 77.16 ppm) or to CFCl_3_. When necessary, assignments of signals in NMR spectra were made using 2D techniques. Accurate mass measurements (HRMS) were obtained on Bruker micrOTOF II (Bruker Daltonik GmbH, Bremen, Germany) or G3 QTof quadrupole-time-of-flight (Waters, Milford, MA, USA) with electrospray ionization (ESI). Analytical thin layer chromatography was carried out with silica gel plates supported on aluminum (ALUGRAM^®^ Xtra SIL G/UV_254_, Macherey-Nagel, Duren, Germany); the detection was performed by UV lamp (254 nm). Column chromatography was performed on silica gel (Silica 60, 0.015–0.04 mm, Macherey-Nagel, Duren, Germany). Bis(oxiranes) **trans-1** and **cis-1** [25] were obtained via the described method. All other starting materials were commercially available. All reagents except commercial products of satisfactory quality were purified according to literature procedures prior to use.

### 3.2. Reaction of Bis(oxiranes) trans-1 and cis-1 with Amines (General Method)

To a solution of bis(oxirane) (0.1 mmol, 17 mg) in dry CH_3_CN (3 mL), LiClO_4_ (0.5–2 mmol) and corresponding amine (0.22 mmol) were added. The mixture was stirred at 80 °C for 5–40 h. The solvent was evaporated under reduced pressure. The product was isolated via preparative column chromatography (SiO_2_).

**{5-[(Butylamino)methyl]-9-oxabicyclo[3.3.1]nonan-1-yl}methanol (5a).** Reaction time—5 h. Reagent ratio (bis(oxirane):amine:LiClO_4_)—1:2.2:20. Yield 81% (20 mg), yellow oil, R*_f_* 0.20 (light petrol:EtOAc:MeOH 3:1:0.1). ^1^H NMR (*δ*, ppm, *J*, Hz): 0.91 (t, 3H, ^3^*J* = 7.3, CH_3_), 1.27–1.39 (m, 4H, 2CH_2_, cy-Oct + CH_2_, Bu), 1.39–1.57 (m, 4H, 2CH_2_, cy-Oct + CH_2_, Bu), 1.58–1.75 (m, 6H, 6CH_2_, cy-Oct), 1.91–2.08 (m, 2H, 2CH_2_, cy-Oct), 2.54 (s, 2H, CH_2_N), 2.61–2.69 (m, 2H, CH_2_N, Bu), 3.11 (br.s, 2H, OH + NH), 3.31 (s, 2H, CH_2_OH). ^13^C NMR (*δ*, ppm): 14.1 (CH_3_), 18.5 (2CH_2_, cy-Oct), 20.6 (CH_2_, Bu), 29.8 (2CH_2_, cy-Oct), 31.4 (CH_2_, Bu), 31.8 (2CH_2_, cy-Oct), 50.2 (CH_2_N, Bu), 61.2 (CH_2_N), 71.4 (CH_2_OH), 71.7 (C-CH_2_N), 72.5 (C-CH_2_O). HRMS (ESI^+^, *m*/*z*): calculated for C_14_H_27_NO_2_ [M + H]^+^: 242.2115, found: 242.2122.

**[5-(Morpholin-4-ylmethyl)-9-oxabicyclo[3.3.1]nonan-1-yl]methanol (5b).** Reaction time—5 h. Reagent ratio (bis(oxirane):amine:LiClO_4_)—1:2.2:5. Yield 88% (22 mg), orange oil, R*_f_* 0.20 (light petrol:EtOAc:MeOH 3:1:0.1). ^1^H NMR (*δ*, ppm): 1.28–1.46 (m, 4H, 4CH_2_, cy-Oct), 1.57–1.78 (m, 6H, 6CH_2_, cy-Oct), 1.94–2.11 (m, 2H, 2CH_2_, cy-Oct), 2.23 (s, 2H, CH_2_N), 2.37 (br.s, 1H, OH), 2.51–2.62 (m, 4H, 2CH_2_N, morpholine), 3.28 (s, 2H, CH_2_OH), 3.64–3.73 (m, 4H, 2CH_2_O, morpholine). ^13^C NMR (*δ*, ppm): 18.8 (2CH_2_, cy-Oct), 29.8 (2CH_2_, cy-Oct), 31.9 (2CH_2_, cy-Oct), 55.7 (2CH_2_N, morpholine), 67.2 (2CH_2_O, morpholine), 70.0 (CH_2_N), 71.6 (CH_2_OH), 72.1 (C-CH_2_O), 73.7 (C-CH_2_N). HRMS (ESI^+^, *m*/*z*): calculated for C_14_H_25_NO_3_ [M + H]^+^: 256.1907, found: 256.1906.

**[5-(Azepan-1-ylmethyl)-9-oxabicyclo[3.3.1]nonan-1-yl]methanol (5c).** Reaction time—5 h. Reagent ratio (bis(oxirane):amine:LiClO_4_)—1:2.2:5. Yield 54% (15 mg), yellow oil, R*_f_* 0.26 (light petrol:DCM:MeOH 1:3:1). ^1^H NMR (*δ*, ppm): 1.31–1.47 (m, 4H, 4CH_2_, cy-Oct), 1.51–1.73 (m, 14H, 6CH_2_, cy-Oct + 4CH_2_, azepane), 1.93–2.09 (m, 2H, 2CH_2_, cy-Oct), 2.41 (s, 2H, CH_2_N), 2.73–2.83 (m, 4H, 2CH_2_N, azepane), 3.29 (s, 2H, CH_2_OH), 4.62 (br.s, 1H, OH). ^13^C NMR (*δ*, ppm): 18.9 (2CH_2_, cy-Oct), 27.5 (2CH_2_, azepane), 29.1 (2CH_2_, azepane), 30.0 (2CH_2_, cy-Oct), 31.7 (2CH_2_, cy-Oct), 57.9 (2CH_2_N, azepane), 69.2 (CH_2_N), 71.79 (CH_2_OH), 71.83 (C-CH_2_OH), 74.4 (C-CH_2_N). HRMS (ESI^+^, *m*/*z*): calculated for C_16_H_29_NO_2_ [M + H]^+^: 268.2271, found: 268.2272.

**[5-(Piperidin-1-ylmethyl)-9-oxabicyclo[3.3.1]nonan-1-yl]methanol (5d).** Reaction time—5 h. Reagent ratio (bis(oxirane):amine:LiClO_4_)—1:2.2:5. Yield 78% (13 mg), yellow oil, R*_f_* 0.14 (light petrol: DCM:MeOH 1:3:0.5). ^1^H NMR (*δ*, ppm): 1.29–1.48 (m, 4H, 4CH_2_, cy-Oct + 2H, CH_2_, piperidine), 1.49–1.81 (m, 6H, 6CH_2_, cy-Oct + 4H, 2CH_2_, piperidine), 1.95–2.11 (m, 2H, 2CH_2_, cy-Oct), 2.14–2.34 (m, 2H, CH_2_N), 2.54 (br.s, 4H, 2CH_2_N, piperidine), 3.29 (s, 2H, CH_2_O). ^13^C NMR (*δ*, ppm): 18.9 (2CH_2_, cy-Oct), 24.1 (CH_2_, piperidine), 26.1 (2CH_2_, piperidine), 29.9 (2CH_2_, cy-Oct), 32.0 (2CH_2_, cy-Oct), 56.8 (2CH_2_N, piperidine), 70.2 (CH_2_N), 71.7 (CH_2_OH), 72.0 (C-CH_2_OH), 73.6 (C-CH_2_N). HRMS (ESI^+^, *m*/*z*): calculated for C_15_H_27_NO_2_ [M + H]^+^: 254.2115, found: 254.2119.

**[5-(Pyrrolidin-1-ylmethyl)-9-oxabicyclo[3.3.1]nonan-1-yl]methanol (5e).** Reaction time—5 h. Reagent ratio (bis(oxirane):amine:LiClO_4_)—1:2.2:5. Yield 83% (20 mg), yellow oil, R*_f_* 0.24 (light petrol:DCM:MeOH 1:4:1). ^1^H NMR (*δ*, ppm): 1.32–1.40 (m, 2H, 2CH_2_, cy-Oct), 1.44–1.52 (m, 2H, 2CH_2_, cy-Oct), 1.58–1.76 (m, 6H, 6CH_2_, cy-Oct), 1.78–1.92 (m, 4H, 2CH_2_, pyrrolidine), 1.96–2.13 (m, 2H, 2CH_2_, cy-Oct), 2.56 (br.s, 2H, CH_2_N), 2.82 (br.s, 4H, 2CH_2_N, pyrrolidine), 3.32 (br.s, 2H, 2CH_2_OH, pyrrolidine). ^13^C NMR (*δ*, ppm): 18.8 (2CH_2_, cy-Oct), 23.8 (2CH_2_, pyrrolidine), 29.6 (2CH_2_, cy-Oct), 32.1 (2CH_2_, cy-Oct), 56.5 (2CH_2_, pyrrolidine), 68.1 (CH_2_N), 71.5 (CH_2_OH), 72.3 (C-CH_2_N), 72.8 (C-CH_2_OH). HRMS (ESI^+^, *m*/*z*): calculated for C_14_H_25_NO_2_ [M + H]^+^:240.1958, found: 240.1961.

**1,5-Bis[(dibutylamino)methyl]cyclooctane-1,5-diol (5f).** Reaction time—5 h. Reagent ratio (bis(oxirane):amine:LiClO_4_)—1:2.2:5. Yield 83% (25 mg), yellow oil, R*_f_* 0.16 (EtOAc). ^1^H NMR (*δ*, ppm, *J*, Hz): 0.89 (t, ^3^*J* = 7.2, 6H, 2CH_3_, Bu), 1.20–1.31 (m, 4H, 2CH_2_, Bu), 1.31–1.45 (m, 8H, 4CH_2_, cy-Oct + 2CH_2_, Bu), 1.56–1.77 (m, 6H, 6CH_2_, cy-Oct), 1.95–2.08 (m, 2H, 2CH_2_, cy-Oct), 2.30 (s, 2H, CH_2_N), 2.44–2.57 (m, 4H, 2CH_2_N, Bu), 3.28 (s, 2H, CH_2_O). ^13^C NMR (*δ*, ppm): 14.3 (2CH_3_, Bu), 18.9 (2CH_2_, cy-Oct), 20.8 (2CH_2_, Bu), 29.5 (2CH_2_, Bu), 29.8 (2CH_2_, cy-Oct), 31.8 (2CH_2_, cy-Oct), 56.1 (2CH_2_N, Bu), 66.3 (CH_2_N), 71.7 (CH_2_O), 72.1 (C-CH_2_O), 73.8 (C-CH_2_N). HRMS (ESI^+^, *m*/*z*): calculated for C_18_H_35_NO_2_ [M + H]^+^: 298.2741, found: 298.2725.

**{5-[(Propargylamino)methyl]-9-oxabicyclo[3.3.1]nonan-1-yl}methanol (5g).** Reaction time—5 h. Reagent ratio (bis(oxirane):amine:LiClO_4_)—1:2.2:20. Yield 70% (16 mg), yellow oil, R*_f_* 0.32 (light petrol:EtOAc:MeOH 1:1:1). ^1^H NMR (*δ*, ppm, *J*, Hz): 1.30–1.40 (m, 2H, 2CH_2_, cy-Oct), 1.41–1.52 (m, 2H, 2CH_2_, cy-Oct), 1.57–1.74 (m, 6H, 6CH_2_, cy-Oct), 1.95–2.10 (m, 2H, 2CH_2_, cy-Oct), 2.28 (t, ^4^*J* = 2.4, 1H, CH, propargyl), 2.67 (s, 2H, CH_2_N), 3.06 (br.s, 2H, NH, OH), 3.35 (s, 2H, CH_2_O), 3.54 (d, ^4^*J* = 2.4 Hz, CH_2_, propargyl). ^13^C NMR (*δ*, ppm): 18.4 (2CH_2_, cy-Oct), 29.7 (2CH_2_, cy-Oct), 31.6 (2CH_2_, cy-Oct), 38.5 (CH_2_, propargyl), 59.8 (CH_2_N), 71.3 (CH_2_OH), 71.7 (C-CH_2_N), 72.6 (C-CH_2_O), 72.8 (CH, propargyl), 80.9 (C, propargyl). HRMS (ESI^+^, *m*/*z*): calculated for C_13_H_21_NO_2_ [M + H]^+^: 224.1645, found: 224.1649.

**{5-[(Benzylamino)methyl]-9-oxabicyclo[3.3.1]nonan-1-yl}methanol (5h).** Reaction time—5 h. Reagent ratio (bis(oxirane):amine:LiClO_4_)—1:2.2:20. Yield 80% (22 mg), yellow oil, R*_f_* 0.39 (light petrol:EtOAc:MeOH 1:1:0.5). ^1^H NMR (*δ*, ppm): 1.27–1.36 (m, 2H, 2CH_2_, cy-Oct), 1.37–1.50 (m, 2H, 2CH_2_, cy-Oct), 1.54–1.71 (m, 6H, 4CH_2_, cy-Oct), 1.89–2.03 (m, 2H, 2CH_2_, cy-Oct), 2.57 (s, 2H, CH_2_N), 3.31 (s, 2H, CH_2_O), 4.00 (s, 2H, PhCH_2_N), 4.10 (br.s, 2H, NH+OH), 7.28–7.41 (m, 5H, 5CH, Ph). ^13^C NMR (*δ*, ppm): 18.5 (2CH_2_, cy-Oct), 29.7 (2CH_2_, cy-Oct), 31.7 (2CH_2_, cy-Oct), 53.7 (PhCH_2_N), 60.0 (CH_2_N), 71.5 (CH_2_OH), 71.8 (C), 72.6 (C), 127.5 (CH, Ph), 128.7 (4CH, Ph), 138.7 (C, Ph). HRMS (ESI^+^, *m*/*z*): calculated for C_17_H_25_NO_2_ [M + H]^+^: 276.1958, found: 276.1965.

**(5-{[(4-Ethylbenzyl)amino]methyl}-9-oxabicyclo[3.3.1]nonan-1-yl)methanol (5i).** Reaction time—5 h. Reagent ratio (bis(oxirane):amine:LiClO_4_)—1:2.2:20. Yield 65% (20 mg), yellow oil, R*_f_* 0.78 (light petrol:DCM:MeOH 1:1:0.5). ^1^H NMR (*δ*, ppm, *J*, Hz): 1.23 (t, 3H, ^3^*J* 7.6, CH_3_), 1.25–1.34 (m, 2H, 2CH_2_, cy-Oct), 1.38–1.49 (m, 2H, 2CH_2_, cy-Oct), 1.56–1.74 (m, 6H, 6CH_2_, cy-Oct), 1.91–2.06 (m, 2H, 2CH_2_, cy-Oct), 2.55 (s, 2H, CH_2_N), 2.64 (q, 2H, ^3^*J* 7.6, CH_2_, Et), 3.26 (s, 2H, CH_2_O), 3.77 (br.s, 2H, NH+OH), 3.93 (s, 2H, ArCH_2_N), 7.15–7.20 (m, 2H, 2CH, Ar), 7.30–7.36 (m, 2H, 2CH, Ar). ^13^C NMR (*δ*, ppm): 15.7 (CH_3_), 18.4 (2CH_2_, cy-Oct), 28.7 (CH_2_, Et), 29.6 (2CH_2_, cy-Oct), 31.7 (2CH_2_, cy-Oct), 53.3 (ArCH_2_N), 59.5 (CH_2_N), 71.2 (CH_2_OH), 71.3 (C-CH_2_N), 72.8 (C-CH_2_OH), 128.3 (2CH, Ar), 129.0 (2CH, Ar), 134.4 (C, Ar), 143.9 (C, Ar). HRMS (ESI^+^, *m*/*z*): calculated for C_19_H_29_NO_2_ [M + H]^+^: 304.2271, found: 304.2272.

**[5-({[4-(Trifluoromethyl)benzyl])amino}methyl)-9-oxabicyclo[3.3.1]nonan-1-yl]methanol (5j).** Reaction time—10 h. Reagent ratio (bis(oxirane):amine:LiClO_4_)—1:2.2:20. Yield 40% (13 mg), yellow oil, R*_f_* 0.24 (light petrol:EtOAc 1:1). ^1^H NMR (*δ*, ppm, *J*, Hz): 1.31–1.46 (m, 4H, 4CH_2_, cy-Oct), 1.55–1.78 (m, 6H, 6CH_2_, cy-Oct), 1.93–2.09 (m, 2H, 2CH_2_, cy-Oct), 2.47 (s, 2H, CH_2_N), 3.31 (s, 2H, CH_2_O), 3.87 (s, 2H, ArCH_2_N), 7.41–7.50 (m, 2H, 2CH, Ar), 7.54–7.60 (m, 2H, 2CH, Ar). ^13^C NMR (*δ*, ppm, *J*, Hz): 18.6 (2CH_2_, cy-Oct), 29.9 (2CH_2_, cy-Oct), 31.8 (2CH_2_, cy-Oct), 53.7 (ArCH_2_N), 61.0 (CH_2_N), 71.6 (CH_2_OH), 72.3 (C), 72.4 (C), 124.5 (q, ^1^*J*_CF_ 272), 125.4 (q, ^3^*J*_CF_ 4, 2CH, Ar), 128.3 (2CH, Ar), 129.3 (q, ^2^*J*_CF_ 32, C, Ar), 145.0 (C, Ar). ^19^F NMR (*δ*, ppm): −62.37 (s, 3F). HRMS (ESI^+^, *m*/*z*): calculated for C_18_H_24_F_3_NO_2_ [M + H]^+^: 344.1832, found: 344.1836.

**[5-({[4-(3-Methylpiperidin-1-yl)benzyl]amino}methyl)-9-oxabicyclo[3.3.1]non-1-yl]methanol (5k).** Reaction time—5 h. Reagent ratio (bis(oxirane):amine:LiClO_4_)—1:2.2:20. Yield 16% (6 mg), yellow oil, R*_f_* 0.78 (light petrol:DCM:MeOH 1:1:0.5). ^1^H NMR (*δ*, ppm, *J*, Hz): 0.94 (d, 3H, ^3^*J* 6.6, CH_3_), 0.99–1.13 (m, 1H, CH_2_, piperidine), 1.19–1.35 (m, 2H, cy-Oct), 1.40–1.51 (m, 2H, 2CH_2_, cy-Oct), 1.50–1.87 (m, 10H, 6CH_2_, cy-Oct + 2CH_2_ piperidine + CH, piperidine), 1.88–2.09 (m, 2H, 2CH_2_, cy-Oct), 2.31–2.41 (m, 1H, CH_2_N, piperidine), 2.60–2.73 (m, 1H, CH_2_N, piperidine), 2.76 (s, 2H, CH_2_N), 3.34 (s, 2H, CH_2_O), 3.54–3.69 (m, 2H, 2CH_2_N, piperidine), 4.26 (s, 2H, ArCH_2_N), 6.86–6.96 (m, 2H, 2CH, Ar), 7.36–7.38 (d, 2H, 2CH, Ar), 7.89 (br.s, 2H, NH+OH). ^13^C NMR (*δ*, ppm): 18.1 (2CH_2_, cy-Oct), 19.6 (CH_3_), 25.2 (CH_2_, piperidine), 28.9 (2CH_2_, cy-Oct), 30.9 (CH, piperidine), 31.3 (2CH_2_, cy-Oct), 33.0 (CH_2_, piperidine), 49.2 (CH_2_, piperidine), 51.7 (ArCH_2_N), 55.7 (CH_2_N), 56.9 (CH_2_, piperidine), 69.4 (C-CH_2_N), 70.7 (CH_2_OH), 73.8 (C-CH_2_OH), 116.1 (2CH, Ar), 118.9 (C, Ar), 131.6 (2CH, Ar), 152.5 (C, Ar). HRMS (ESI^+^, *m*/*z*): calculated for C_23_H_36_N_2_O_2_ [M + H]^+^: 373.2850, found: 373.2852.

**{5-[(Phenylamino)methyl]-9-oxabicyclo[3.3.1]nonan-1-yl}methanol (5l).** Reaction time—5 h. Reagent ratio (bis(oxirane):amine:LiClO_4_)—1:2.2:20. Yield 36% (9 mg), yellow oil, R*_f_* 0.77 (light petrol:EtOAc 1:2). ^1^H NMR (*δ*, ppm): 1.35–1.43 (m, 2H, 2CH_2_, cy-Oct), 1.44–1.53 (m, 2H, 2CH_2_, cy-Oct), 1.59–1.79 (m, 6H, 4CH_2_, cy-Oct), 1.97–2.11 (m, 2H, 2CH_2_, cy-Oct), 3.03 (s, 2H, CH_2_N), 3.35 (s, 2H, CH_2_O), 6.58–6.73 (m, 3H, 3CH, Ph), 7.12–7.19 (m, 2H, 2CH, Ph). ^13^C NMR (101 MHz, CDCl_3_) *δ*, ppm: 18.5 (2CH_2_, cy-Oct), 29.8 (2CH_2_, cy-Oct), 31.6 (2CH_2_, cy-Oct), 55.4 (CH_2_N), 71.5 (CH_2_OH), 72.4 (C), 72.5 (C), 113.0 (2CH, Ph), 117.3 (CH, Ph), 129.3 (2CH, Ph), 148.9 (C, Ph). HRMS (ESI^+^, *m*/*z*): calculated for C_16_H_23_NO_2_ [M + H]^+^: 262.1802, found: 262.1806.

**{5-[(4-Methoxyphenylamino)methyl]-9-oxabicyclo[3.3.1]nonan-1-yl}methanol (5m).** Reaction time—5 h. Reagent ratio (bis(oxirane):amine:LiClO_4_)—1:2.2:20. Yield 95% (27 mg), yellow oil, R*_f_* 0.32 (EtOAc:DCM 1:5). ^1^H NMR (*δ*, ppm): 1.34–1.42 (m, 2H, 2CH_2_, cy-Oct), 1.43–1.53 (m, 2H, 2CH_2_, cy-Oct), 1.59–1.81 (m, 6H, 6CH_2_, cy-Oct), 1.96–2.10 (m, 2H, 2CH_2_, cy-Oct), 2.97 (s, 2H, CH_2_N), 3.35 (s, 2H, CH_2_O), 3.74 (s, 3H, OMe), 6.56–6.64 (m, 2H, 2CH, Ar), 6.74–6.80 (m, 2H, 2CH, Ar). ^13^C NMR (*δ*, ppm): 18.5 (2CH_2_, cy-Oct), 29.9 (2CH_2_, cy-Oct), 31.7 (2CH_2_, cy-Oct), 56.0 (OMe), 56.5 (CH_2_N), 71.6 (CH_2_O), 72.4 (C-CH_2_NH), 72.5 (C-CH_2_OH), 114.3 (2CH, Ar), 115.1 (2CH, Ar), 143.3 (C-NH, Ar), 152.1 (C-O, Ar). HRMS (ESI^+^, *m*/*z*): calculated for C_17_H_25_NO_3_ [M + H]^+^: 292.1907, found: 292.1911.

**{5-[(4-Methylphenylamino)methyl]-9-oxabicyclo[3.3.1]nonan-1-yl}methanol (5n).** Reaction time—10 h. Reagent ratio (bis(oxirane):amine:LiClO_4_)—1:2.2:20. Yield 26% (7 mg), yellow oil, R*_f_* 0.67 (light petrol:EtOAc 1:1). ^1^H NMR (*δ*, ppm): 1.32–1.42 (m, 2H, 2CH_2_, cy-Oct), 1.43–1.53 (m, 2H, 2CH_2_, cy-Oct), 1.57–1.82 (m, 6H, 4CH_2_, cy-Oct), 1.95–2.11 (m, 2H, 2CH_2_, cy-Oct), 2.23 (s, 3H, CH_3_), 3.00 (s, 2H, CH_2_N), 3.35 (s, 2H, CH_2_O), 6.52–6.58 (m, 2H, 2CH, Ar), 6.93–7.01 (m, 2H, 2CH, Ar). ^13^C NMR (*δ*, ppm): 18.5 (2CH_2_, cy-Oct), 20.5 (CH_3_), 29.8 (2CH_2_, cy-Oct), 31.6 (2CH_2_, cy-Oct), 55.7 (CH_2_N), 71.6 (CH_2_OH), 72.4 (C), 72.5 (C), 113.1 (2CH, Ar), 126.4 (C, Ar), 129.8 (2CH, Ar), 146.7 (C, Ar). HRMS (ESI^+^, *m*/*z*): calculated for C_17_H_25_NO_2_ [M + H]^+^: 276.1958, found: 276.1966.

**{5-[(4-Bromophenylamino)methyl]-9-oxabicyclo[3.3.1]nonan-1-yl}methanol (5o).** Reaction time—30 h. Reagent ratio (bis(oxirane):amine:LiClO_4_)—1:2.2:20. Yield 17% (6 mg), yellow oil, R*_f_* 0.58 (light petrol:EtOAc 1:1). ^1^H NMR (*δ*, ppm): 1.34–1.43 (m, 2H, 2CH_2_, cy-Oct), 1.43–1.52 (m, 2H, 2CH_2_, cy-Oct), 1.58–1.75 (m, 6H, 4CH_2_, cy-Oct), 1.97–2.12 (m, 2H, 2CH_2_, cy-Oct), 2.98 (s, 2H, CH_2_N), 3.35 (s, 2H, CH_2_O), 6.45–6.54 (m, 2H, 2CH, Ar), 7.19–7.26 (m, 2H, 2CH, Ar). ^13^C NMR (*δ*, ppm): 18.5 (2CH_2_, cy-Oct), 29.8 (2CH_2_, cy-Oct), 31.6 (2CH_2_, cy-Oct), 55.3 (CH_2_N), 71.6 (CH_2_OH), 72.4 (C), 72.6 (C), 108.7 (C, Ar), 114.5 (2CH, Ar), 132.0 (2CH, Ar), 148.0 (C, Ar). HRMS (ESI^+^, *m*/*z*): calculated for C_16_H_22_BrNO_2_ [M + H]^+^: 340.0907, found: 340.0916.

**1,5-Bis[(butylamino)methyl]cyclooctane-1,5-diol (6a).** Reaction time—5 h. Reagent ratio (bis(oxirane):amine:LiClO_4_)—1:2.2:20. Yield 56% (19 mg), yellow oil, R*_f_* 0.05 (CH_3_CN). ^1^H NMR (*δ*, ppm, *J*, Hz): 0.90 (t, 6H, ^3^*J* 7.2, 2CH_3_), 1.29–1.38 (m, 4H, 2CH_2_, Bu), 1.39–1.50 (m, 10H, 6CH_2_, cy-Oct + 2CH_2_, Bu), 1.77–1.90 (m, 6H, 6CH_2_, cy-Oct), 2.46 (s, 4H, 2CH_2_N), 2.62 (t, 4H, ^3^*J* 7.1, 2CH_2_N, Bu). ^13^C NMR (*δ*, ppm): 14.1 (2CH_3_, Bu), 18.8 (2CH_2_, cy-Oct), 20.5 (2CH_2_, Bu), 32.6 (2CH_2_, Bu_),_ 37.5 (4CH_2_, cy-Oct), 50.5 (2CH_2_N, Bu), 60.5 (2CH_2_N), 72.8 (2C). HRMS (ESI^+^, *m*/*z*): calculated for C_18_H_38_N_2_O_2_ [M + H]^+^: 315.3006, found: 315.3015.

**1,5-Bis(morpholin-4-ylmethyl)cyclooctane-1,5-diol (6b).** Reaction time—5 h. Reagent ratio (bis(oxirane):amine:LiClO_4_)—1:2.2:5. Yield 97% (33 mg), yellow oil, R*_f_* = 0.61 (light petrol:EtOAc:MeOH 3:1:1). ^1^H NMR (*δ*, ppm): 1.37–1.54 (m, 6H, 6CH_2_, cy-Oct), 1.79–1.95 (m, 6H, 6CH_2_, cy-Oct), 2.26 (s, 4H, 2CH_2_N), 2.60–2.63 (m, 8H, 4CH_2_N, morpholine), 2.99 (br.s, 2H, 2OH), 3.68–3.71 (m, 8H, 4CH_2_O, morpholine). ^13^C NMR (*δ*, ppm): 18.4 (2CH_2_, cy-Oct), 37.9 (4CH_2_, cy-Oct), 56.3 (4CH_2_N, morpholine), 67.5 (4CH_2_O, morpholine), 69.0 (2CH_2_N), 74.0 (2C). HRMS (ESI^+^, *m*/*z*): calculated for C_18_H_34_N_2_O_4_ [M + H]^+^: 343.2591, found: 343.2596.

**1,5-Bis(azepan-4-ylmethyl)cyclooctane-1,5-diol (6c).** Reaction time—5 h. Reagent ratio (bis(oxirane):amine:LiClO_4_)—1:2.2:5. Yield 19% (7 mg), yellow oil, R*_f_* 0.08 (light petrol:DCM:MeOH 1:3:1). ^1^H NMR (*δ*, ppm): 1.38–1.52 (m, 6H, 6CH_2_, cy-Oct), 1.53–1.71 (m, 16H, 8CH_2_, azepane), 1.78–1.96 (m, 6H, 6CH_2_, cy-Oct), 2.43 (s, 4H, 2CH_2_N), 2.74–2.86 (m, 8H, 4CH_2_N, azepane). ^13^C NMR (*δ*, ppm): 18.7 (2CH_2_, cy-Oct), 27.1 (4CH_2_, azepane), 29.1 (4CH_2_, azepane), 37.8 (4CH_2_, cy-Oct), 59.2 (4CH_2_N, azepane), 68.8 (2CH_2_N), 73.7 (2C). HRMS (ESI^+^, *m*/*z*): calculated for C_22_H_42_N_2_O_2_ [M + H]^+^: 367.3319, found: 367.3319.

**1,5-Bis(piperidin-4-ylmethyl)cyclooctane-1,5-diol (6d).** Reaction time—5 h. Reagent ratio (bis(oxirane):amine:LiClO_4_)—1:2.2:5. Yield 97% (30 mg), yellow oil, R*_f_* 0.09 (light petrol:DCM:MeOH 1:3:2). ^1^H NMR (*δ*, ppm): 1.35–1.53 (m, 6H, 6CH_2_, cy-Oct + 4H, 2CH_2_, piperidine), 1.53–1.65 (m, 8H, 4CH_2,_ piperidine), 1.79–1.95 (m, 6H, 6CH_2_, cy-Oct), 2.29 (s, 4H, 2CH_2_N), 2.61 (br.s, 8H, 4CH_2_N, piperidine). ^13^C NMR (*δ*, ppm): 18.5 (2CH_2_, cy-Oct), 23.9 (2CH_2_, piperidine), 26.3 (4CH_2_, piperidine), 38.0 (4CH_2_, cy-Oct), 57.4 (4CH_2_N, piperidine), 68.6 (2CH_2_N), 73.3 (2C). HRMS (ESI^+^, *m*/*z*): calculated for C_20_H_38_N_2_O_2_ [M + H]^+^: 339.3006, found 339.3003.

**1,5-Bis(pyrrolidin-4-ylmethyl)cyclooctane-1,5-diol (6e).** Reaction time—5 h. Reagent ratio (bis(oxirane):amine:LiClO_4_)—1:2.2:5. Yield 61% (23 mg), yellow oil, R*_f_* 0.1 (light petrol:DCM:MeOH 1:1:1). ^1^H NMR (*δ*, ppm): 1.40–1.57 (m, 6H, 6CH_2_, cy-Oct), 1.68–1.79 (m, 8H, 4CH_2_, pyrrolidine), 1.80–1.94 (m, 6H, 6CH_2_, cy-Oct), 2.45 (s, 4H, 2CH_2_N), 2.62–2.74 (m, 8H, 4CH_2_N, pyrrolidine). ^13^C NMR (*δ*, ppm): 18.6 (2CH_2_, cy-Oct), 24.3 (4CH_2_, pyrrolidine), 38.0 (4CH_2_, cy-Oct), 57.0 (4CH_2_, pyrrolidine), 67.2 (2CH_2_N), 73.4 (2C). HRMS (ESI^+^, *m*/*z*): calculated for C_18_H_34_N_2_O_2_ [M + H]^+^: 311.2693, found: 311.2701.

**1,5-Bis[(dibutylamino)methyl]cyclooctane-1,5-diol (6f).** Reaction time—20 h. Reagent ratio (bis(oxirane):amine:LiClO_4_)—1:2.2:5. Yield 65% (28 mg), yellow oil, R*_f_* 0.24 (light petrol:DCM:MeOH 1:4:1). ^1^H NMR (CD_3_OD; *δ*, ppm, *J*, Hz): 0.96 (t, ^3^*J* = 7.3, 12H, 4CH_3_, Bu), 1.25–1.42 (m, 8H, 4CH_2_, Bu), 1.46–1.64 (m, 14H, 6CH_2_, cy-Oct + 4CH_2_, Bu), 1.85–1.98 (m, 6H, 6CH_2_, cy-Oct), 2.64 (br.s, 4H, 2CH_2_N), 2.79 (br.s, 8H, 4CH_2_N, Bu). ^13^C NMR (CD_3_OD; *δ*, ppm): 14.3 (4CH_3_, Bu), 18.8 (2CH_2_, cy-Oct), 21.4 (4CH_2_, Bu), 29.0 (4CH_2_, Bu), 38.4 (4CH_2_, cy-Oct), 57.2 (4CH_2_N, Bu), 66.5 (2CH_2_N), 74.6 (2C). HRMS (ESI^+^, *m*/*z*): calculated for C_26_H_54_N_2_O_2_ [M + H]^+^: 427.4258, found: 427.4282.

**1,5-Bis[(phenylamino)methyl]cyclooctane-1,5-diol (6l).** Reaction time—5 h. Reagent ratio (bis(oxirane):amine:LiClO_4_)—1:2.2:20. Yield 6% (2 mg), yellow oil, R*_f_* 0.58 (light petrol:EtOAc 2:1). ^1^H NMR (*δ*, ppm): 1.56–1.71 (m, 6H, 6CH_2_, cy-Oct), 1.83–1.93 (m, 2H, 2CH_2_, cy-Oct), 1.93–2.04 (m, 4H, 4CH_2_, cy-Oct), 3.05 (s, 4H, 2CH_2_N), 6.59–6.77 (m, 6H, 6CH, Ph), 7.12–7.20 (m, 4H, 4CH, Ph). ^13^C NMR (*δ*, ppm): 18.5 (2CH_2_, cy-Oct), 37.3 (4CH_2_, cy-Oct), 55.9 (2CH_2_, CH_2_N), 74.2 (2C), 113.4 (4CH, Ar), 118.0 (2CH, Ar), 129.4 (4CH, Ar), 148.9 (2C, Ar). HRMS (ESI^+^, *m*/*z*): calculated for C_22_H_30_N_2_O_2_ [M + H]^+^: 355.2380, found: 355.2388.

**1,5-Bis[(4-bromophenylamino)methyl]cyclooctane-1,5-diol (6o).** Reaction time—40 h. Reagent ratio (bis(oxirane):amine:LiClO_4_)—1:2.2:20. Yield 4% (2 mg), yellow oil, R*_f_* 0.32 (light petrol:EtOAc 1:2). ^1^H NMR (*δ*, ppm): 1.50–1.69 (m, 6H, 6CH_2_, cy-Oct), 1.79–1.91 (m, 2H, 2CH_2_, cy-Oct), 1.91–2.02 (m, 4H, 4CH_2_, cy-Oct), 3.00 (s, 4H, 2CH_2_N), 6.49–6.59 (m, 4H, 4CH, Ar), 7.20–7.29 (m, 4H, 4CH, Ph). ^13^C NMR (*δ*, ppm): 18.4 (2CH_2_, cy-Oct), 37.3 (4CH_2_, cy-Oct), 55.8 (2CH_2_, CH_2_N), 74.2 (2C), 109.4 (2C, Ar), 114.9 (4CH, Ar), 132.1 (4CH, Ar), 147.9 (C, Ar). HRMS (ESI^+^, *m*/*z*): calculated for C_22_H_28_Br_2_N_2_O_2_ [M + H]^+^: 511.0590, found: 511.0587.


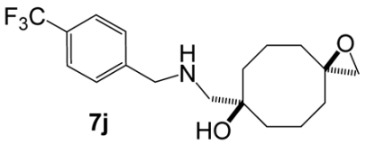


**7-({[(4-Trifluoromethyl)benzyl]amino}methyl)-1-oxaspiro[2.7]decan-7-ol (7j).** Reaction time—10 h. Reagent ratio (bis(oxirane):amine:LiClO_4_)—1:2.2:20. Yield 7% (2 mg), yellow oil, R*_f_* 0.16 (EtOAc). ^1^H NMR (*δ*, ppm): 1.38–1.49 (m, 4H, 2CH_2_, cy-Oct), 1.56–1.87 (m, 8H, 6CH_2_, cy-Oct), 2.55 (s, 2H, CH_2_O), 2.60 (s, 2H, CH_2_N), 3.90 (s, 2H, ArCH_2_N), 7.37–7.48 (m, 2H, 2CH, Ar), 7.54–7.64 (m, 2H, 2CH, Ar). ^13^C NMR (*δ*, ppm): 19.3 (2CH_2_, cy-Oct), 34.9 (2CH_2_, cy-Oct), 35.9 (2CH_2_, cy-Oct), 54.1 (CH_2_N), 55.3 (CH_2_O), 58.1 (ArCH_2_N), 59.1 (C, epoxy), 73.8 (C-OH), 125.5 (q, ^3^*J*_CF_ 4, 2CH, Ar), 128.4 (2CH, Ar). Signals of CF_3_-group and quaternary carbon atoms were not observed due to low concentration of the compound. ^19^F NMR (*δ*, ppm): −62.44 (c, 3F). HRMS (ESI^+^, *m*/*z*): calculated for C_18_H_24_F_3_NO_2_ [M + H]^+^: 344.1832, found: 344.1827.


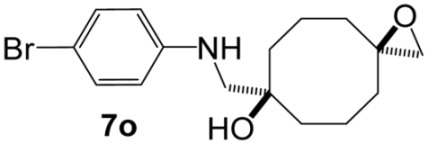


**7-{[(4-Bromophenyl)amino]methyl}-1-oxaspiro[2.7]decan-7-ol (7o).** Reaction time—40 h. Reagent ratio (bis(oxirane):amine:LiClO_4_)—1:2.2:20. Yield 6% (2 mg), yellow oil, R*_f_* 0.39 (light petrol:EtOAc 1:1). ^1^H NMR (*δ*, ppm): 1.47–1.92 (m, 12H, 6CH_2_, cy-Oct), 2.63 (s, 2H, CH_2_O), 3.04 (s, 2H, CH_2_N), 7.48–7.58 (m, 2H, 2CH, Ar), 7.19–7.26 (m, 2H, 2CH, Ar). ^13^C NMR (*δ*, ppm): 19.4 (2CH_2_, cy-Oct), 34.9 (2CH_2_, cy-Oct), 35.8 (2CH_2_, cy-Oct), 53.5 (CH_2_N), 55.5 (CH_2_O), 59.0 (C, epoxy), 75.1 (C-OH), 109.2 (C, Ar), 114.8 (2CH, Ar), 132.1 (2CH, Ar), 147.9 (C, Ar). HRMS (ESI^+^, *m*/*z*): calculated for C_16_H_22_BrNO_2_ [M + H]^+^: 340.0907, found: 340.0915.

## Figures and Tables

**Table 1:** Ring-opening of bis(oxiranes) **trans-1** and **cis-1** with various amines.

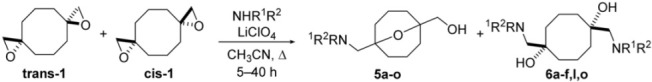					
Starting Material	NHR^1^R^2^	Product	Yield, % ^1^	Product	Yield, % ^1^
**trans-1** + **cis-1**	NH_2_Bu	**5a**	81	**6a**	–
**cis-1**	NH_2_Bu	–	–	**6a**	65
**trans-1** + **cis-1**	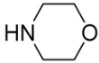	**5b**	88	**6b**	97
**trans-1** + **cis-1**	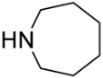	**5c**	54	**6c**	19
**trans-1** + **cis-1**	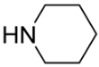	**5d**	83	**6d**	–
**cis-1**	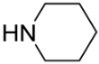	–	–	**6d**	97
**trans-1** + **cis-1**	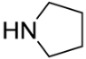	**5e**	83	**6e**	–
**cis-1**	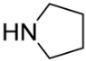	–	–	**6e**	61
**trans-1**	NHBu_2_	**5f**	83	–	–
**cis-1**	NHBu_2_	–	–	**6f**	65
**trans-1** + **cis-1**	HC≡CCH_2_NH_2_	**5g**	70	**6g**	–
**trans-1** + **cis-1**	PhCH_2_NH_2_	**5h**	80	**6h**	–
**trans-1** + **cis-1**	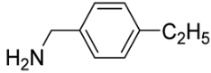	**5i**	65	**6i**	–
**trans-1**	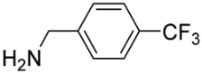	**5j**	40	–	–
**cis-1**	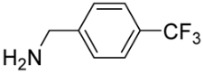	–	–	**6j**	– ^2^
**trans-1** + **cis-1**	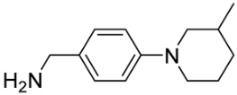	**5k**	16	**6k**	–
**trans-1** + **cis-1**	PhNH_2_	**5l**	36	**6l**	6
**trans-1** + **cis-1**	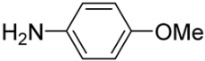	**5m**	95	**6m**	–
**trans-1** + **cis-1**	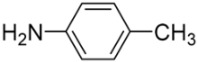	**5n**	26	**6n**	–
**trans-1** + **cis-1**	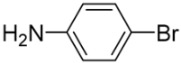	**5o**	17	**6o**	–
**cis-1**	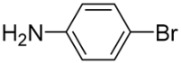	–	–	**6o**	2 ^2^

^1^ Isolated yields, taking in account the ratio of starting diastereomers **trans-1** and **cis-1**. ^2^ The products of opening of one oxirane ring **7j,o** (see Section 3.2) were obtained.

## Data Availability

Data is contained within the article or Supplementary Materials.

## Supplementary Materials

The following supporting information can be downloaded at https://www.mdpi.com/article/10.3390/molecules31020252/s1; Table S1: Optimization of ring-opening conditions; copies of NMR spectra of the obtained compounds.
