# Supplementary material for: Straightforward Synthetic Approach to Aminoalcohols with 9-oxabicyclo[3.3.1]nonane or Cyclooctane Core via Nucleophilic Ring-Opening of Spirocyclic Bis(oxiranes)
Source: Molecules. 2026 Jan 12;31(2):252. doi: 10.3390/molecules31020252 (PMC12843951; doi:10.3390/molecules31020252)

Straightforward synthetic approach to aminoalcohols with 9-oxabicyclo[3.3.1]nonane or cyclooctane core *via* nucleophilic ring-opening of spirocyclic bis(oxiranes)

Olga V. Ryzhikova, Daiana V. Savchenkova, Sergey V. Kositov, Yuri K. Grishin, Olga A. Maloshitskaya, Kseniya N. Sedenkova and Elena B. Averina*

Supplementary information

1. Optimization of ring-opening conditions 2
2. Copies of NMR spectra 3

Table S1. Optimization of ring-opening conditions

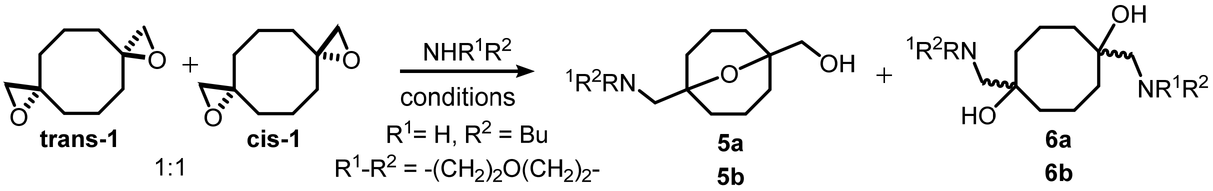

NHR^1R^2	**trans-1** : **cis-1**	equiv. NHR^1R^2	additive	equiv. additive	solvent	T, ˚C	t, h	conversion of **trans-1** and **cis-1**, %	products ratio, **5**:**6**
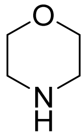	1:1.0	2.2	-	-	toluene	48	27	20	1:0.9
	1:0.9	2.2	-	-	CH_3CN	80	5<br>10	9<br>16	1:1.2<br>1:1.3
	1:1.1	2.2	K_2CO_3	2	CH_3CN	80	24	decomposed	-
	1:0.9	2.2	$LiClO_4$	20	Et_2O	20	1<br>20	0<br>decomposed	-<br>-
	1:1.2	2.2	$LiClO_4$	5	CH_3CN	20	5<br>10<br>72	29<br>47<br>91	1:1.0<br>1:1.1<br>1:1.4
	1:1.2	2.2	$LiClO_4$	1.2	CH_3CN	80	2<br>5<br>10	23<br>41<br>91	1:1.5<br>1:1.2<br>1:1.2
	1:1.2	2.2	$LiClO_4$	1.8	CH_3CN	80	2<br>5<br>10	9<br>88<br>100	1:1.1<br>1:1.1<br>1:1.4
	1:1.2	**2.2**	**$LiClO_4$**	**2.4**	**CH_3CN**	**80**	**5**	**100**	1:1.3
	1:1.2	2.2	$LiClO_4$	5	CH_3CN	80	10<br>22	100<br>decomposed	1:1.2<br>-
	1:0.8	2.2	$LiClO_4$	10	CH_3CN	80	24	100	1:0.6
	1:0.8	20	$LiClO_4$	20	CH_3CN	80	24	100	1:0.9
NH_2Bu	1:0.6	1.1*2	$LiClO_4$	5	CH_3CN	80	5	20	1:0.2
	1:0.6	1.1*2	$LiClO_4$	10	CH_3CN	80	5	65	1:0.4
	1:0.8	**1.1*2**	**$LiClO_4$**	**20**	**CH_3CN**	**80**	**5**	**100**	1:0.6

^{1}H NMR spectrum (400 MHz, $CDCl_3$) of compound **5a**

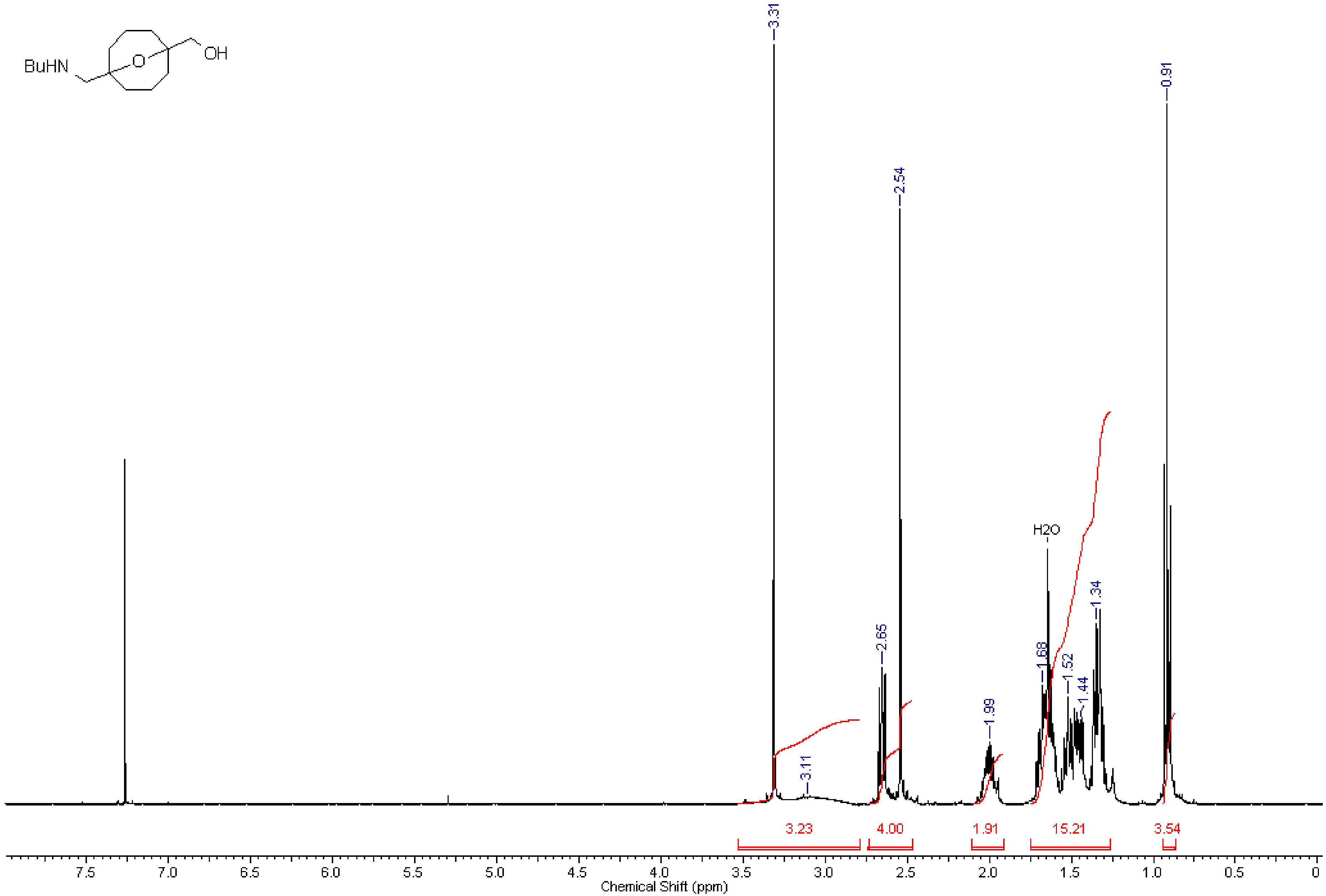

^{13}C NMR spectrum (101 MHz, $CDCl_3$) of compound **5a**

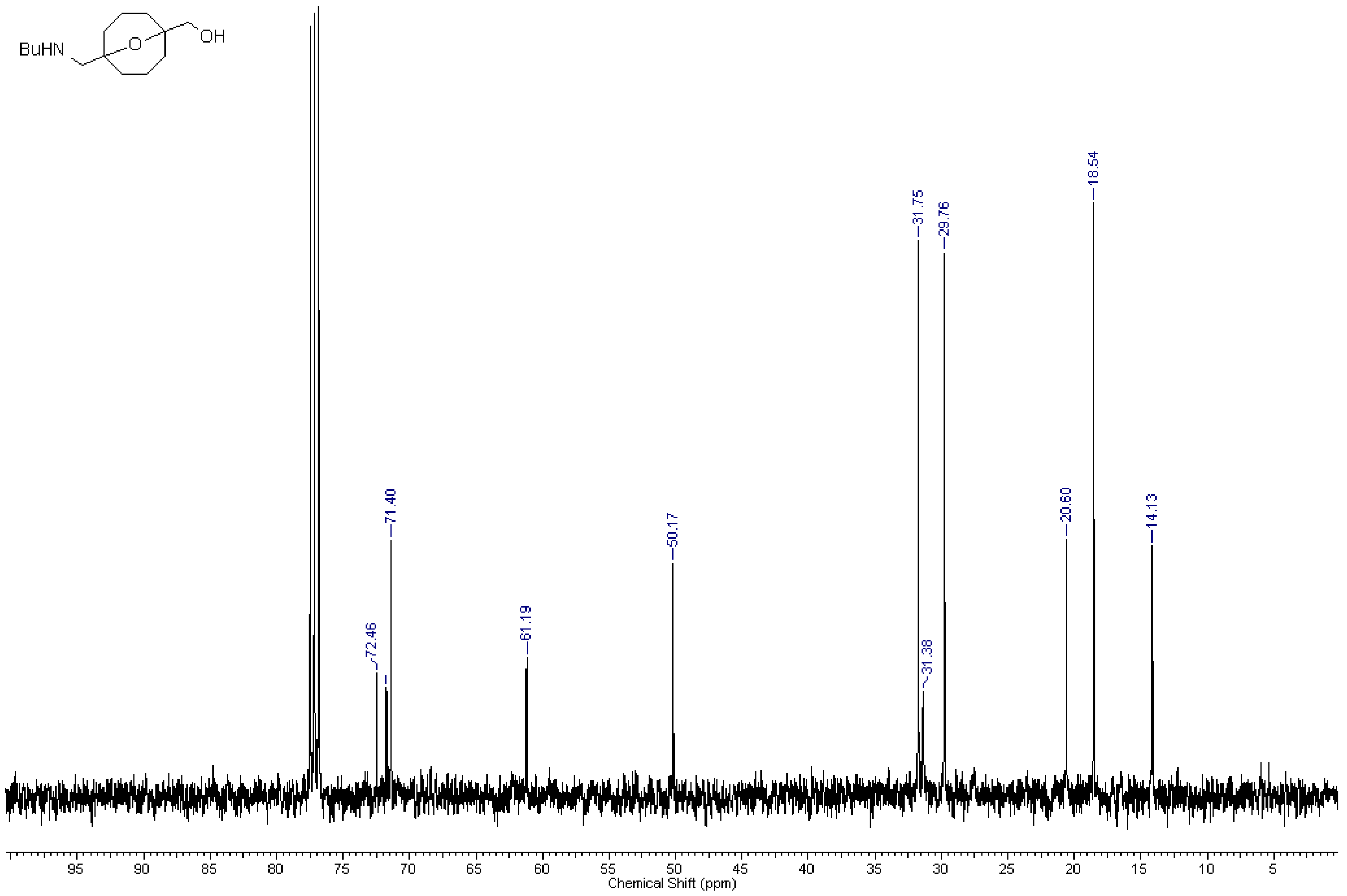

HSQC NMR spectrum of compound **5a**

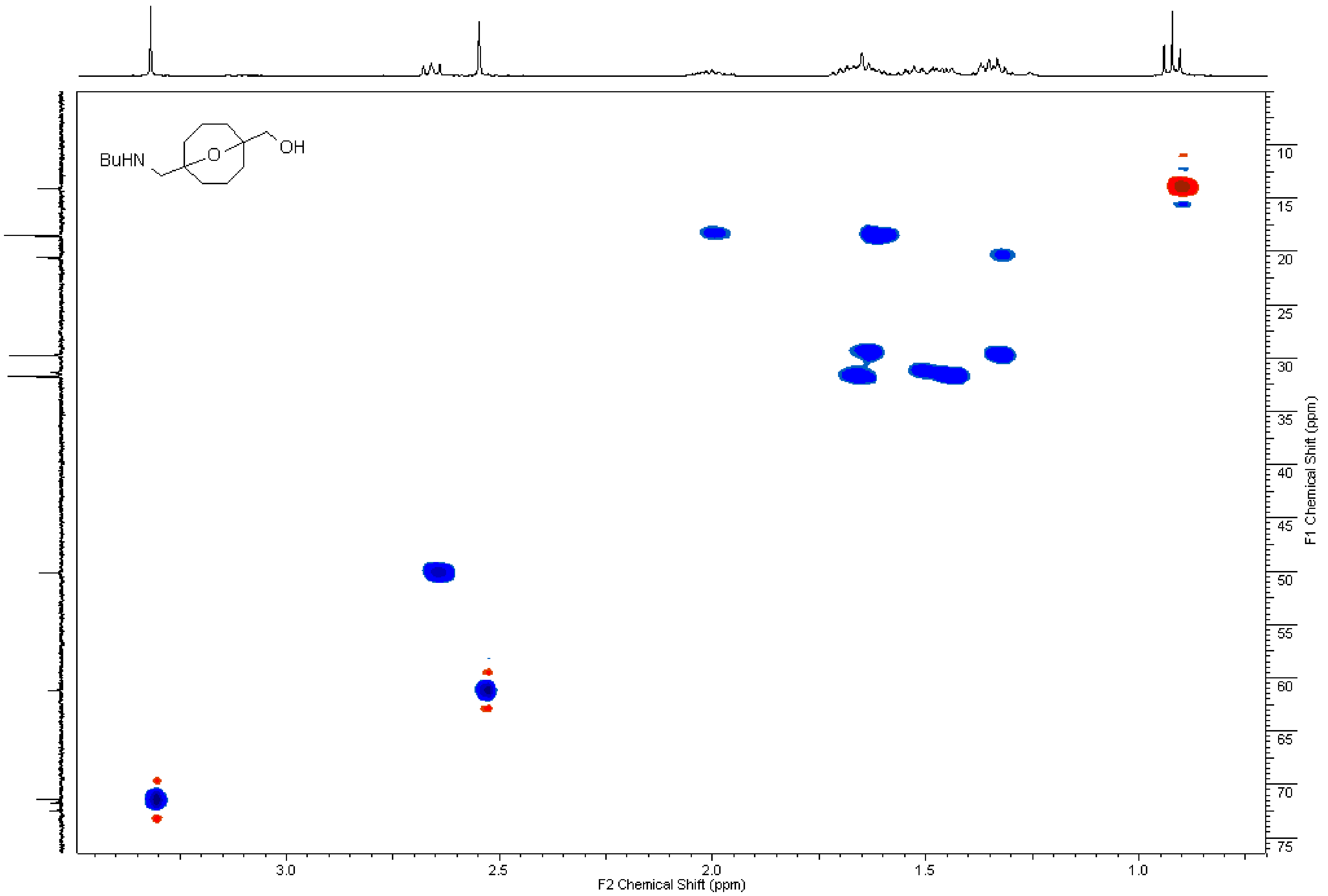

HMBC NMR spectrum of compound **5a**

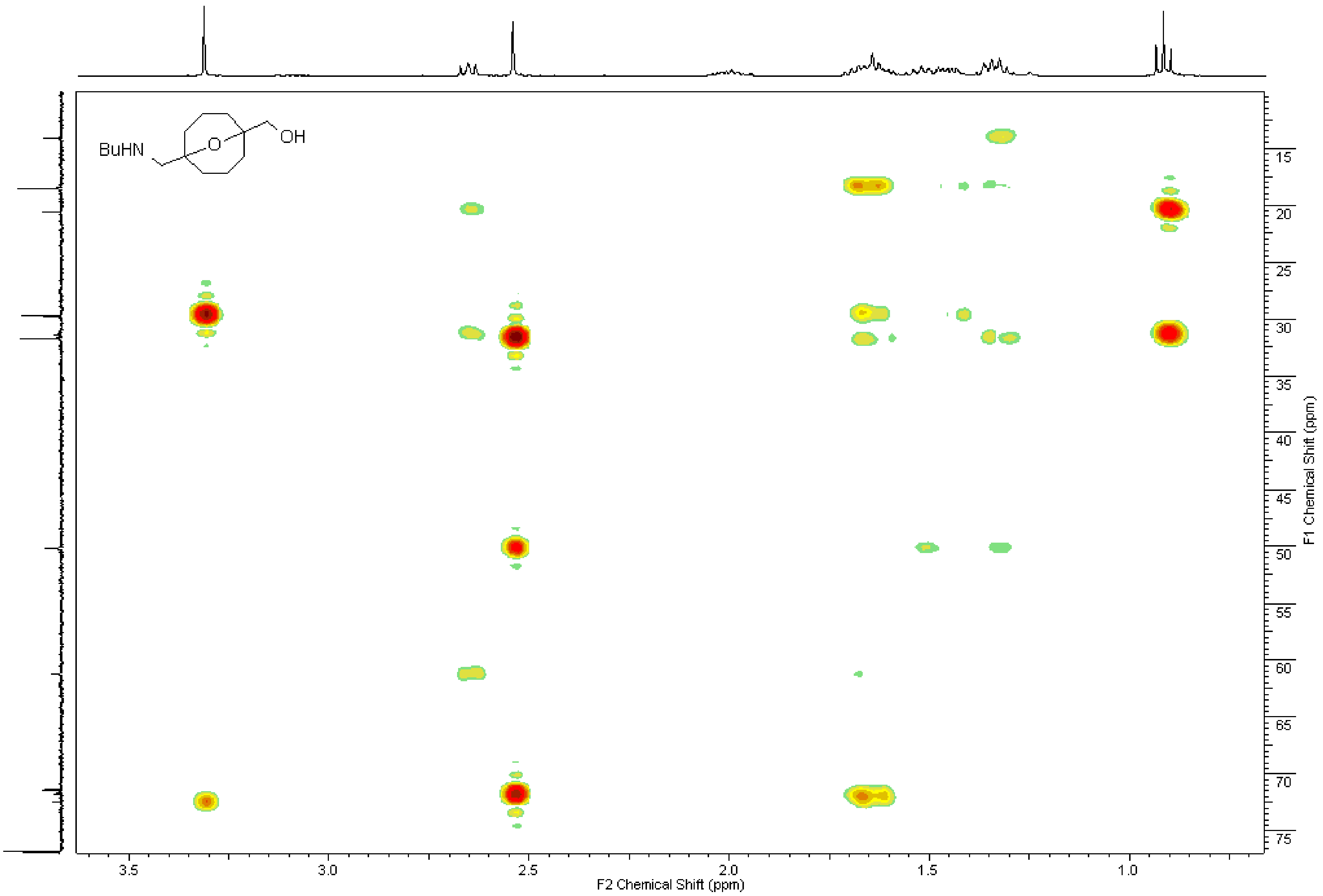

^{1}H NMR spectrum (400 MHz, $CDCl_3$) of compound **5b**

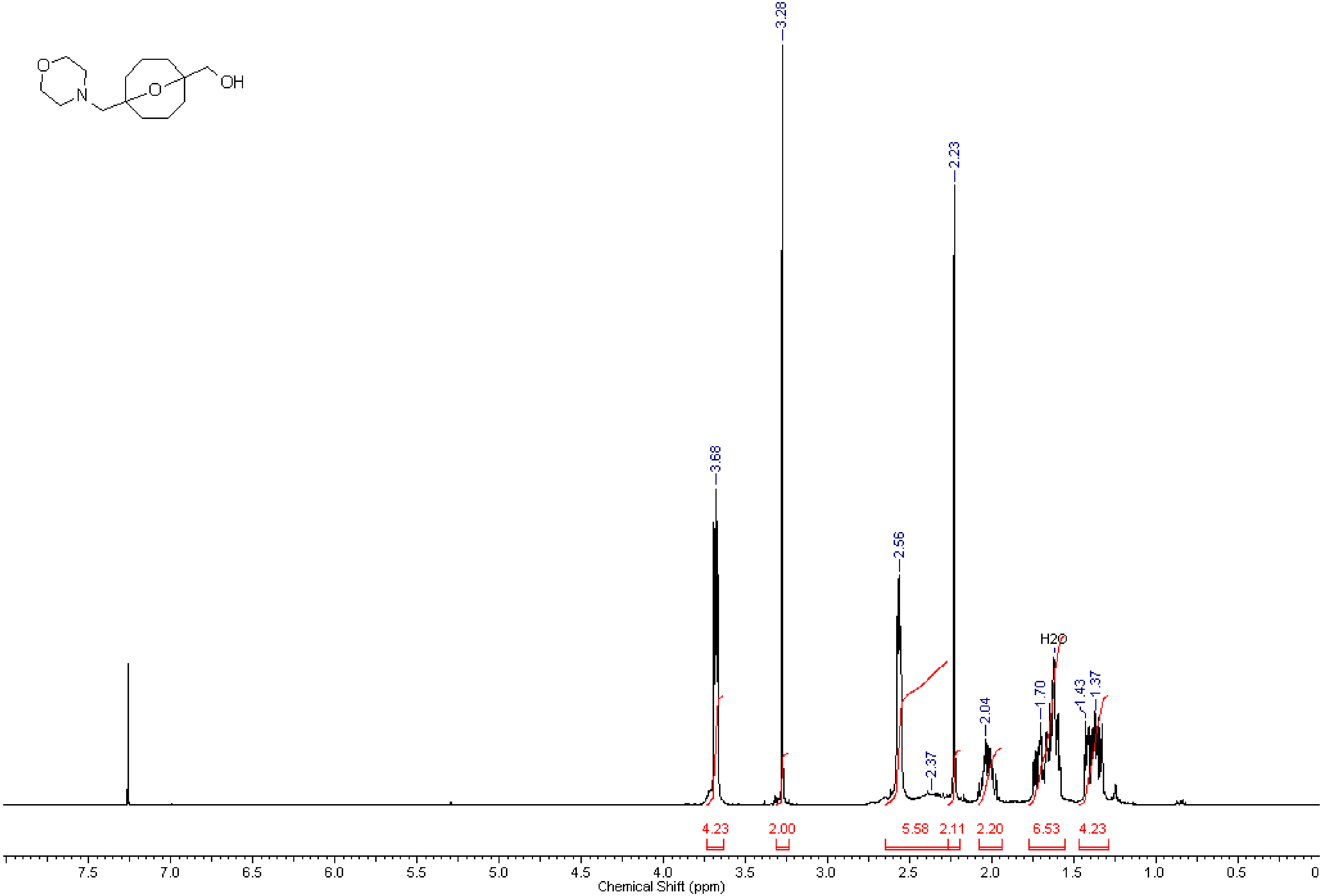

^{13}C NMR spectrum (101 MHz, $CDCl_3$) of compound **5b**

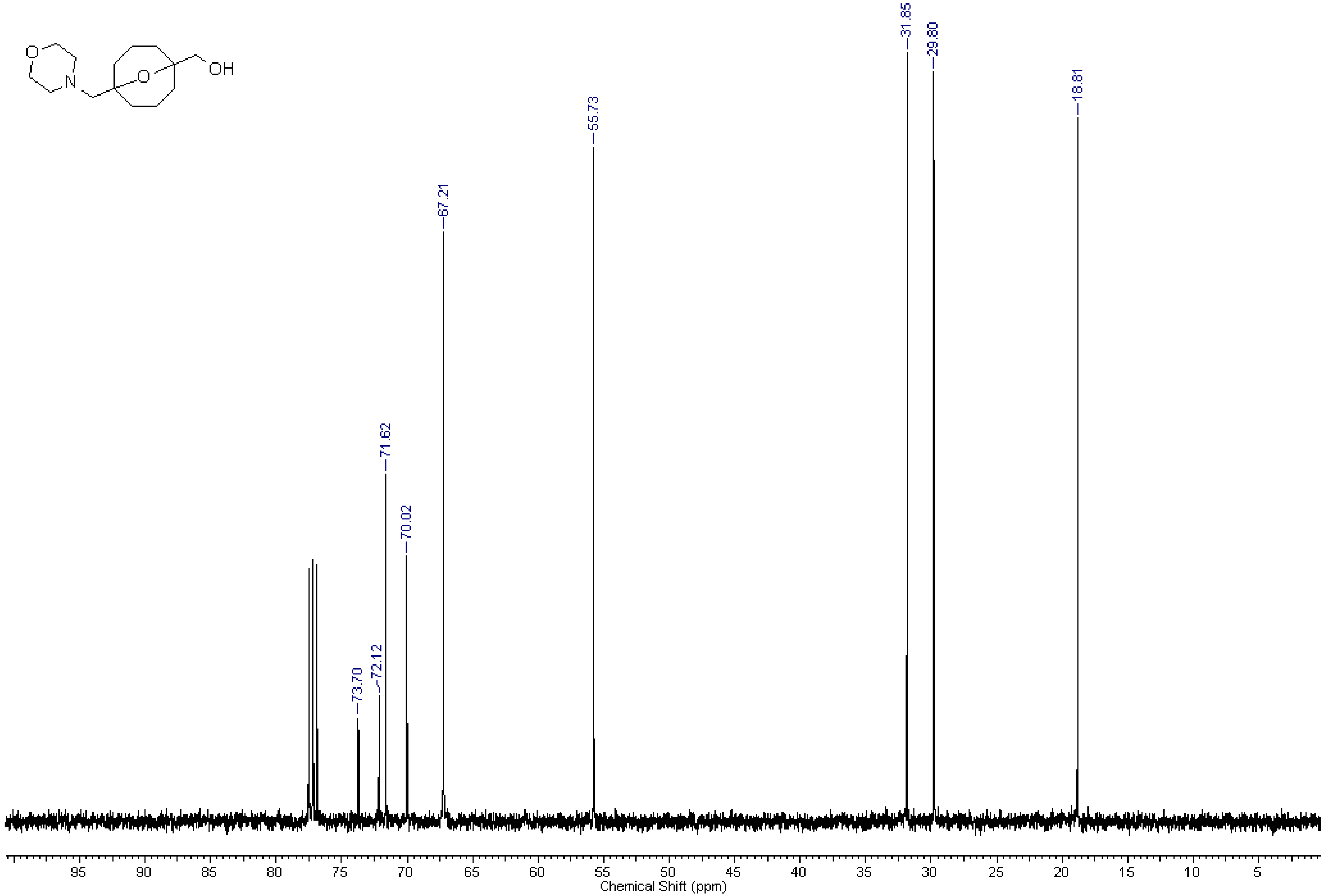

HSQC NMR spectrum of compound **5b**

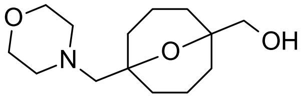

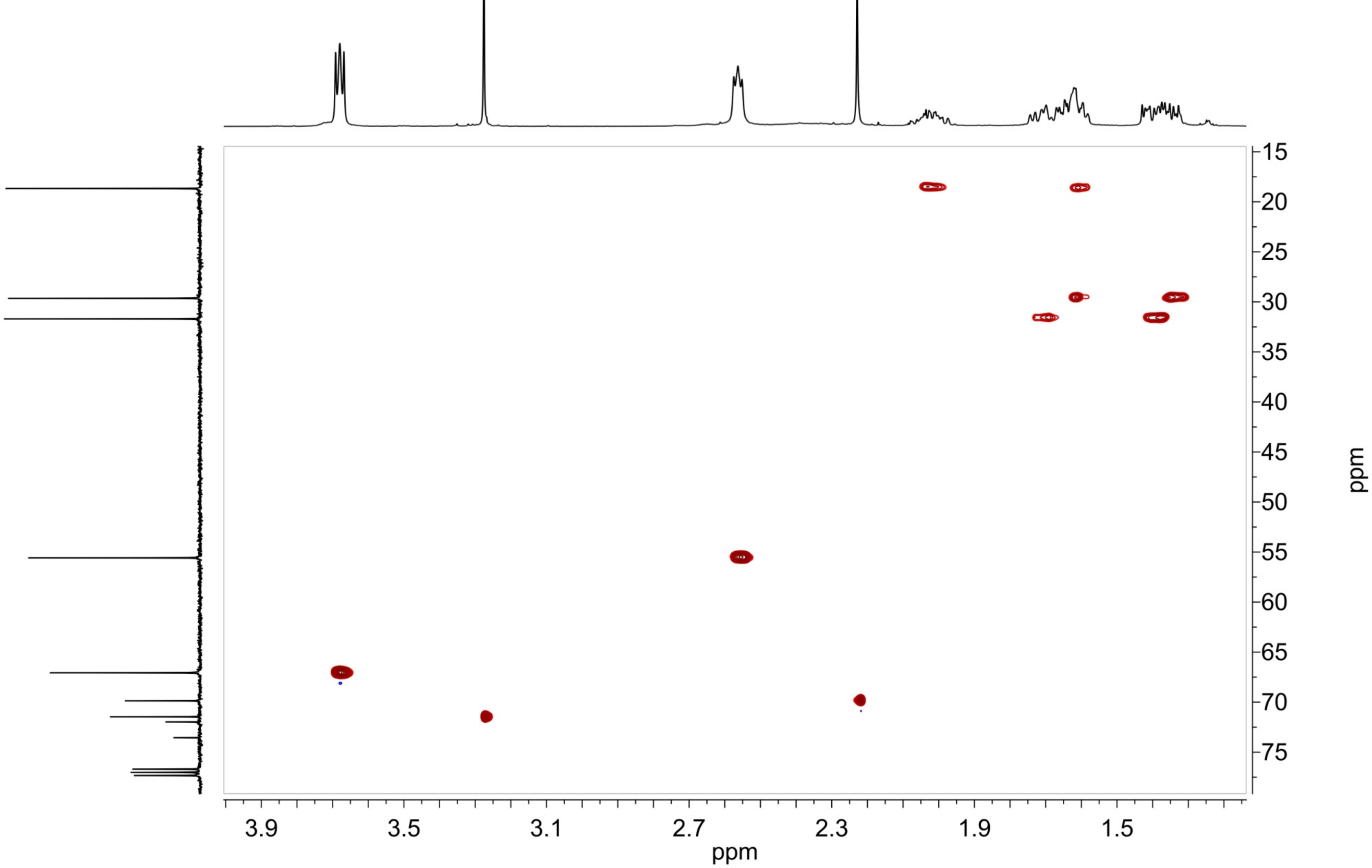

HMBC NMR spectrum of compound **5b**

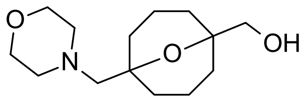

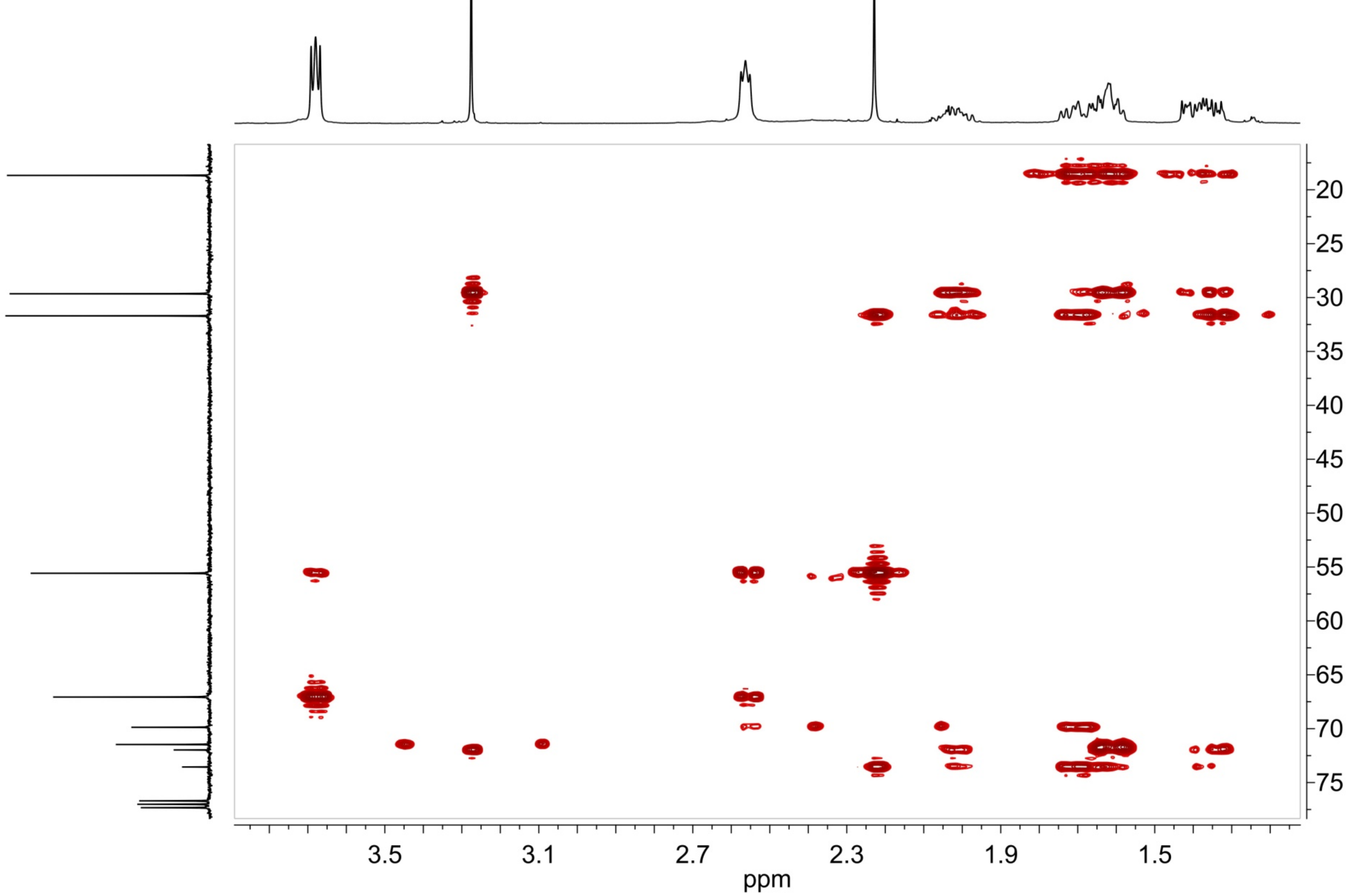

^{1}H NMR spectrum (400 MHz, $CDCl_3$) of compound **5c**

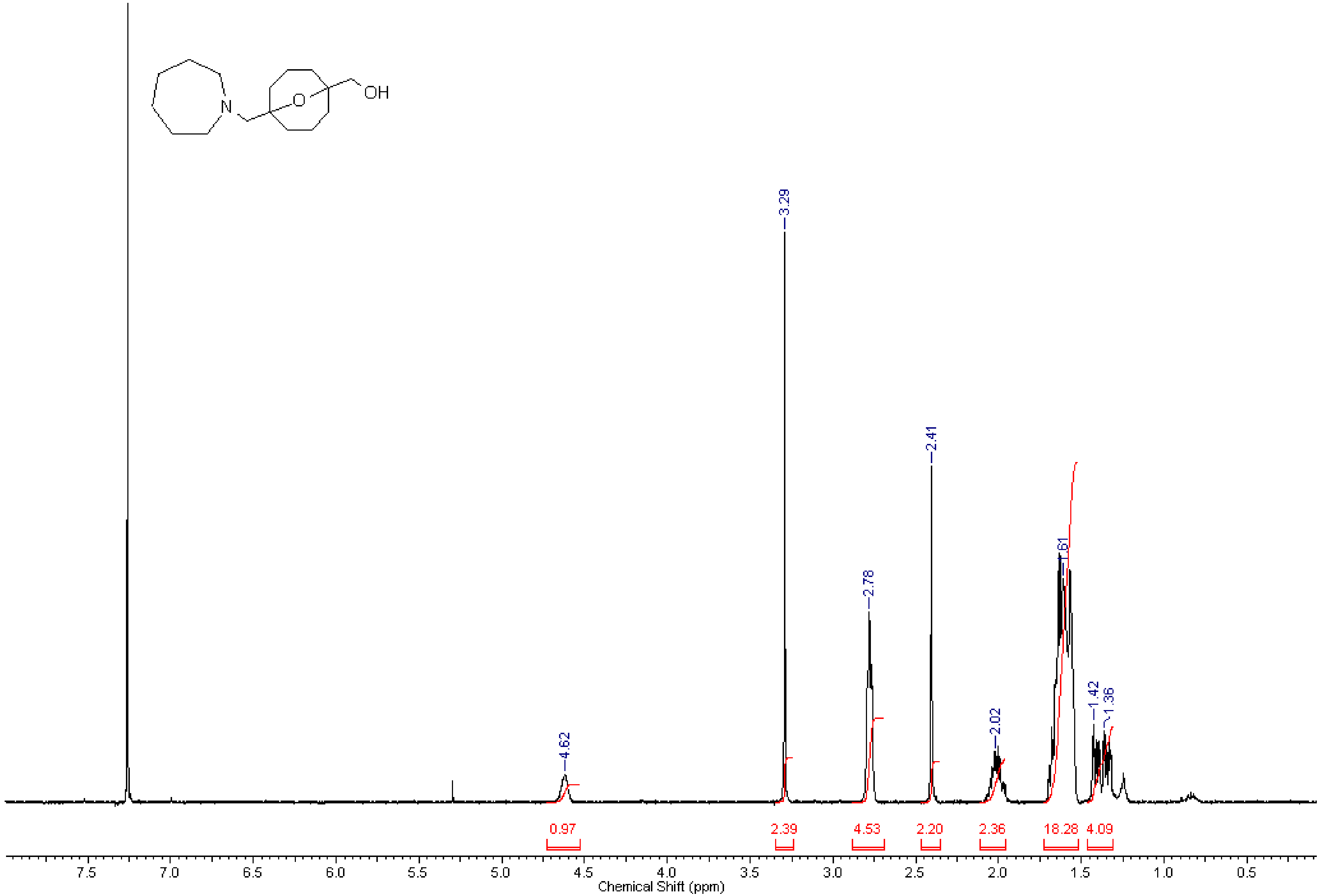

^{13}C NMR spectrum (101 MHz, $CDCl_3$) of compound **5c**

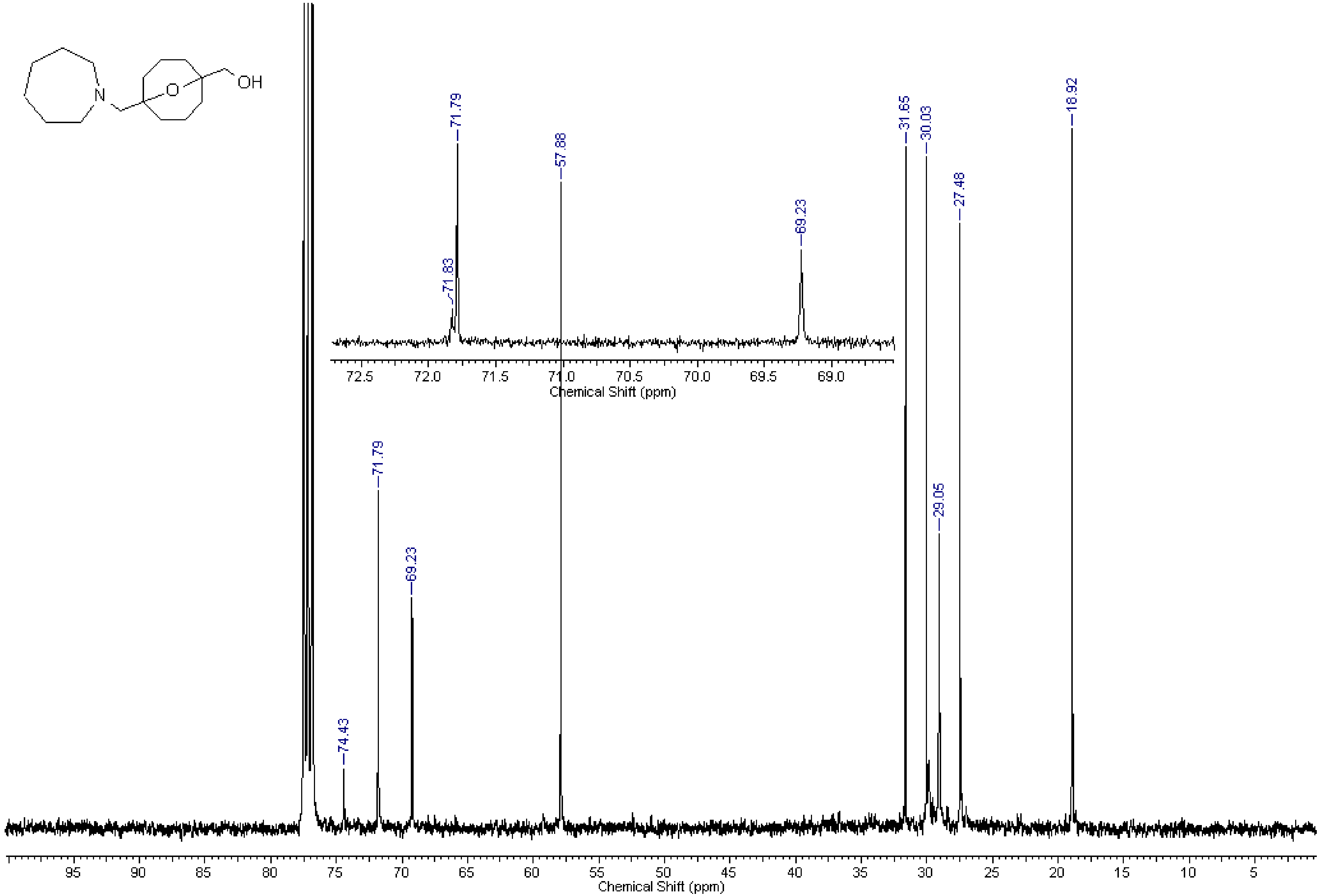

HSQC NMR spectrum of compound **5c**

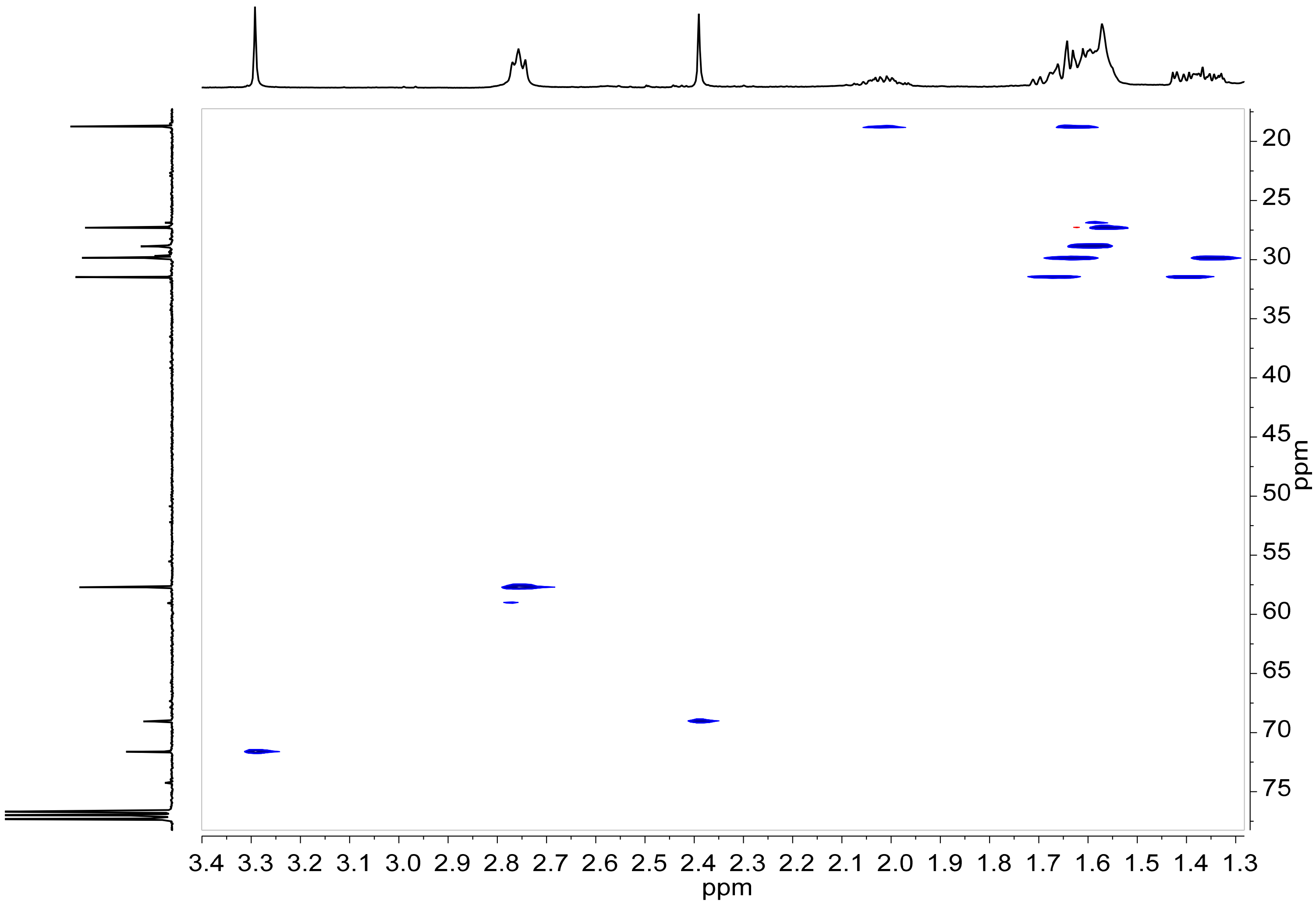

HMBC NMR spectrum of compound **5c**

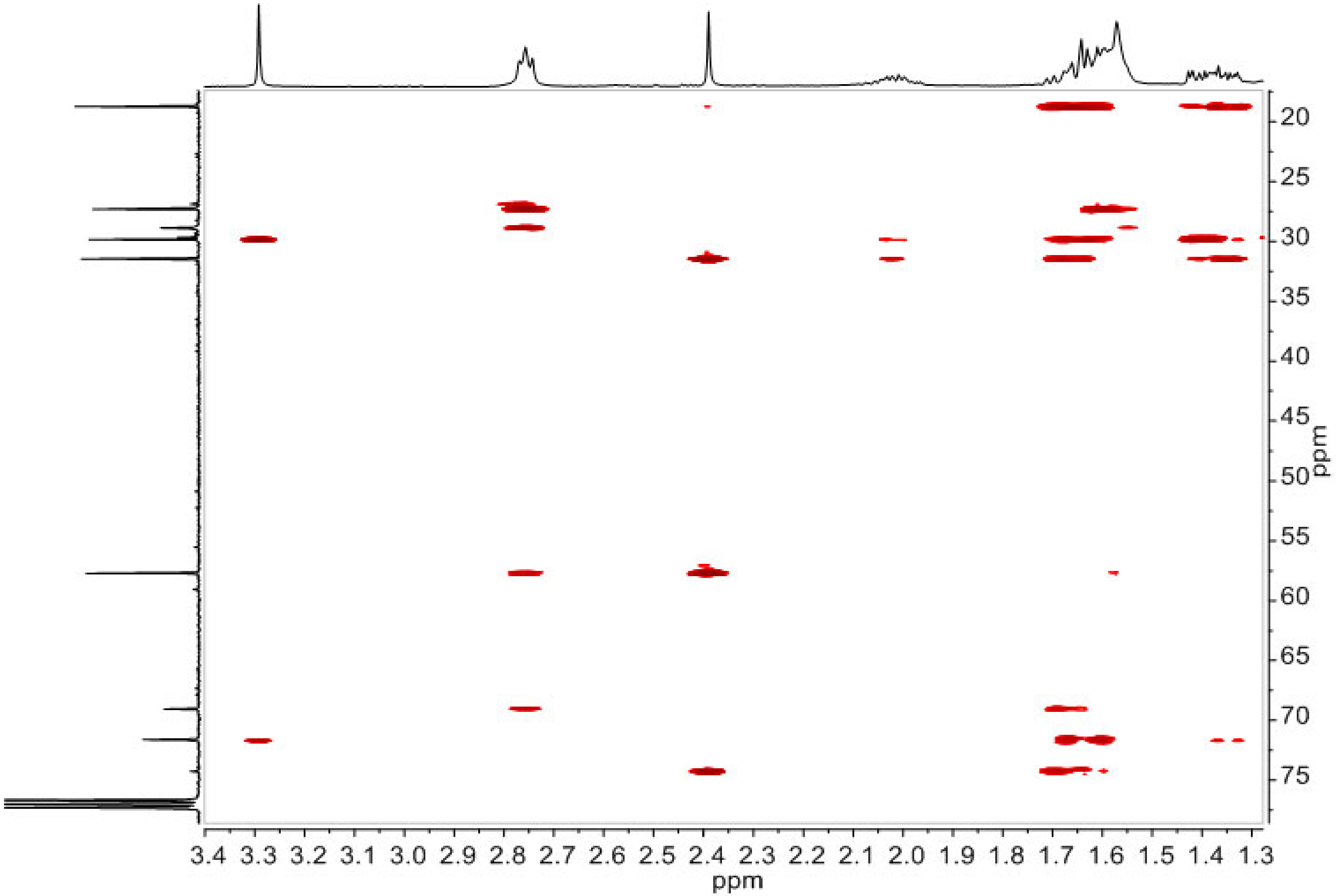

^{1}H NMR spectrum (400 MHz, $CDCl_3$) of compound **5d**

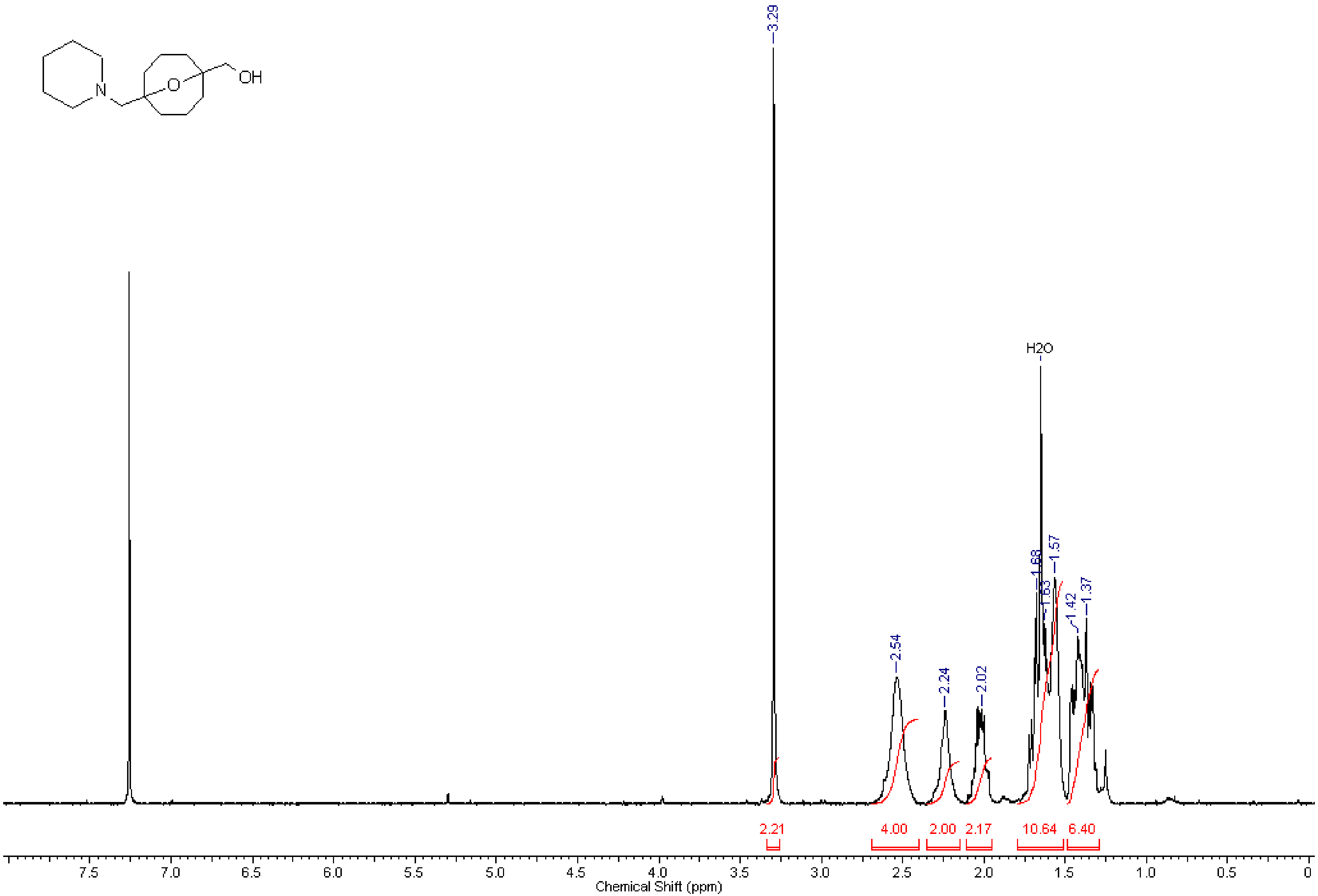

^{13}C NMR spectrum (101 MHz, $CDCl_3$) of compound **5d**

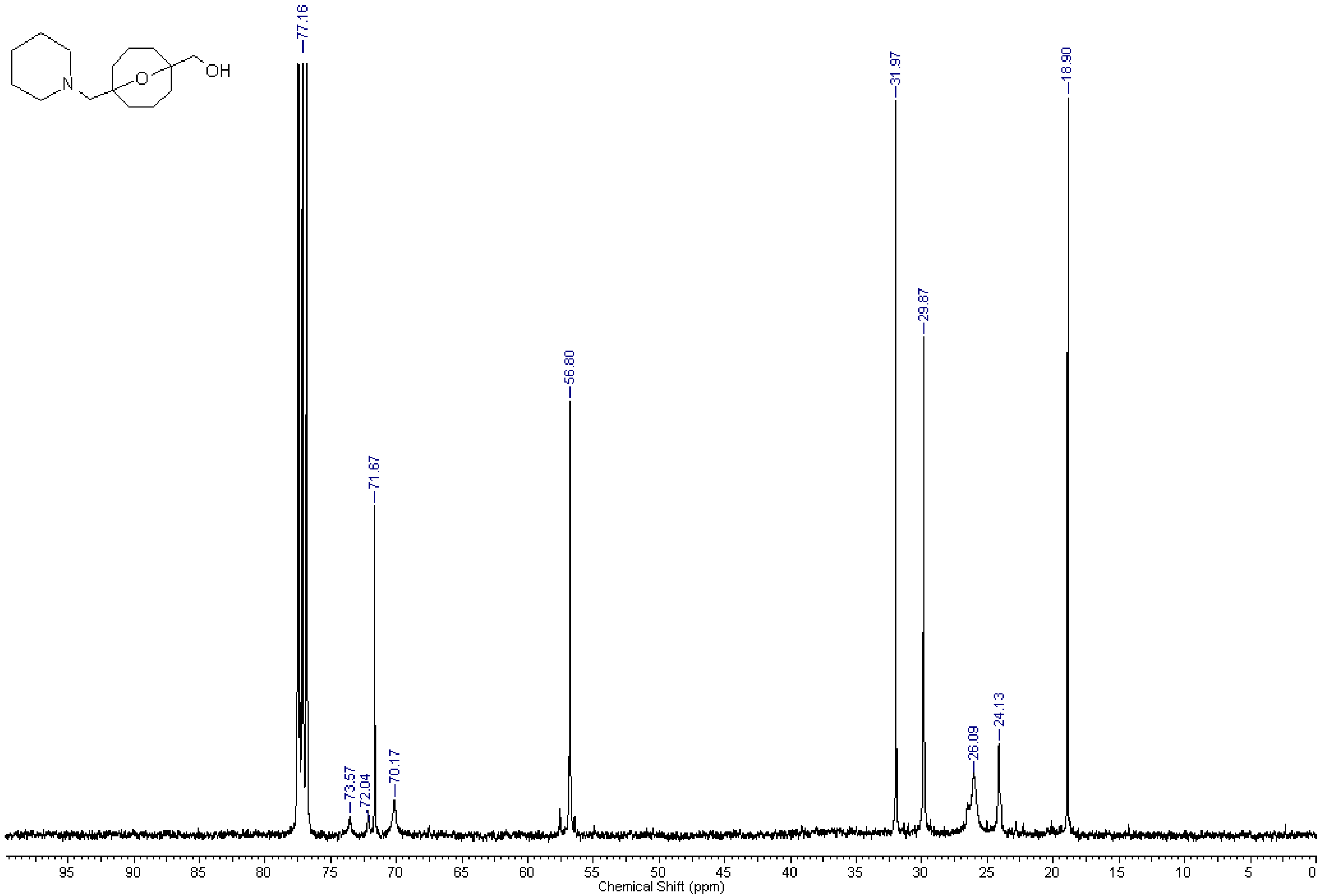

HSQC NMR spectrum of compound **5d**

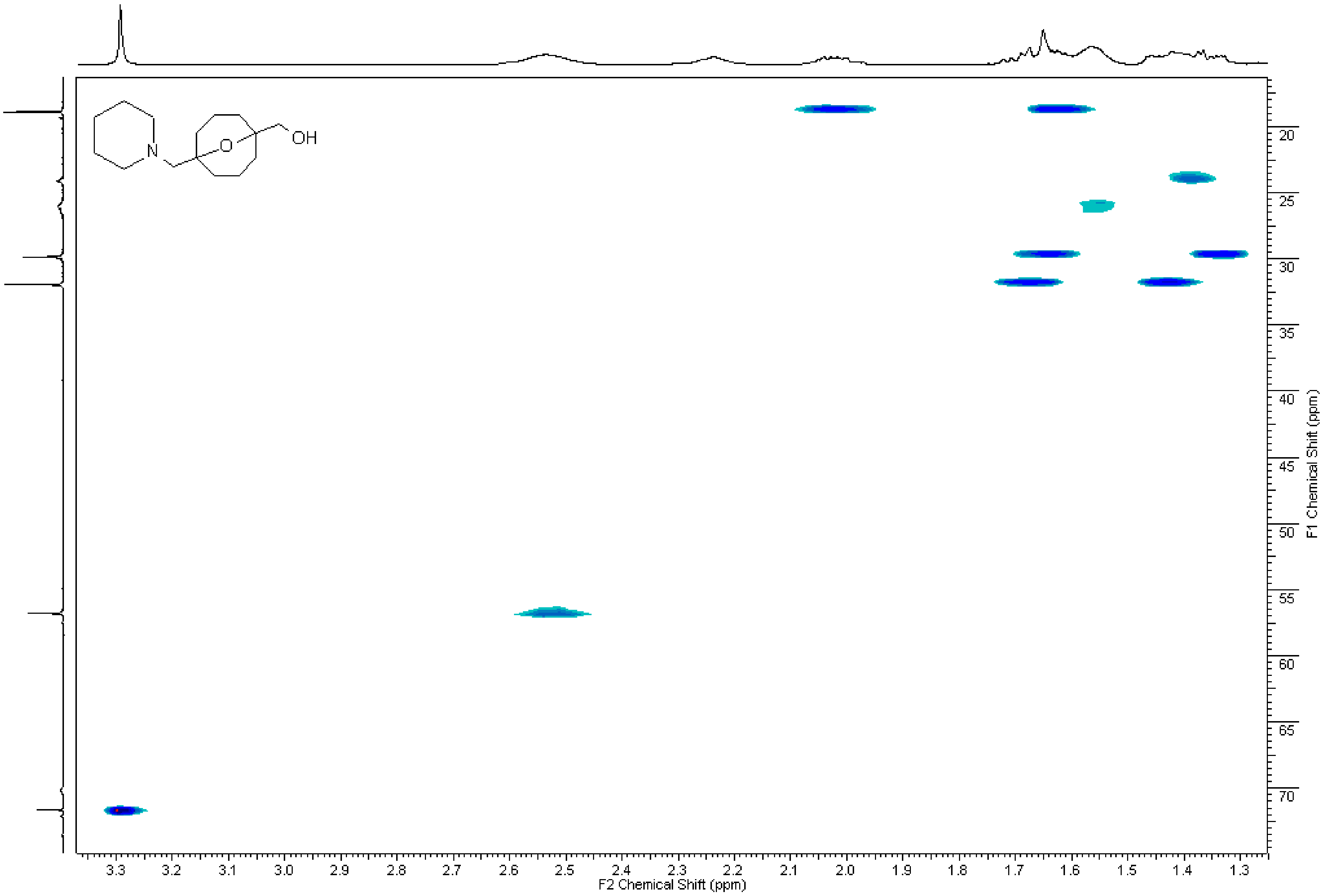

^{1}H NMR spectrum (400 MHz, $CDCl_3$) of compound **5e**

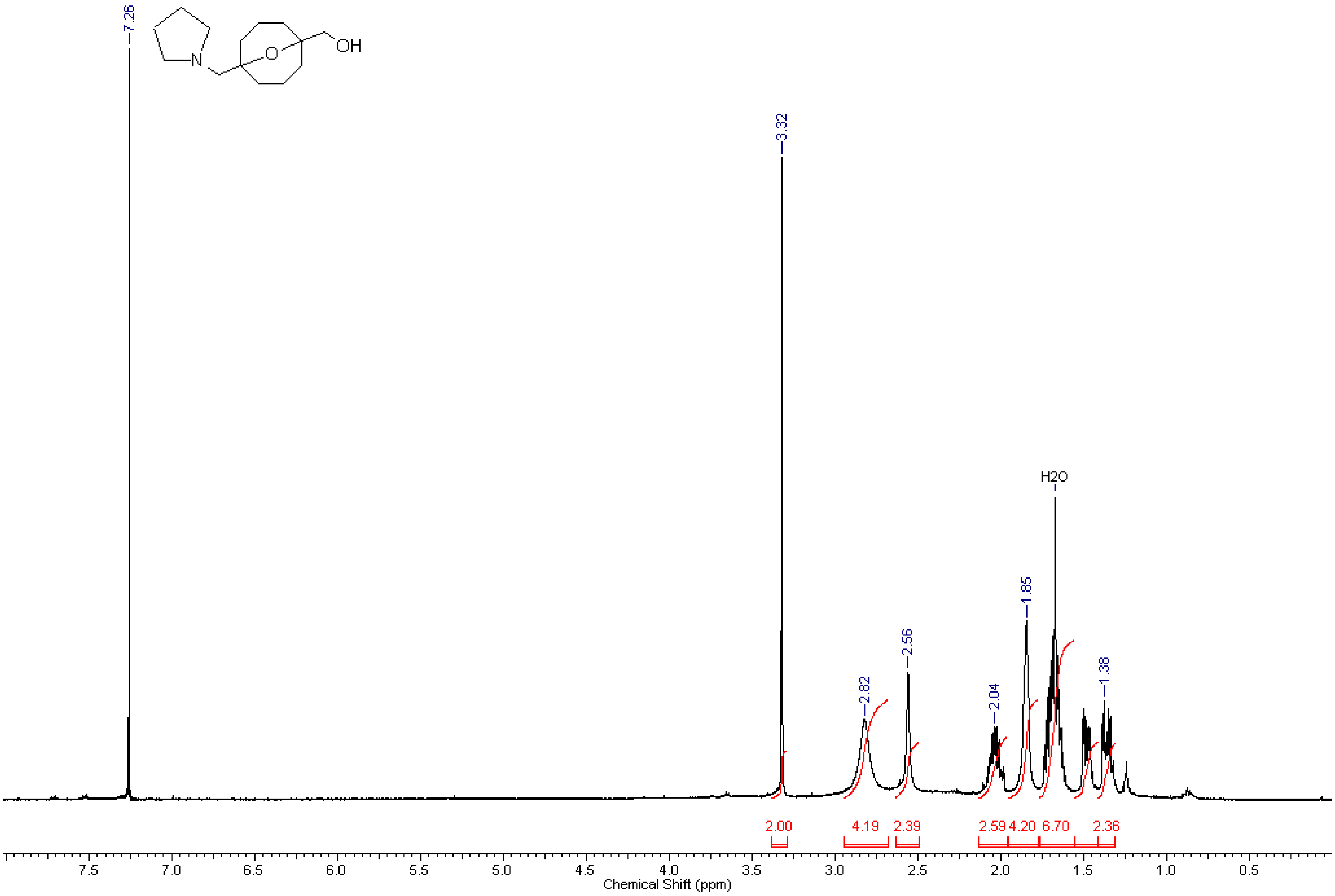

^{13}C NMR spectrum (101 MHz, $CDCl_3$) of compound **5e**

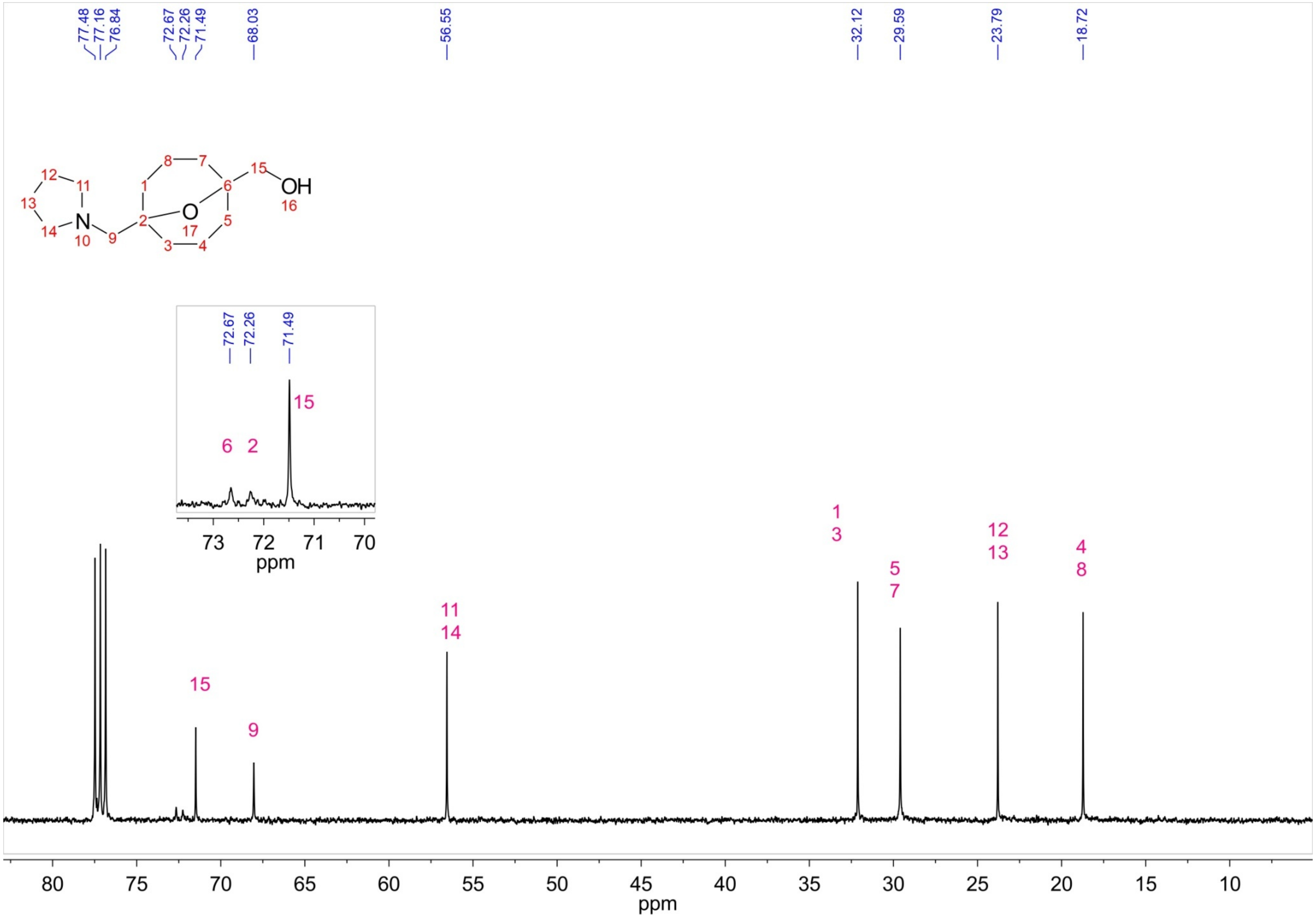

HSQC NMR spectrum of compound **5e**

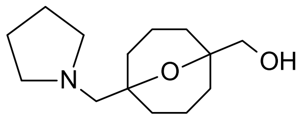

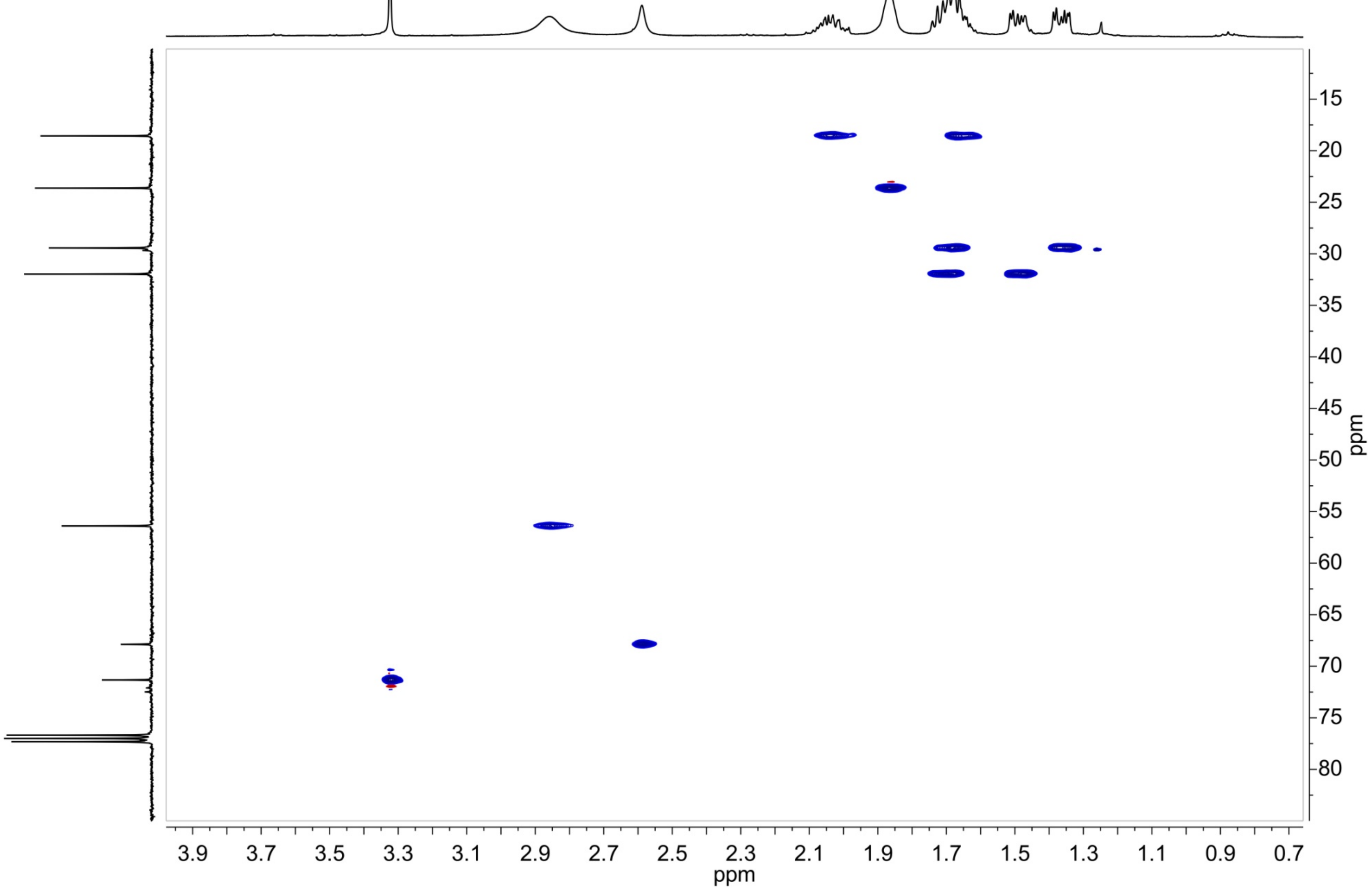

HMBC NMR spectrum of compound **5e**

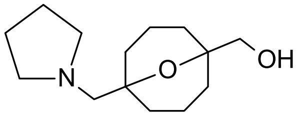

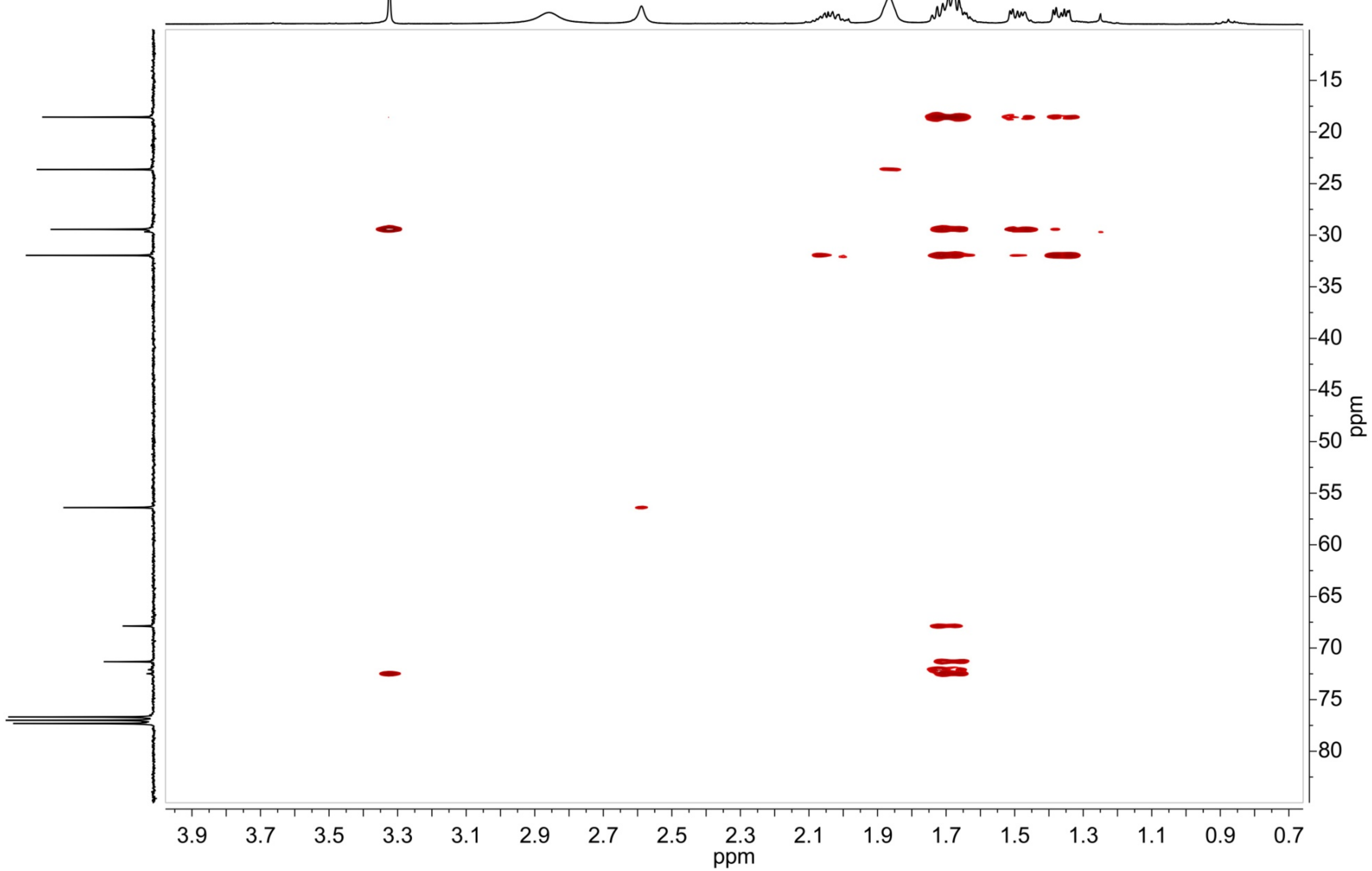

^{1}H NMR spectrum (400 MHz, $CDCl_3$) of compound **5f**

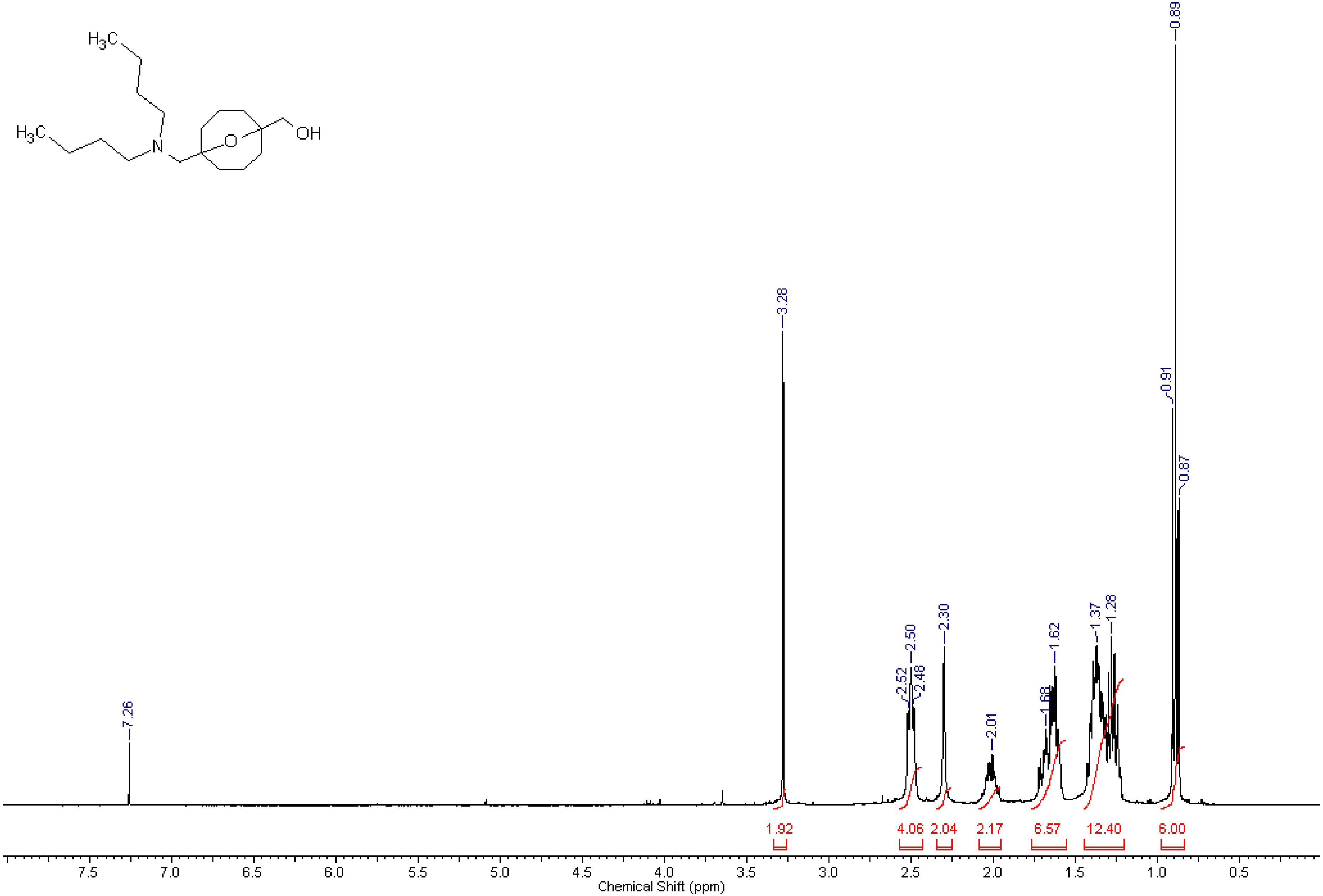

^{13}C NMR spectrum (101 MHz, $CDCl_3$) of compound **5f**

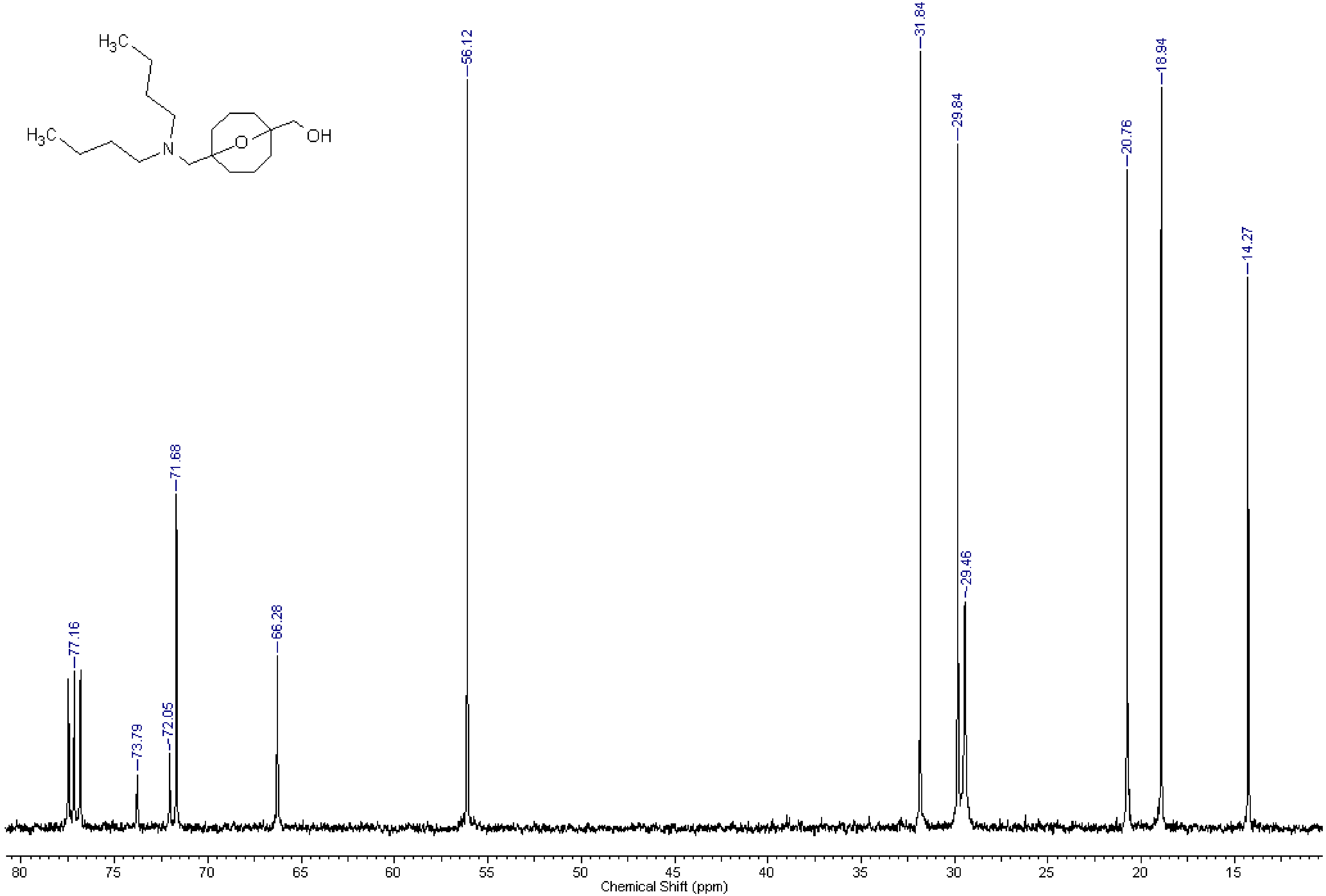

HSQC NMR spectrum ($CDCl_3$) of compound **5f**

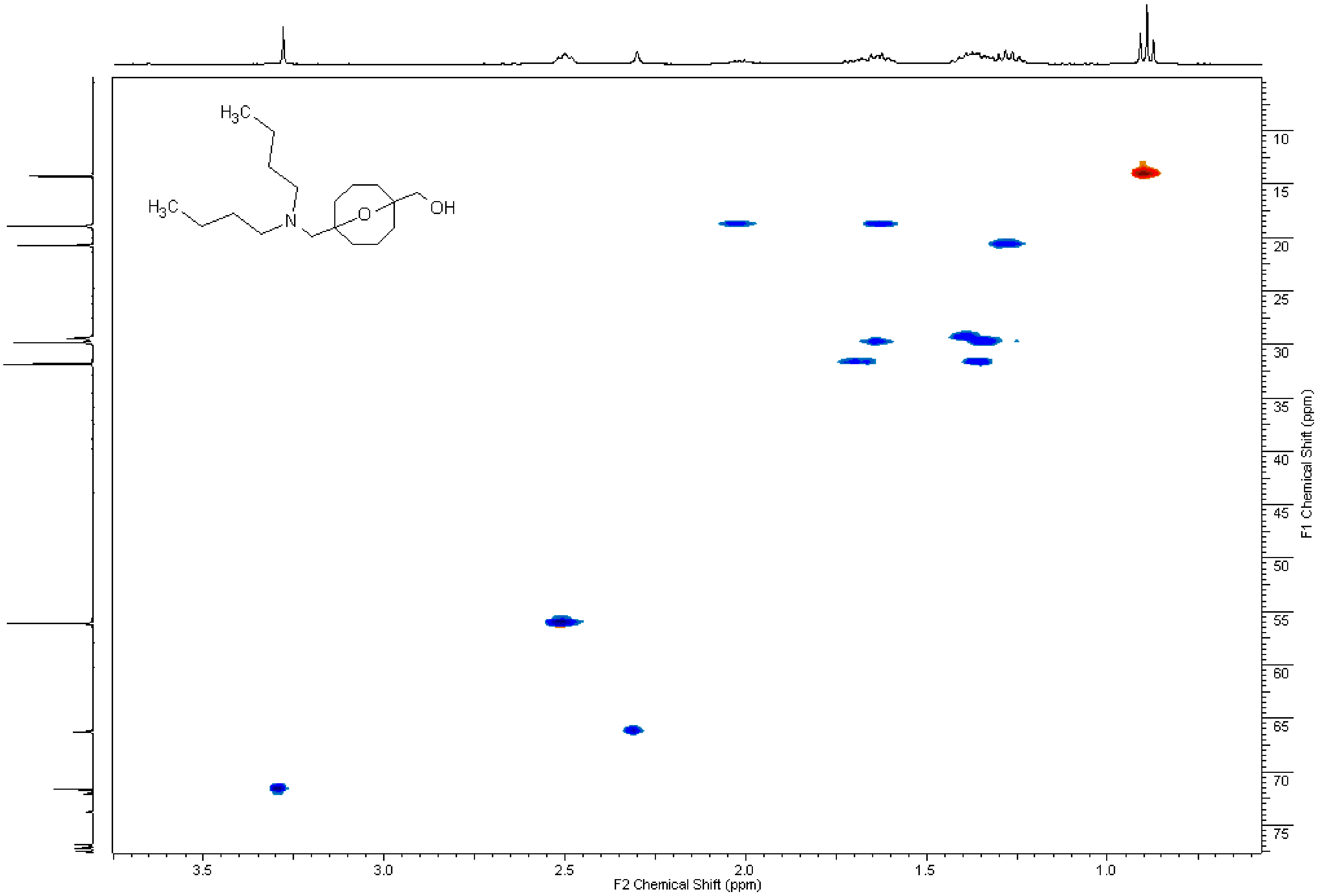

HMBC NMR spectrum ($CDCl_3$) of compound **5f**

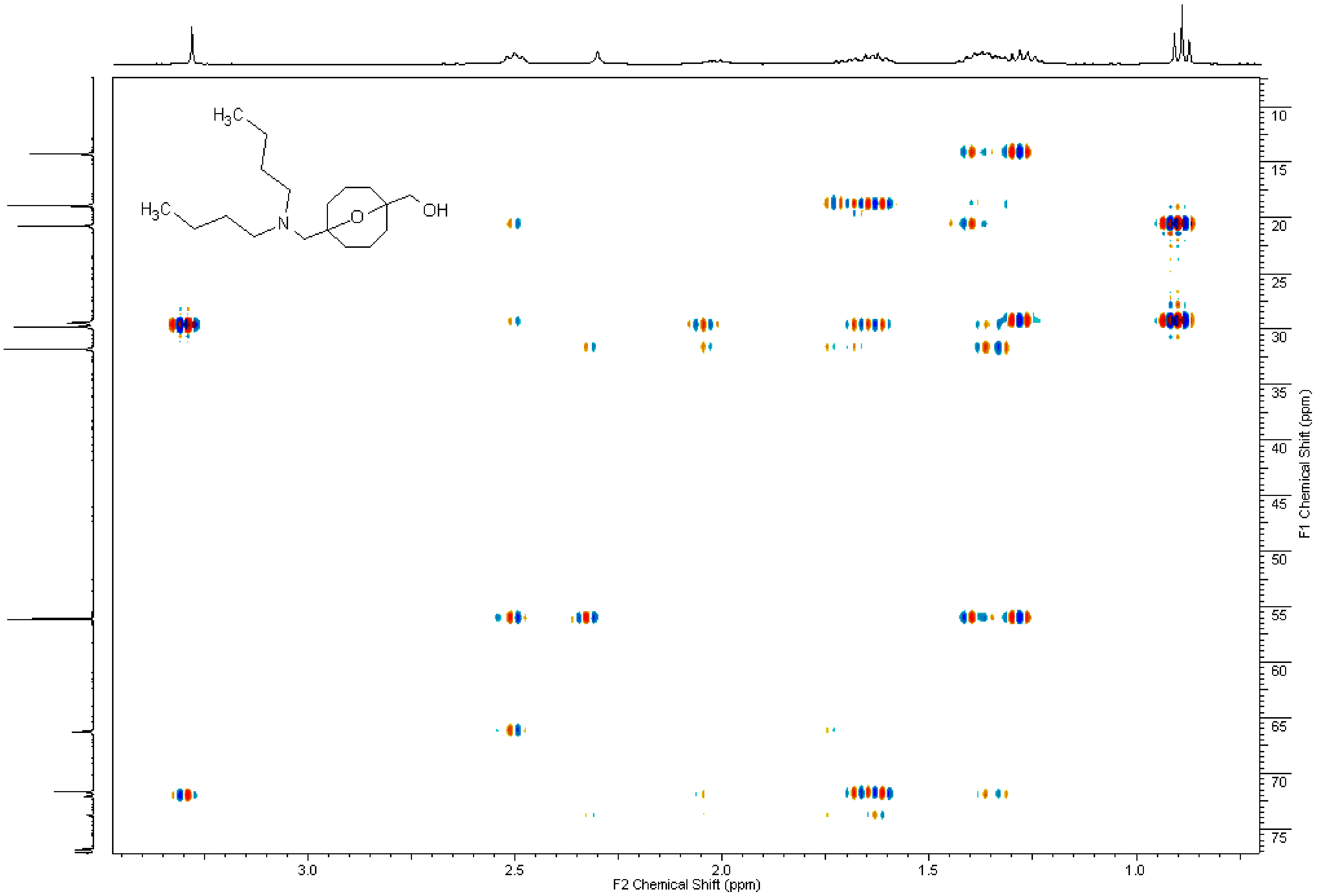

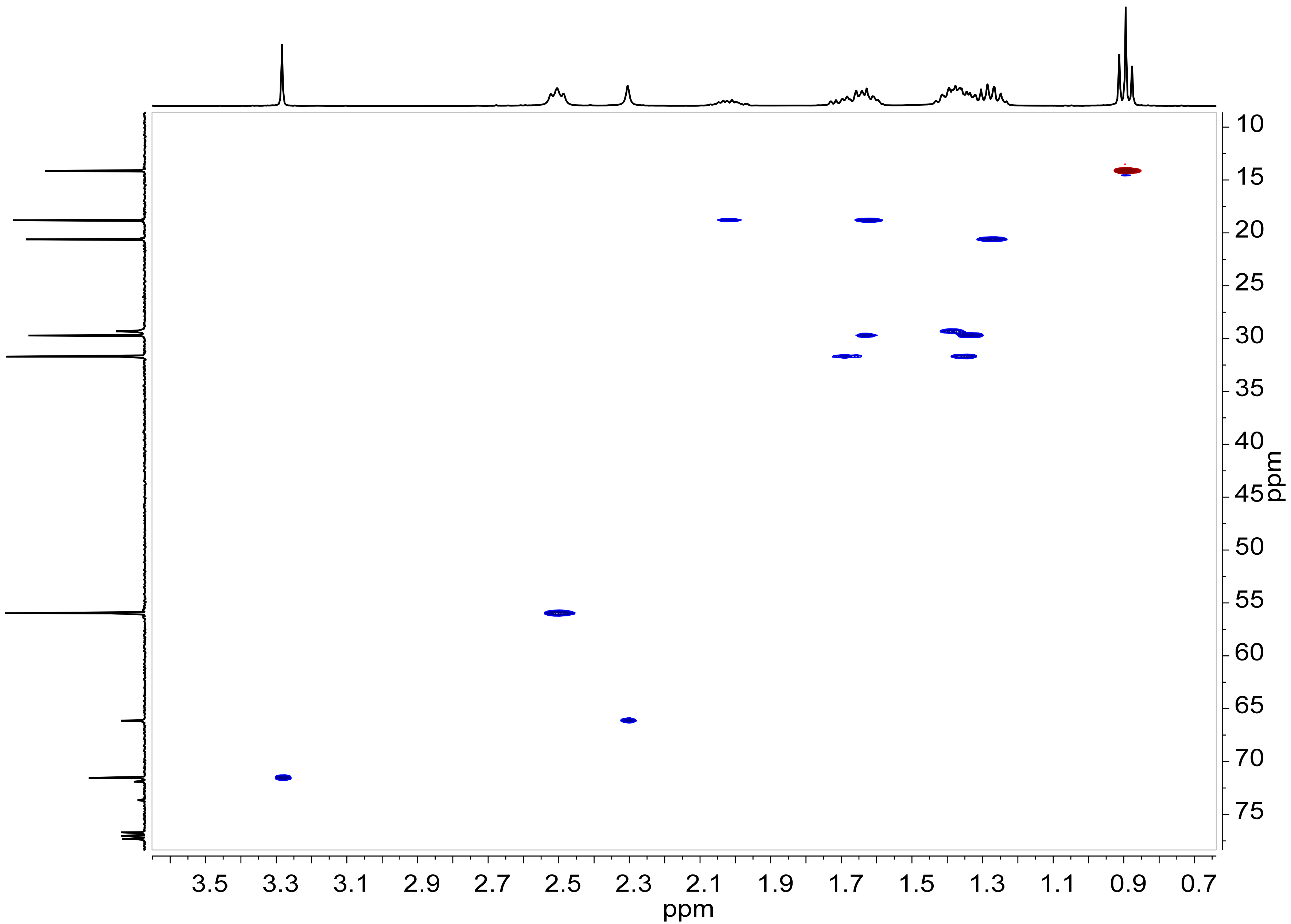
10
15
20
25
30
35
40
45
50
55
60
65
70
75
ppm
3.5
3.3
3.1
2.9
2.7
2.5
2.3
2.1
1.9
1.7
1.5
1.3
1.1
0.9
0.7
ppm

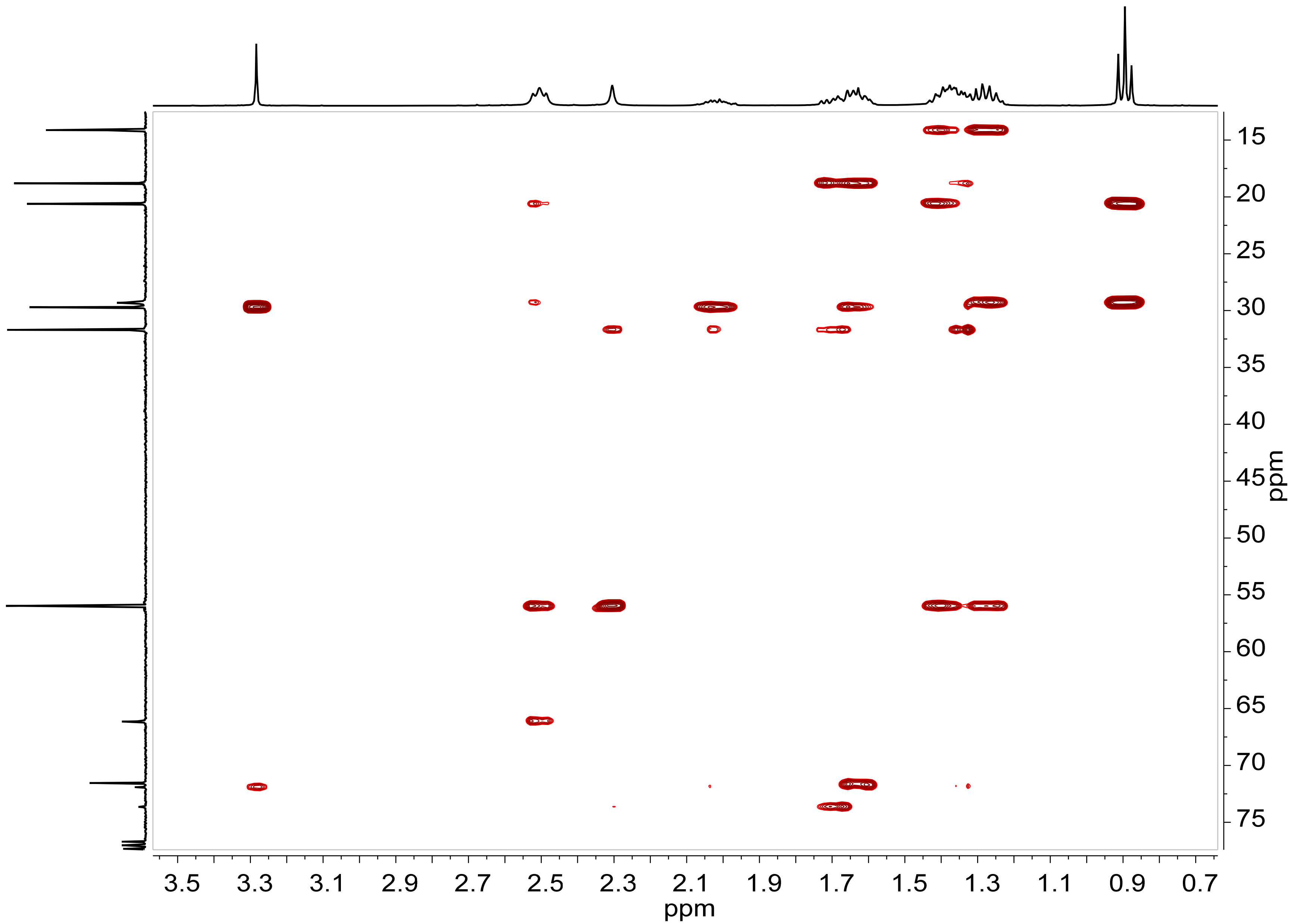

15
20
25
30
35
40
45
50
55
60
65
70
75
ppm
3.5
3.3
3.1
2.9
2.7
2.5
2.3
2.1
1.9
1.7
1.5
1.3
1.1
0.9
0.7
ppm

^{1}H NMR spectrum (400 MHz, $CDCl_3$) of compound **5g**

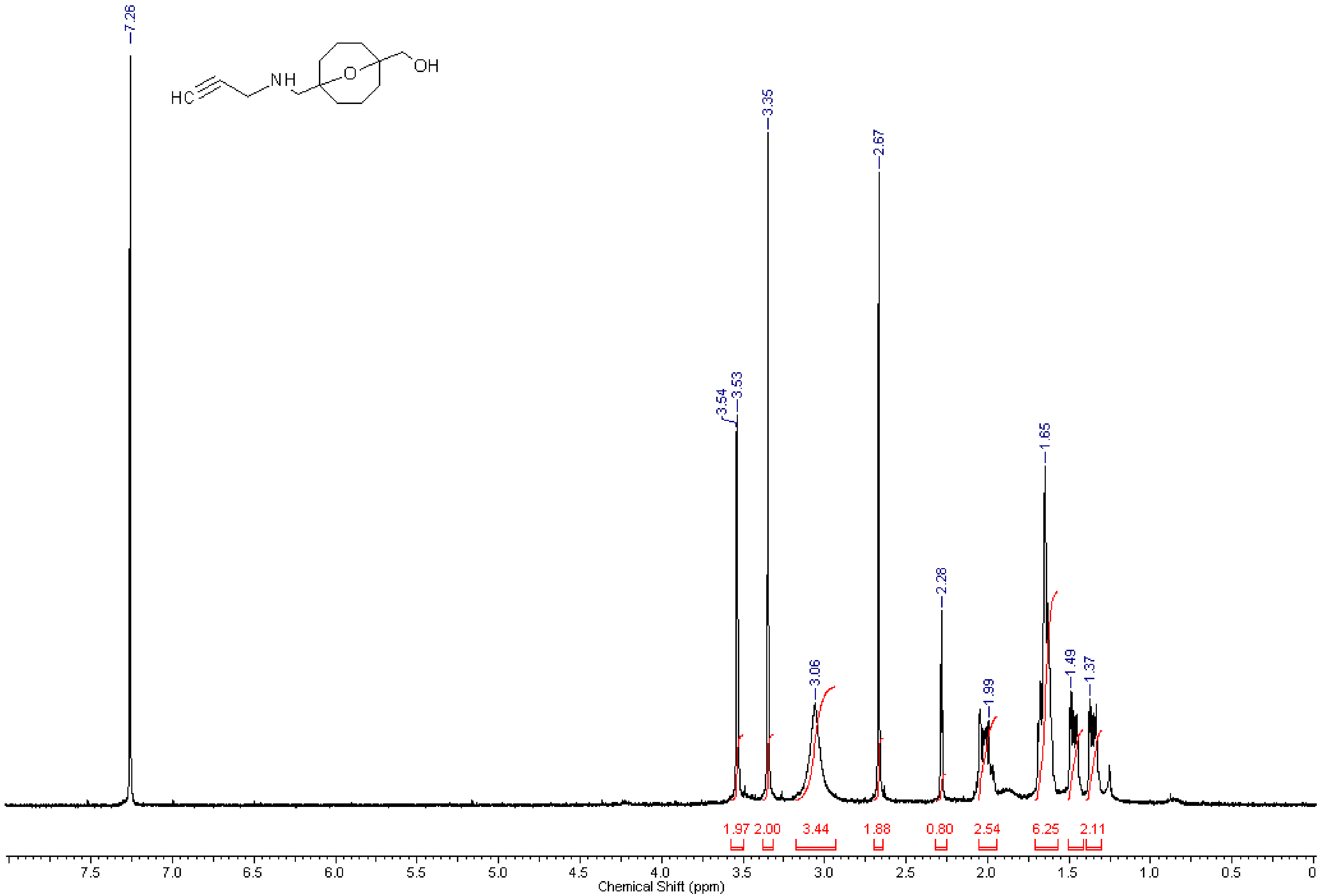

^{13}C NMR spectrum (101 MHz, $CDCl_3$) of compound **5g**

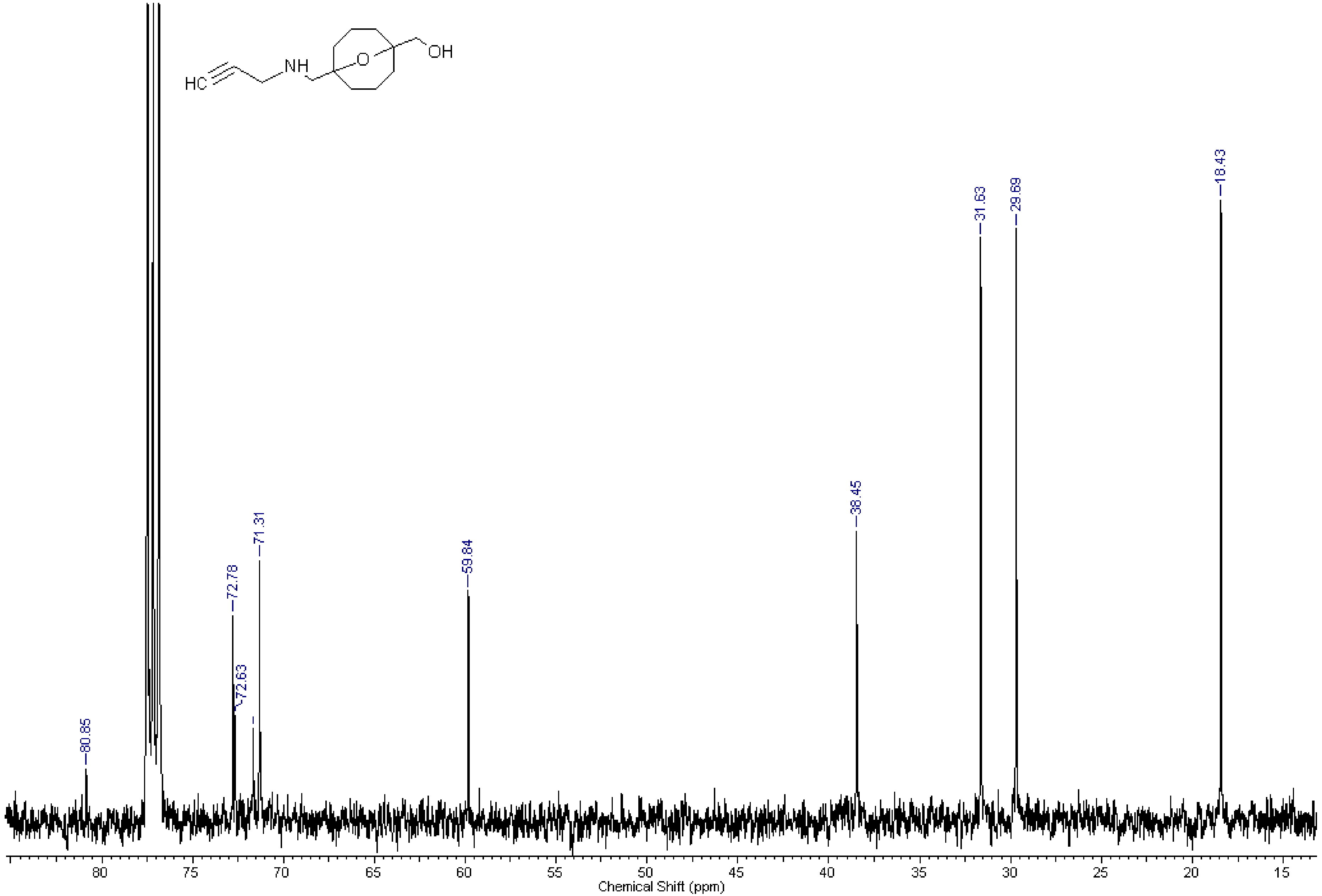

HSQC NMR spectrum of compound **5g**

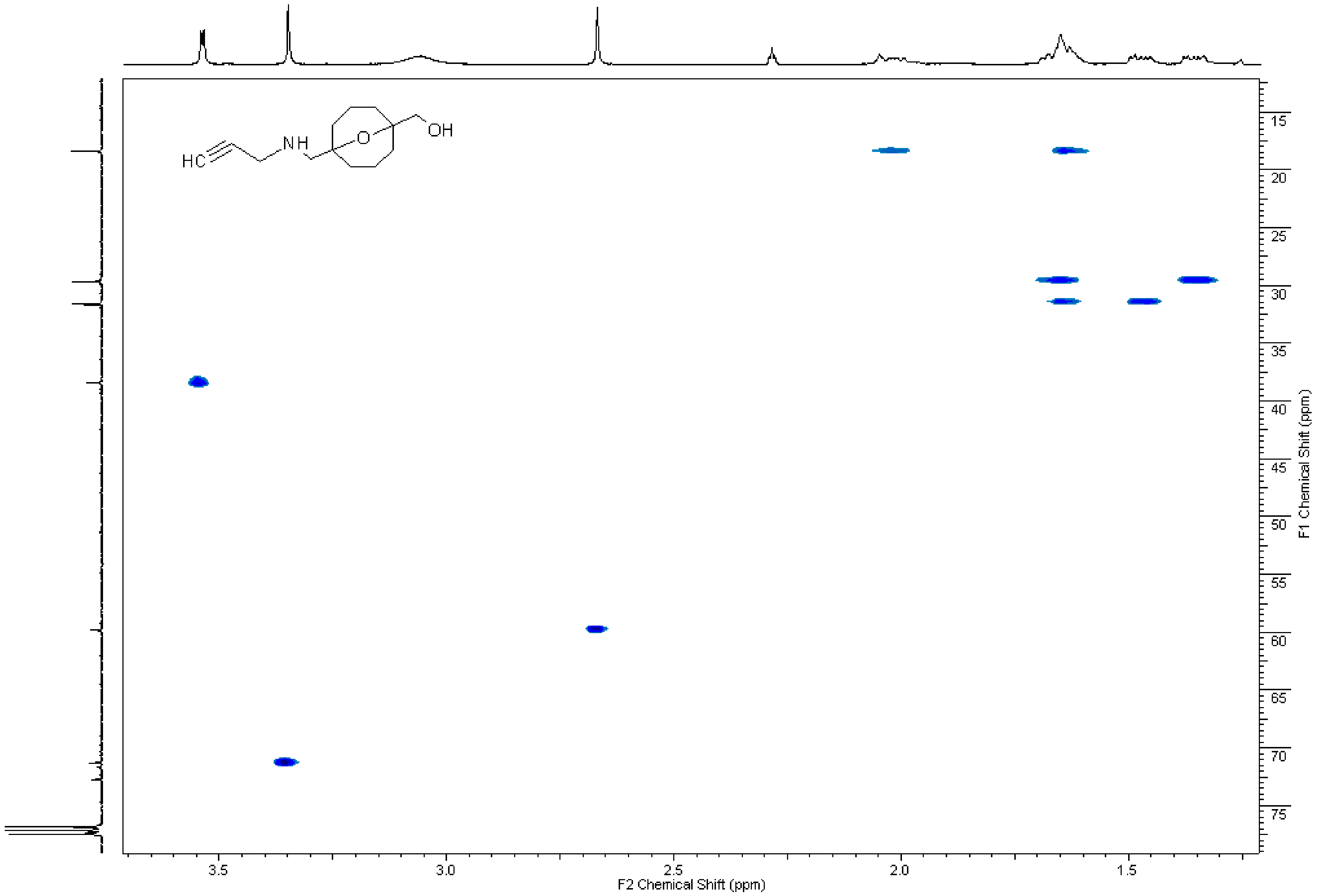

HMBC NMR spectrum of compound **5g**

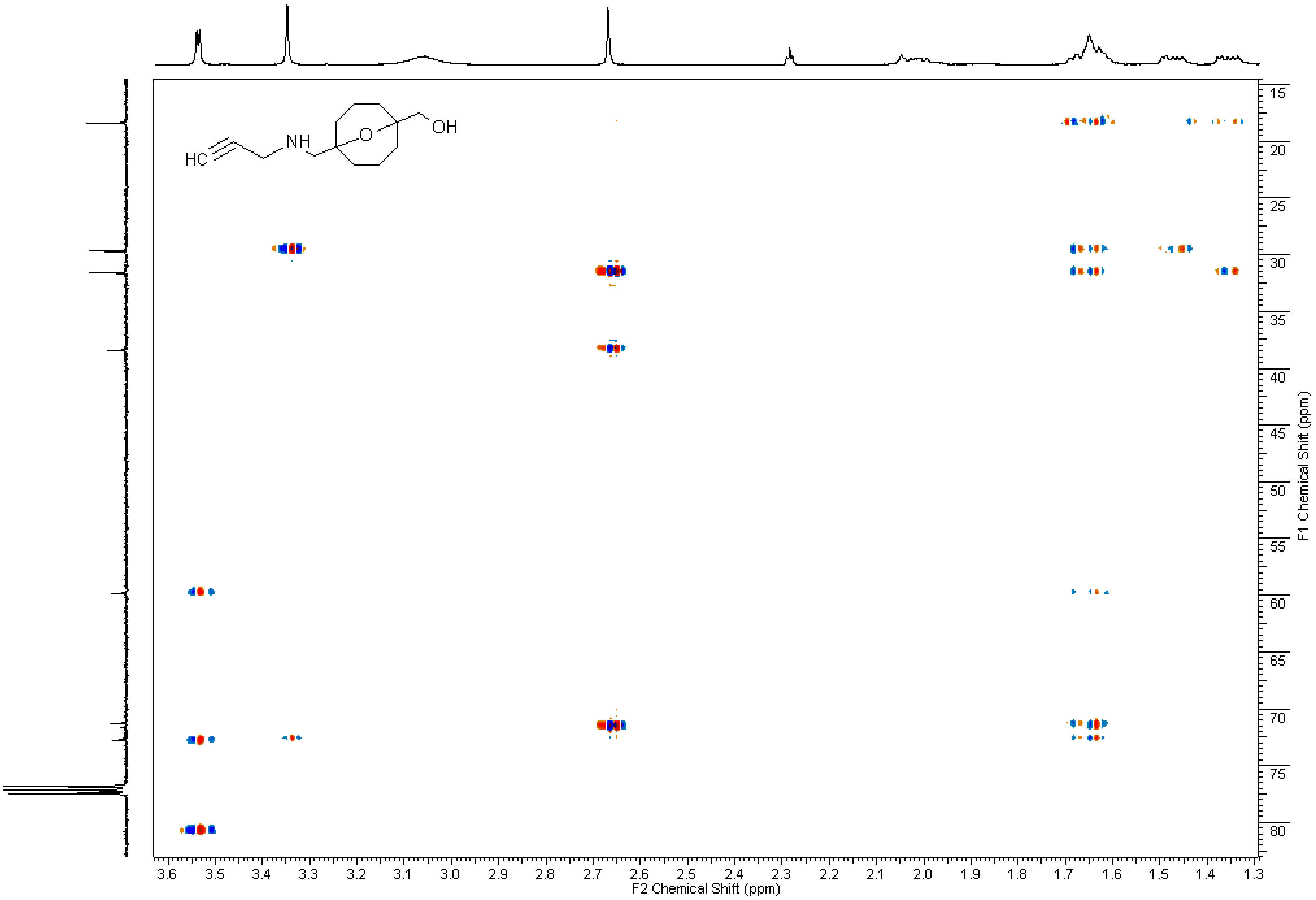

^{1}H NMR spectrum (400 MHz, $CDCl_3$) of compound **5h**

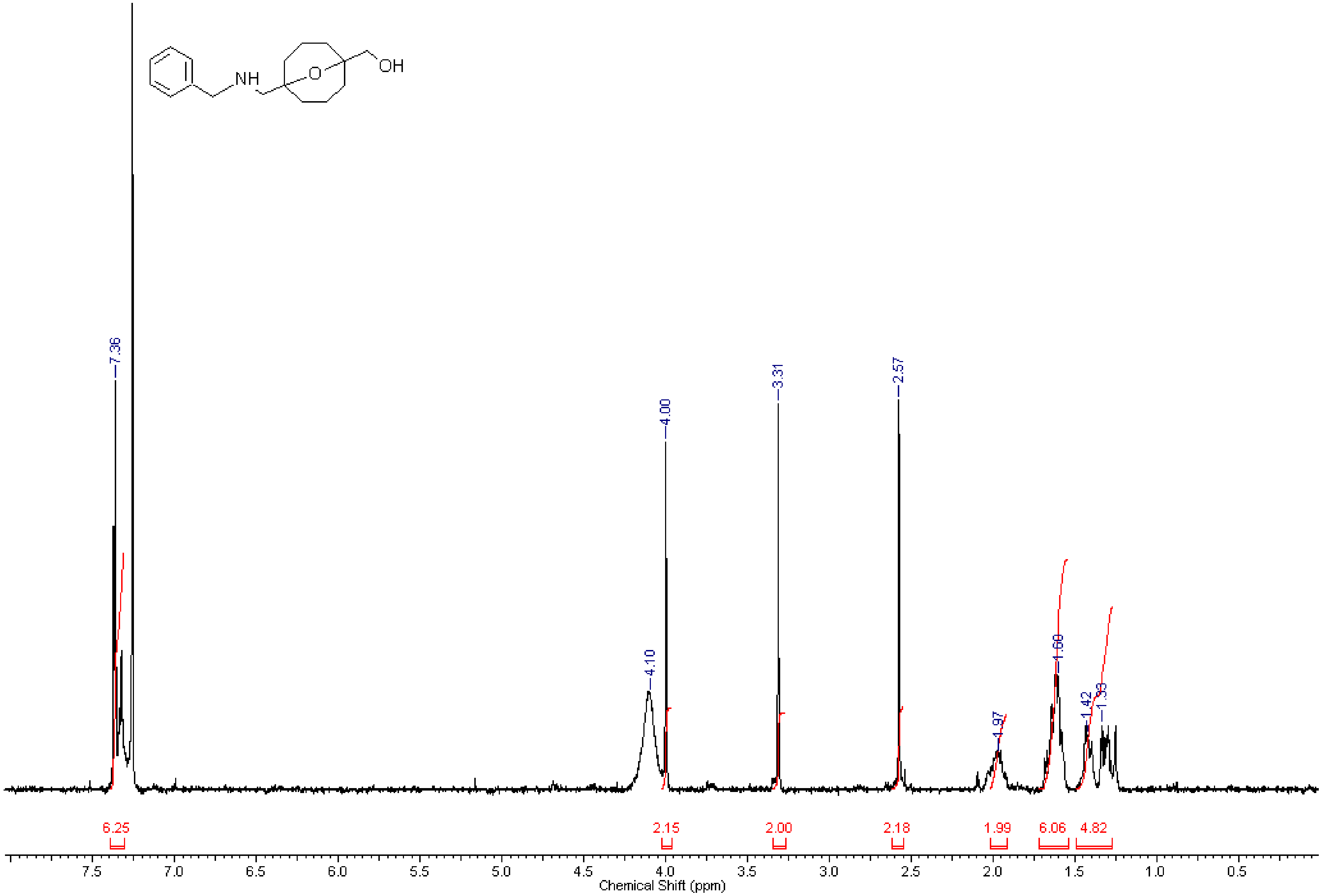

^{13}C NMR spectrum (101 MHz, $CDCl_3$) of compound **5h**

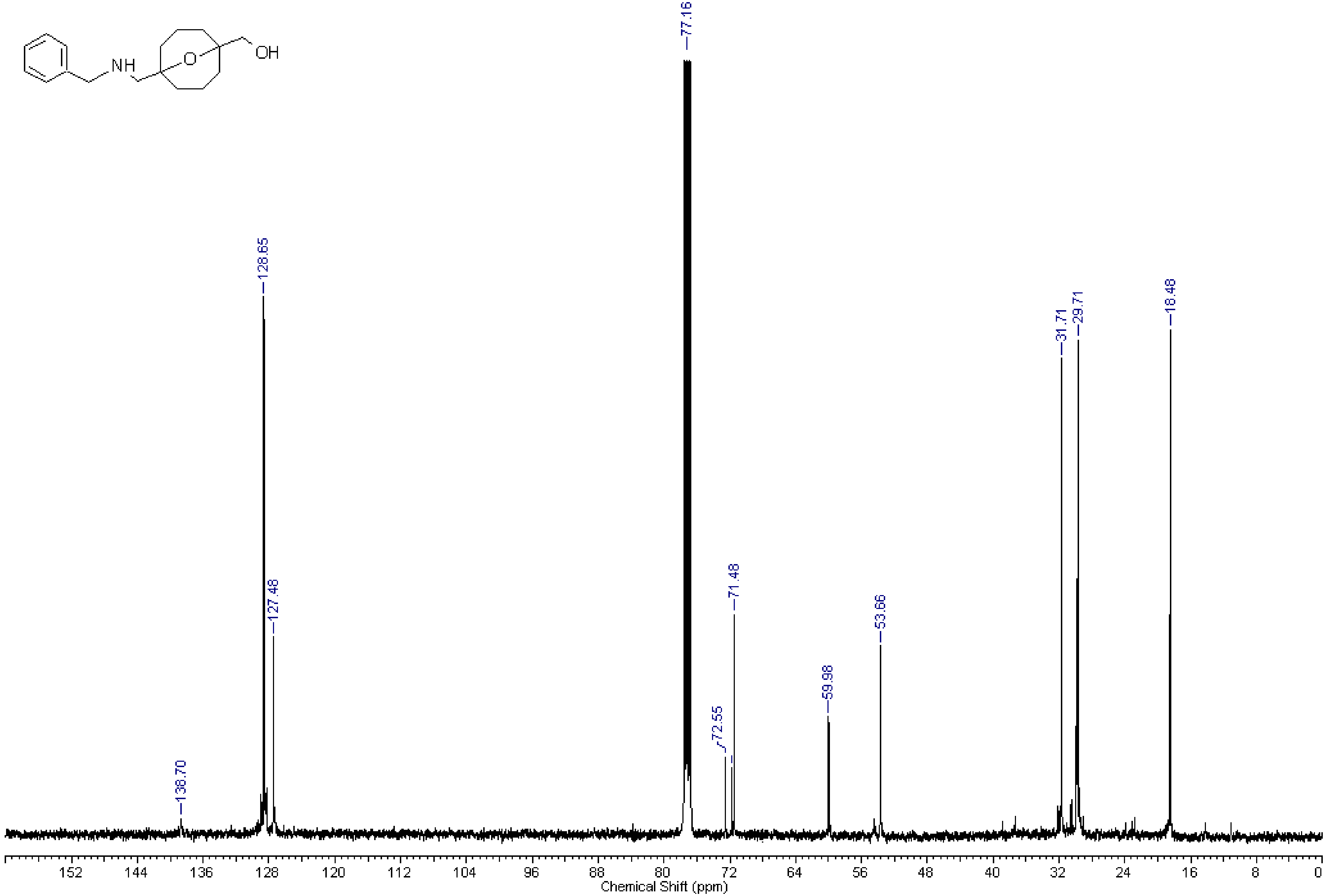

^{1}H NMR spectrum (400 MHz, $CDCl_3$) of compound **5i**

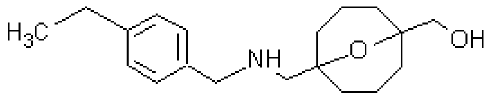

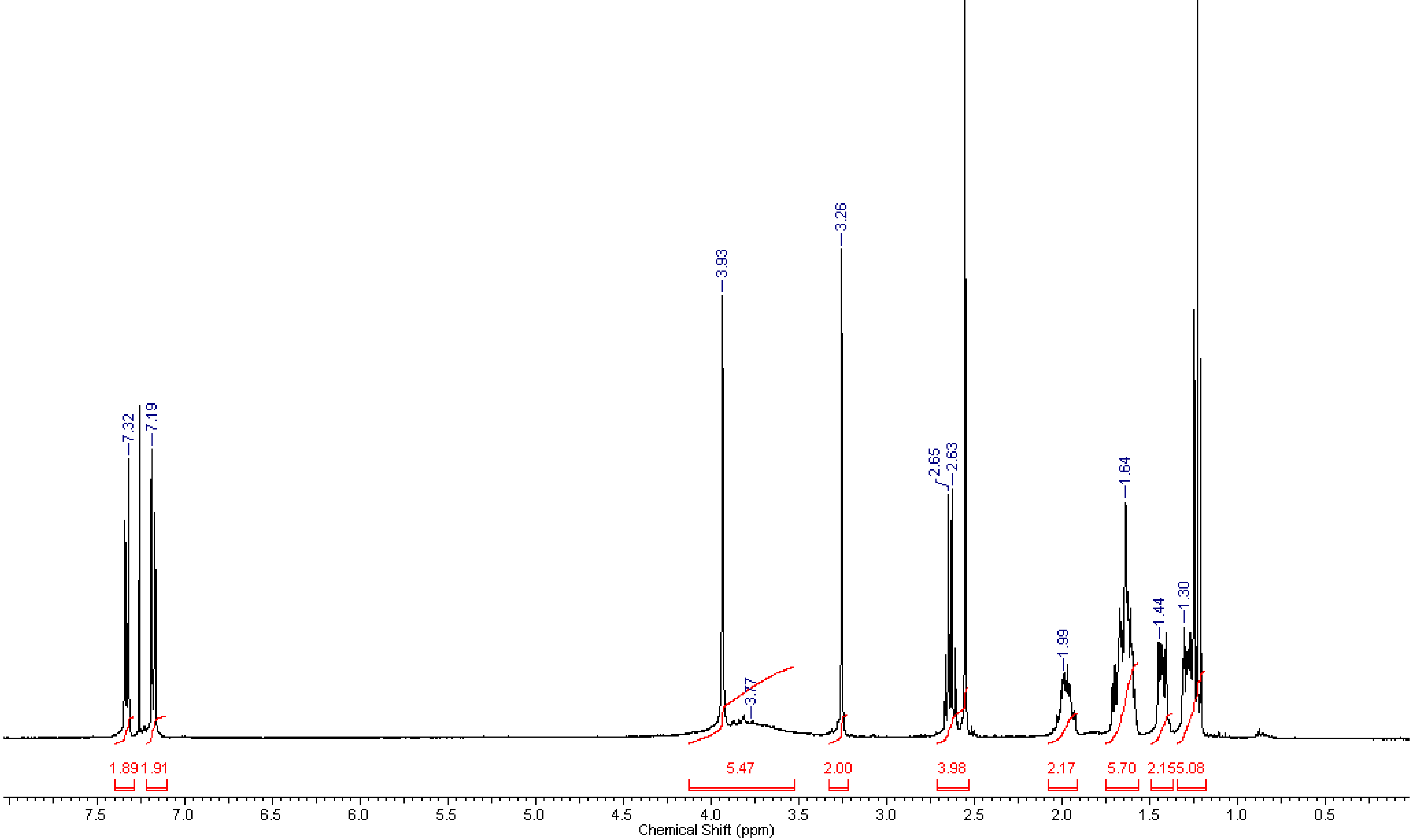

^{13}C NMR spectrum (101 MHz, $CDCl_3$) of compound **5i**

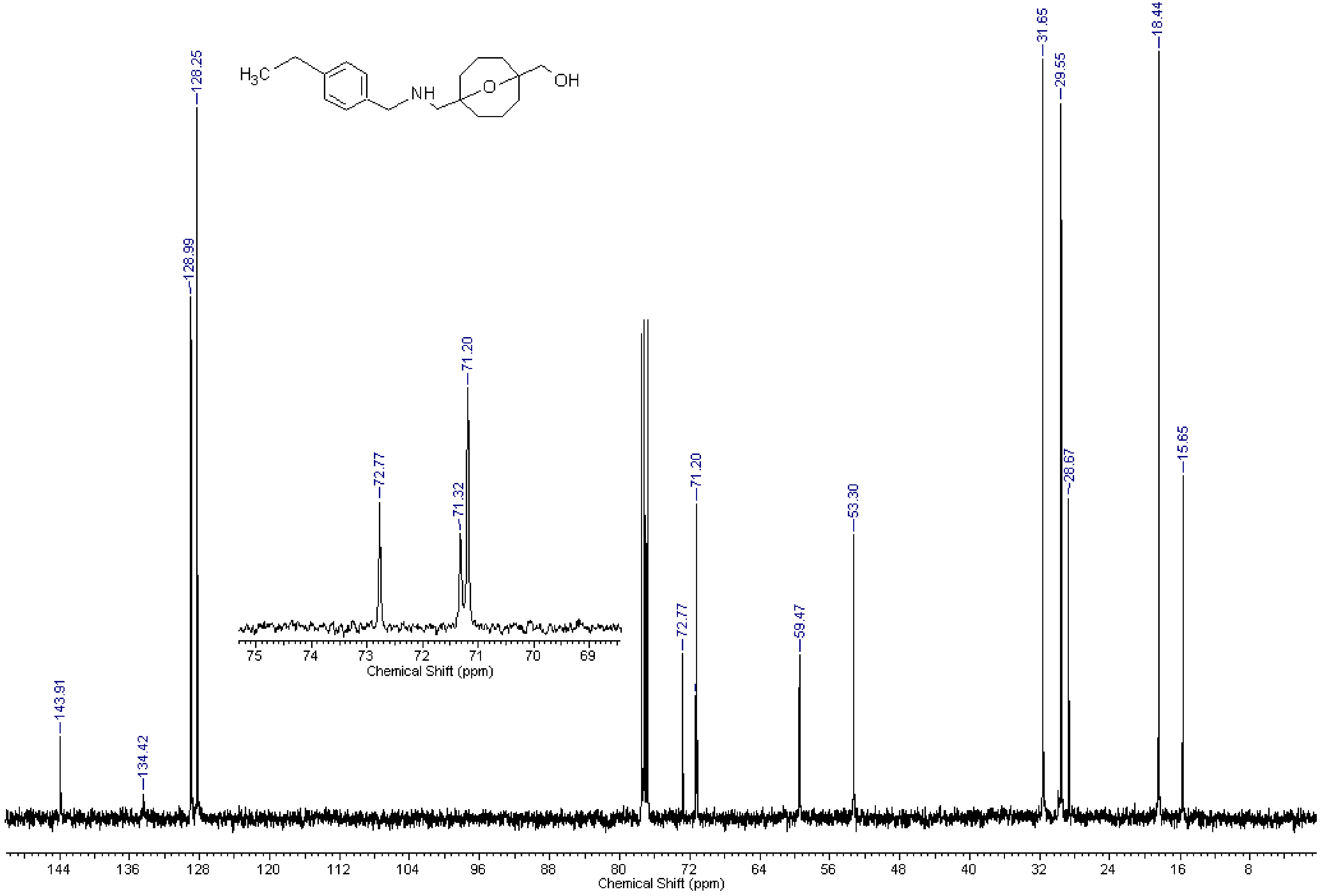

HSQC NMR spectrum of compound **5i**

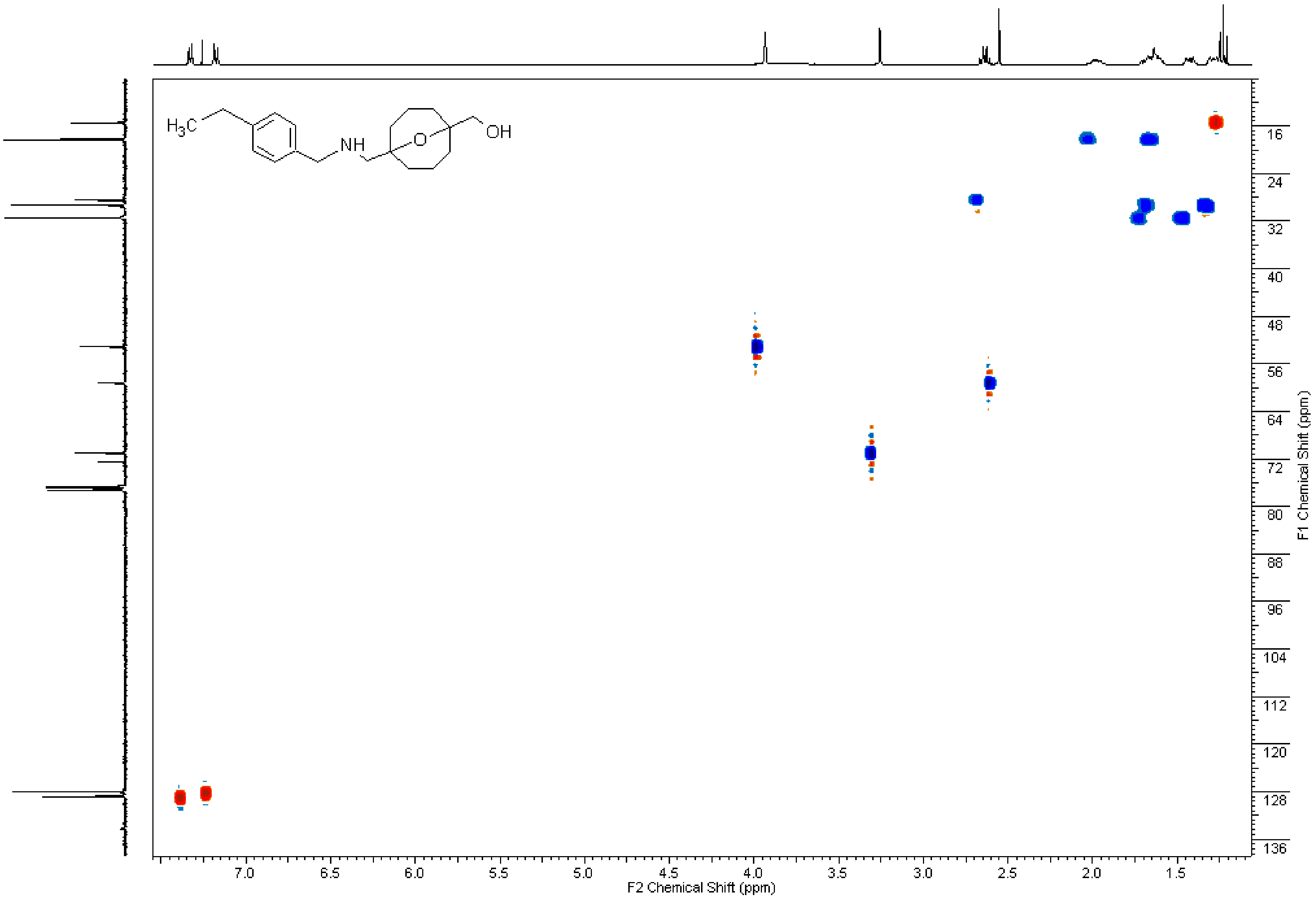

HMBC NMR spectrum of compound **5i**

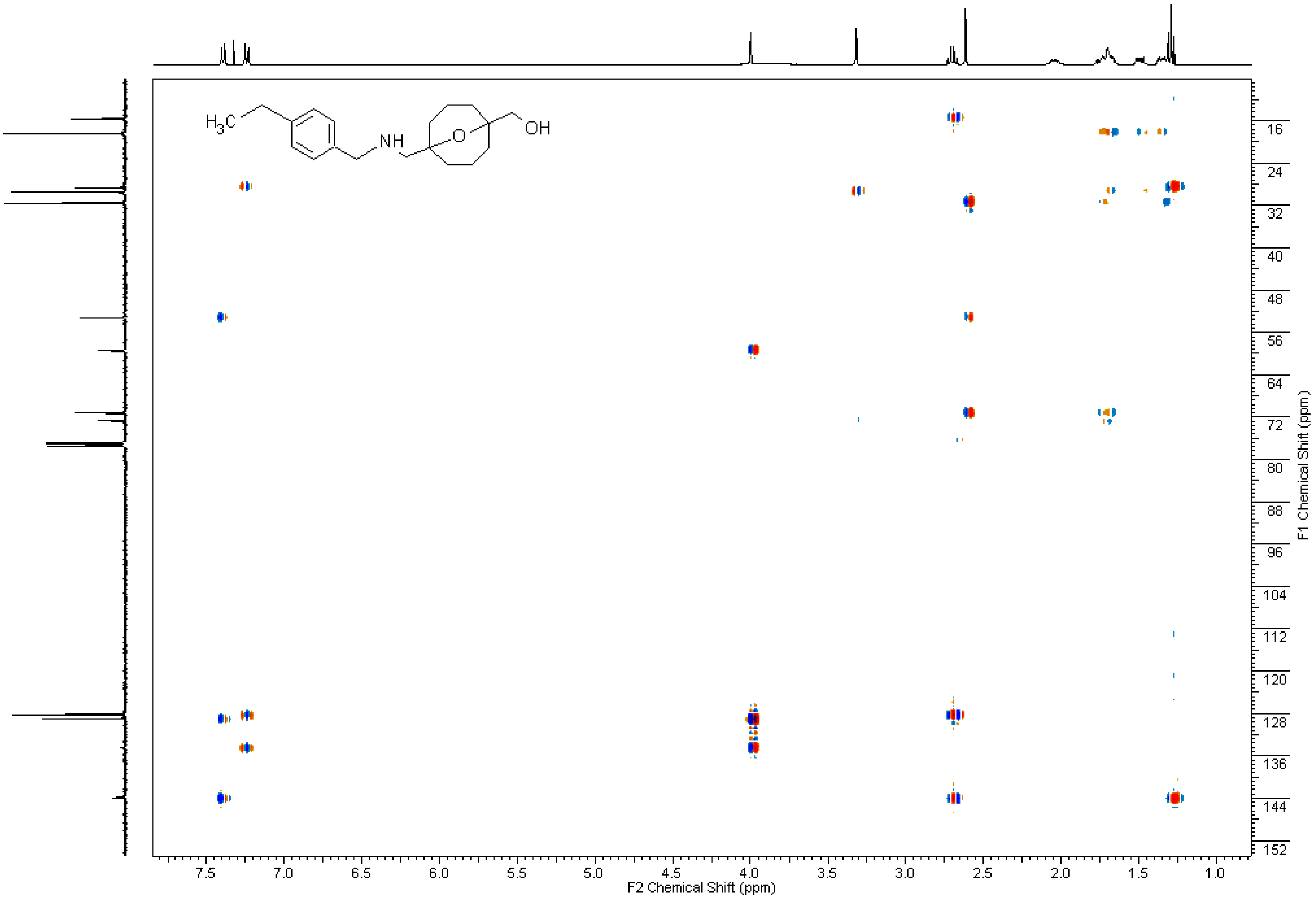

^{1}H NMR spectrum (400 MHz, $CDCl_3$) of compound **5j**

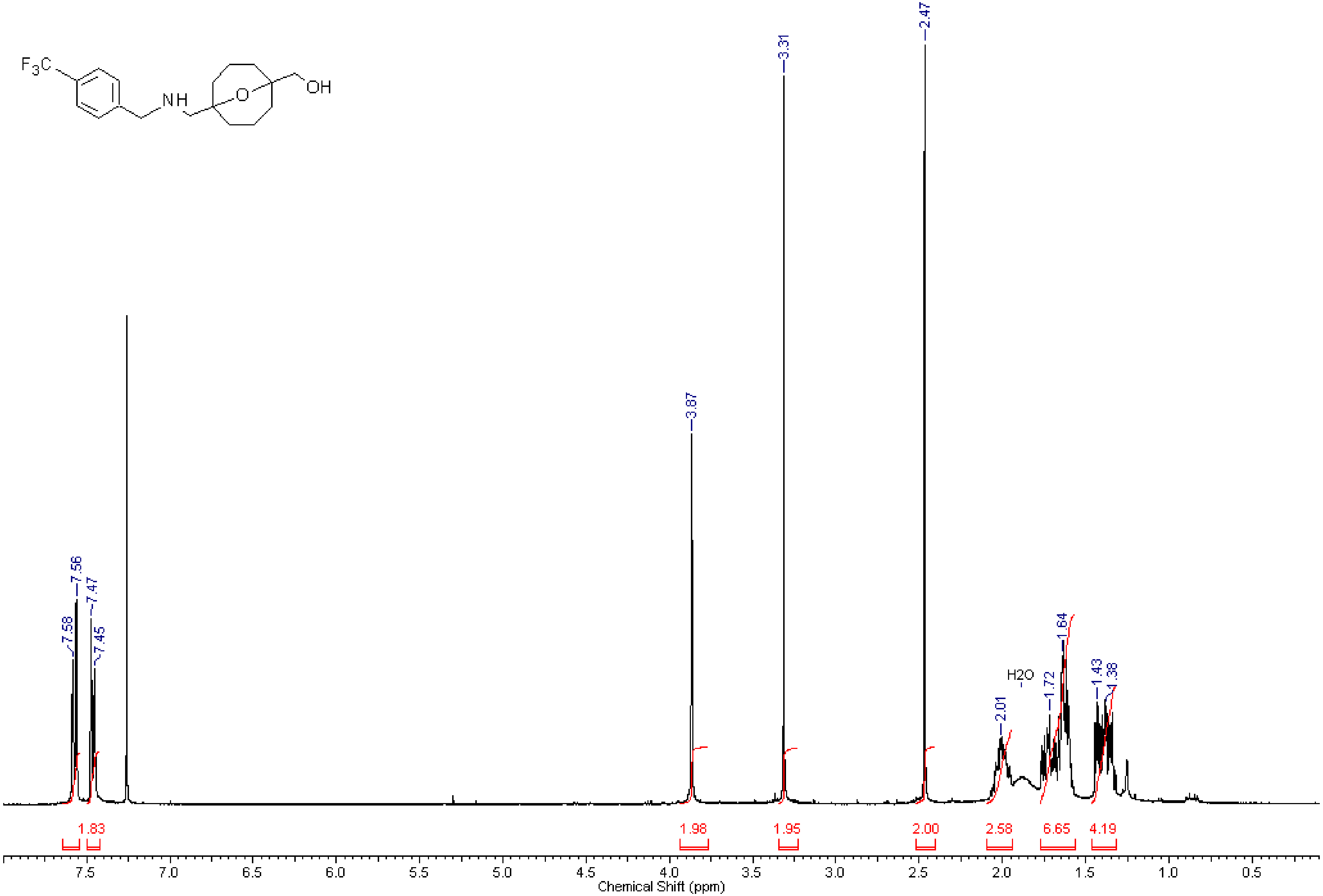

^{13}C NMR spectrum (101 MHz, $CDCl_3$) of compound **5j**

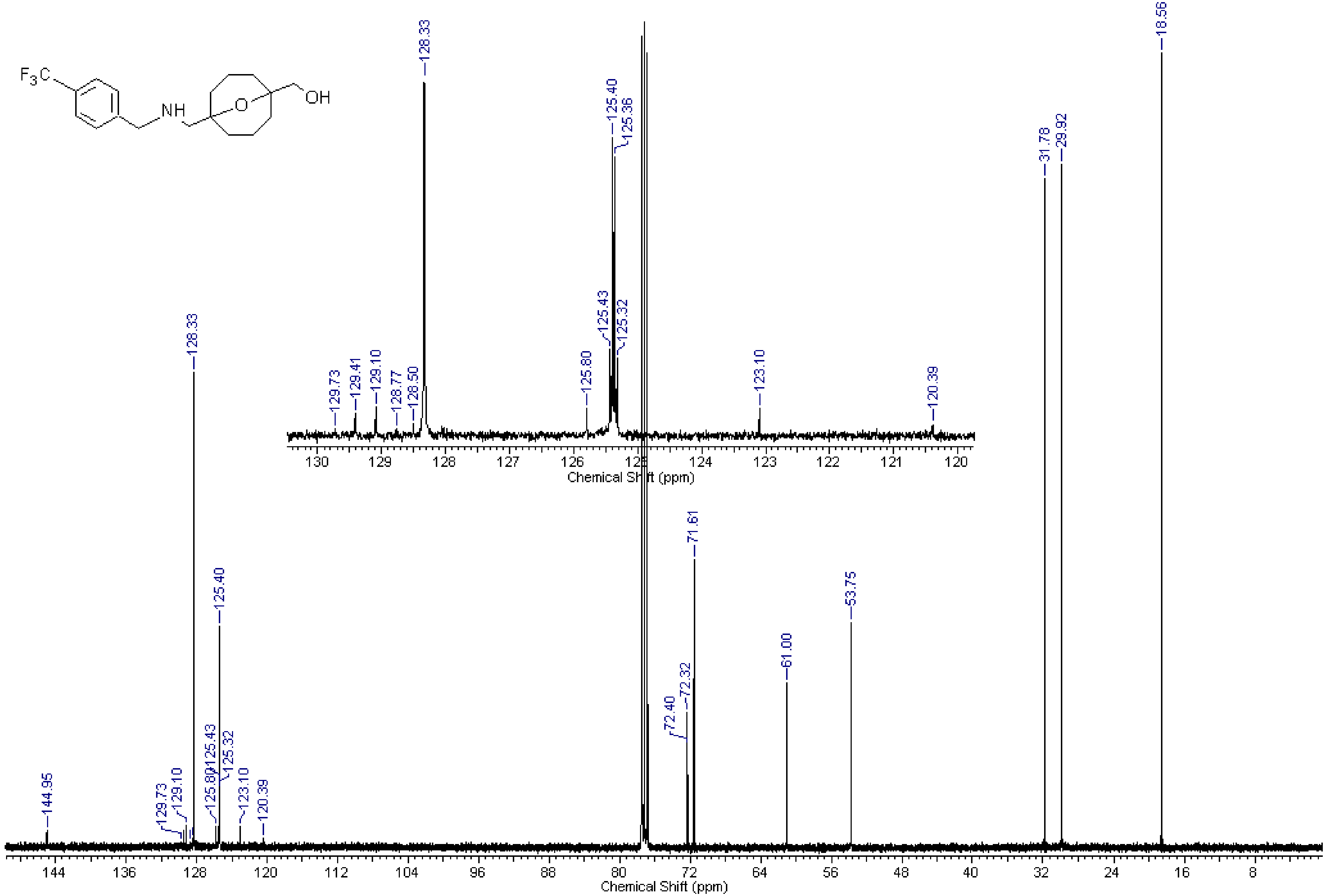

^{19}F NMR spectrum (376 MHz, $CDCl_3$) of compound **5j**

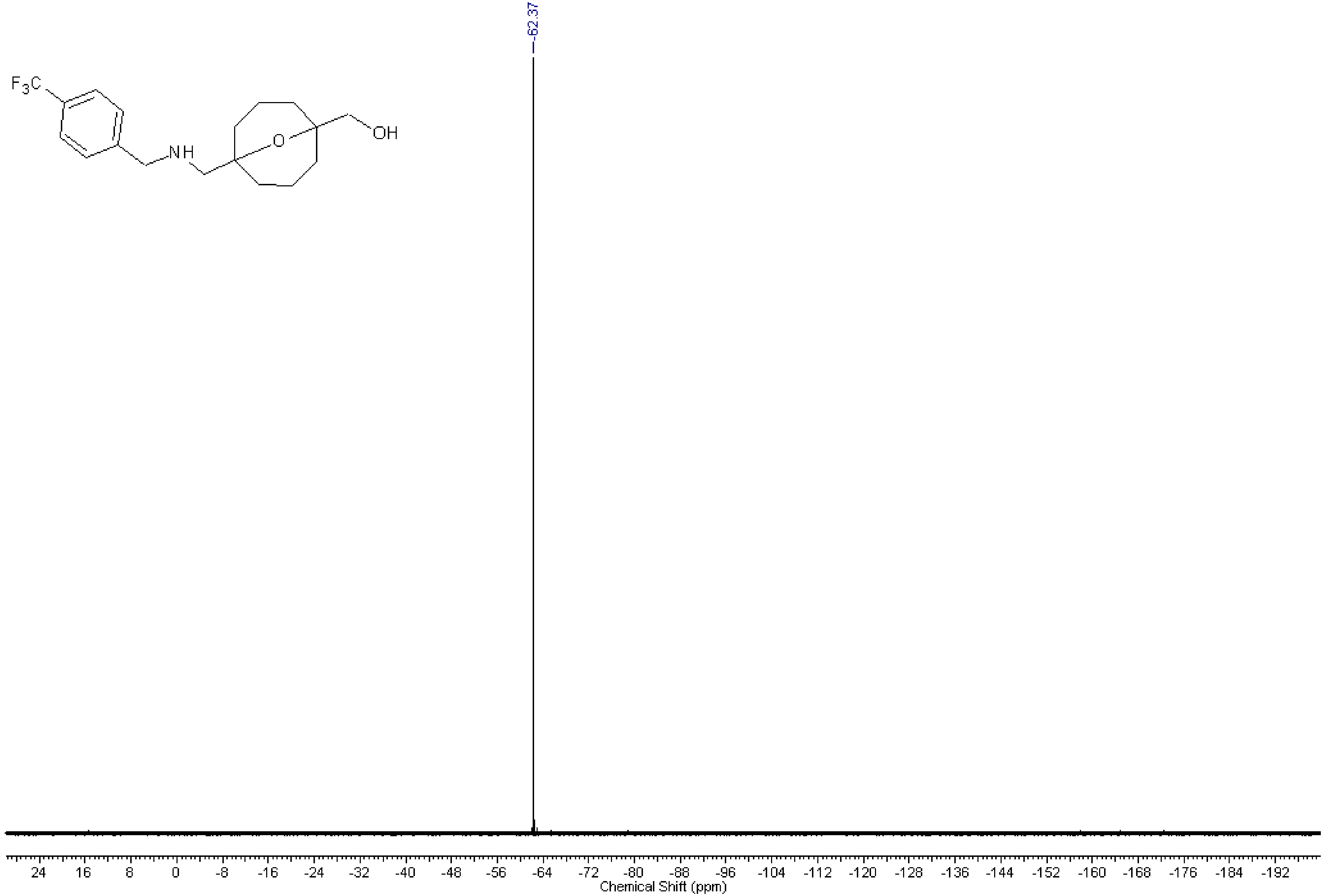

HSQC NMR spectrum ($CDCl_3$) of compound **5j**

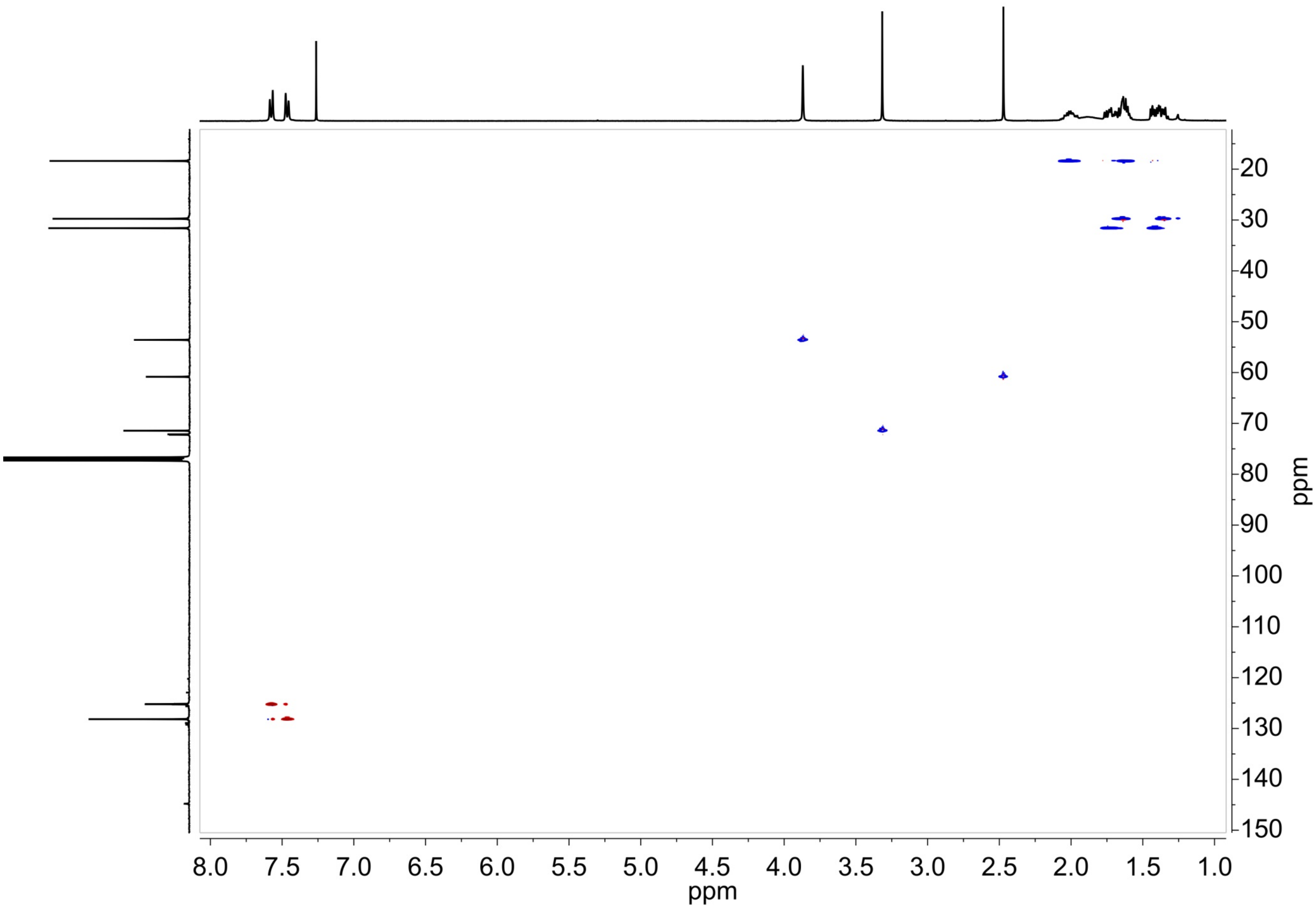

HMBC NMR spectrum ($CDCl_3$) of compound **5j**

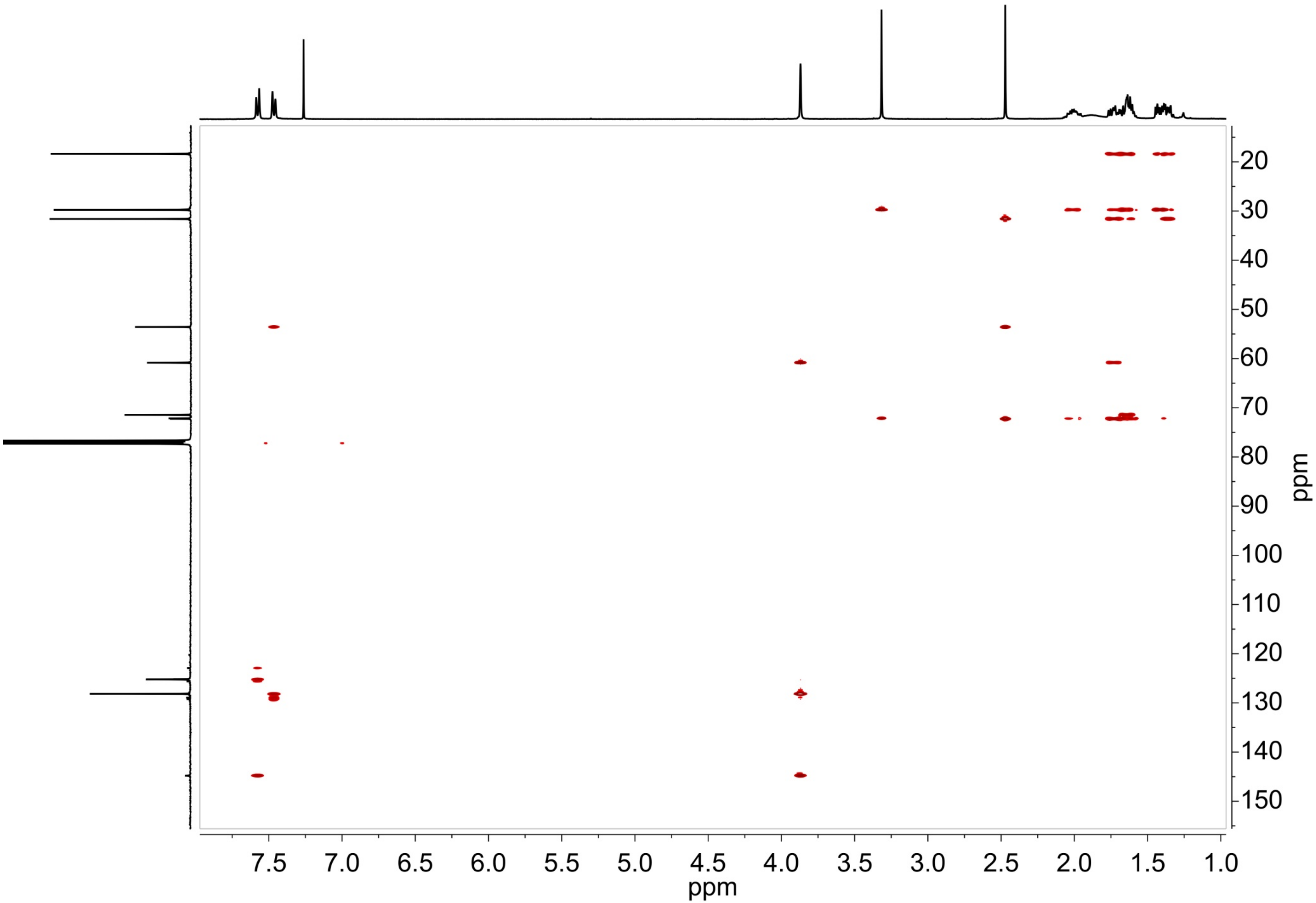

^{1}H NMR spectrum (400 MHz, $CDCl_3$) of compound **5k**

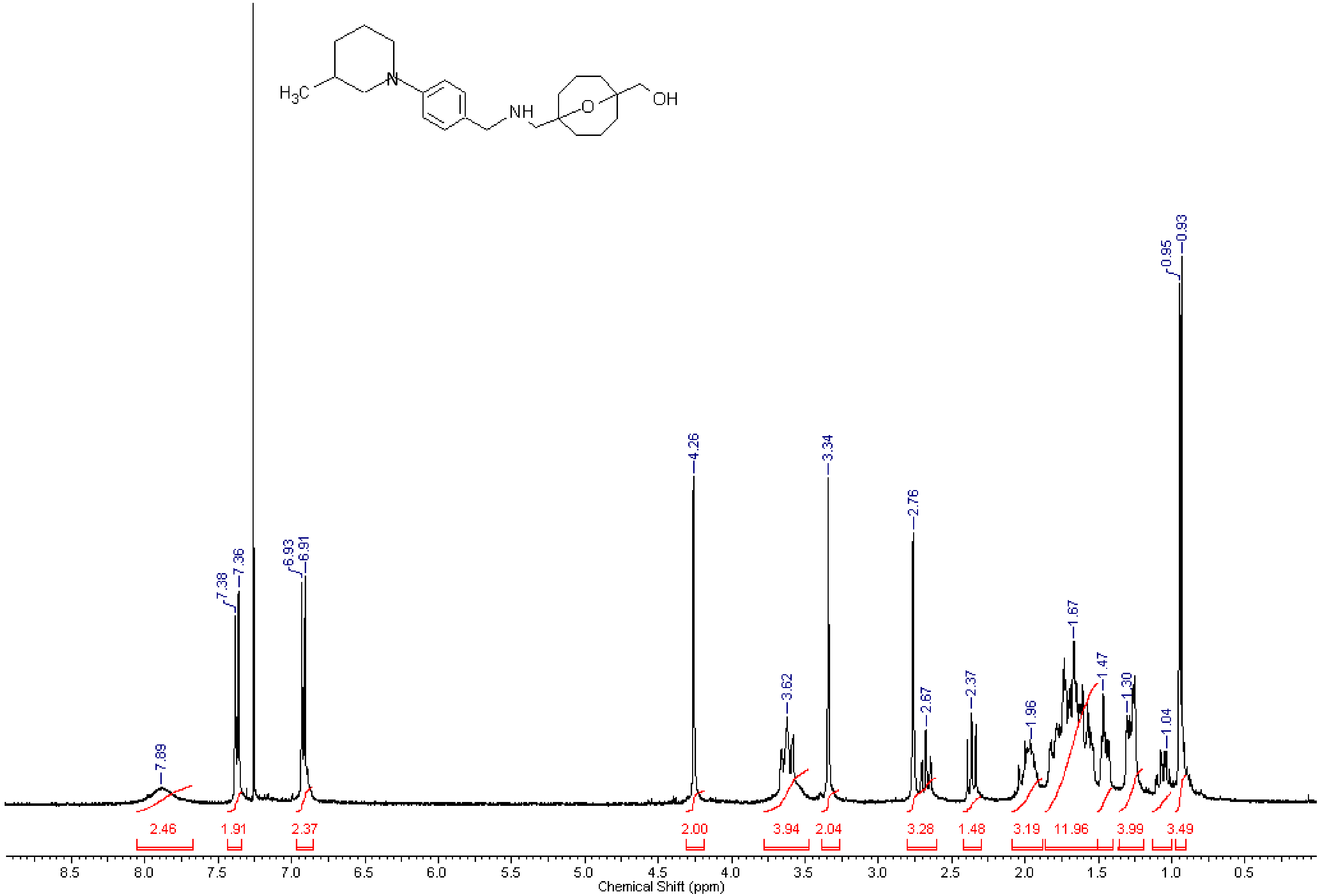

^{13}C NMR spectrum (101 MHz, $CDCl_3$) of compound **5k**

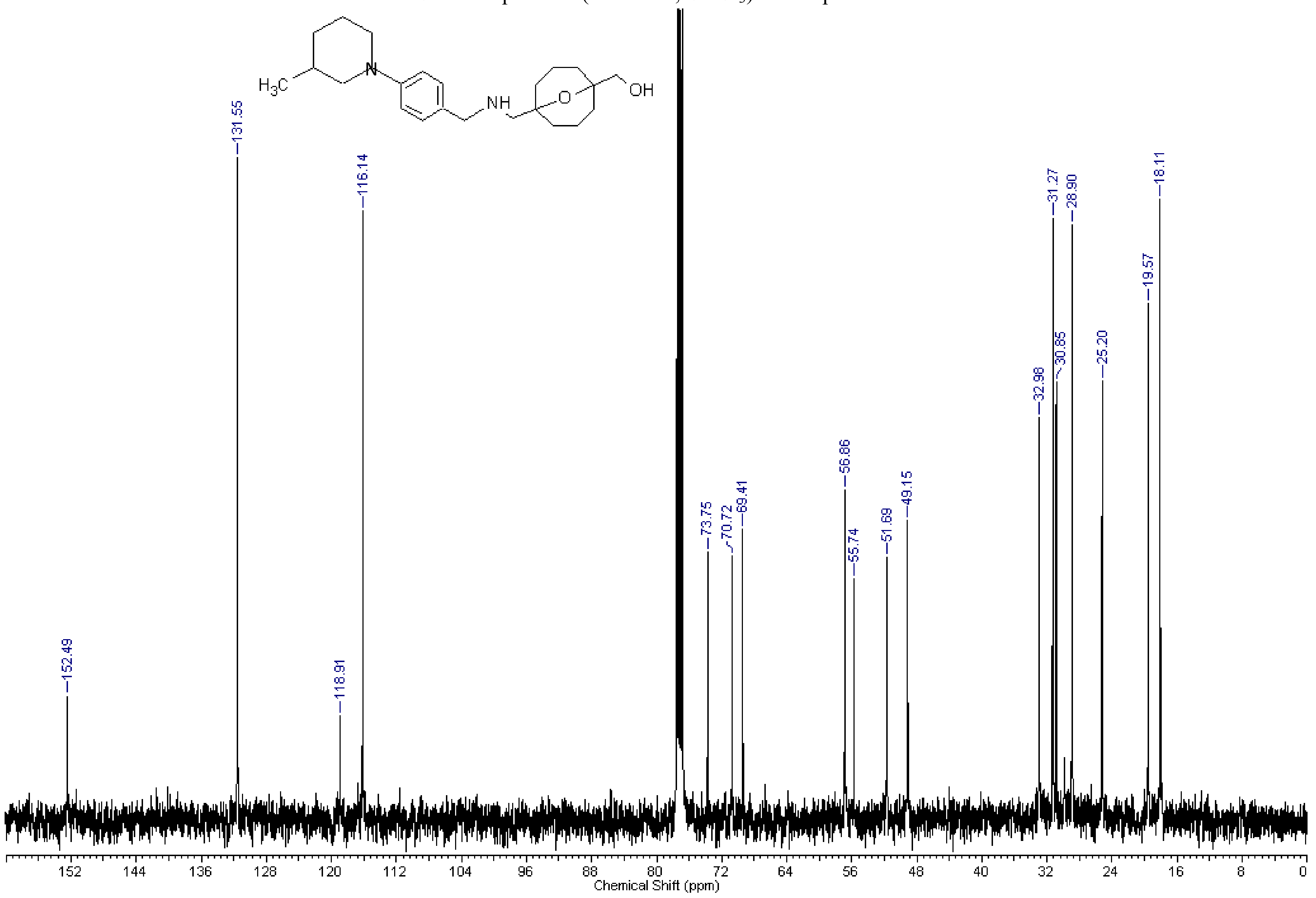

HSQC NMR spectrum of compound **5k**

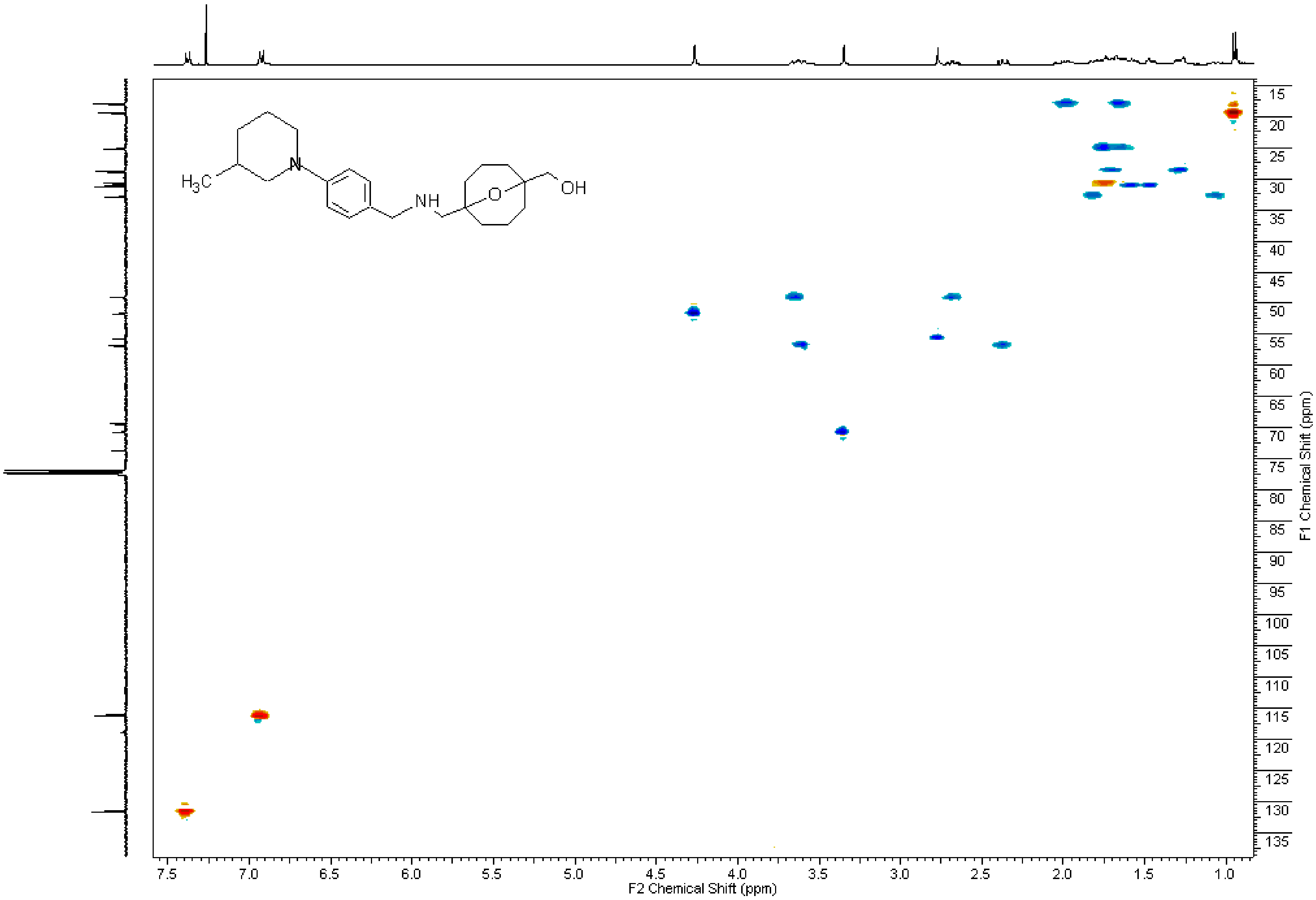

^{1}H NMR spectrum (400 MHz, CDCl$_3$) of compound **5l**

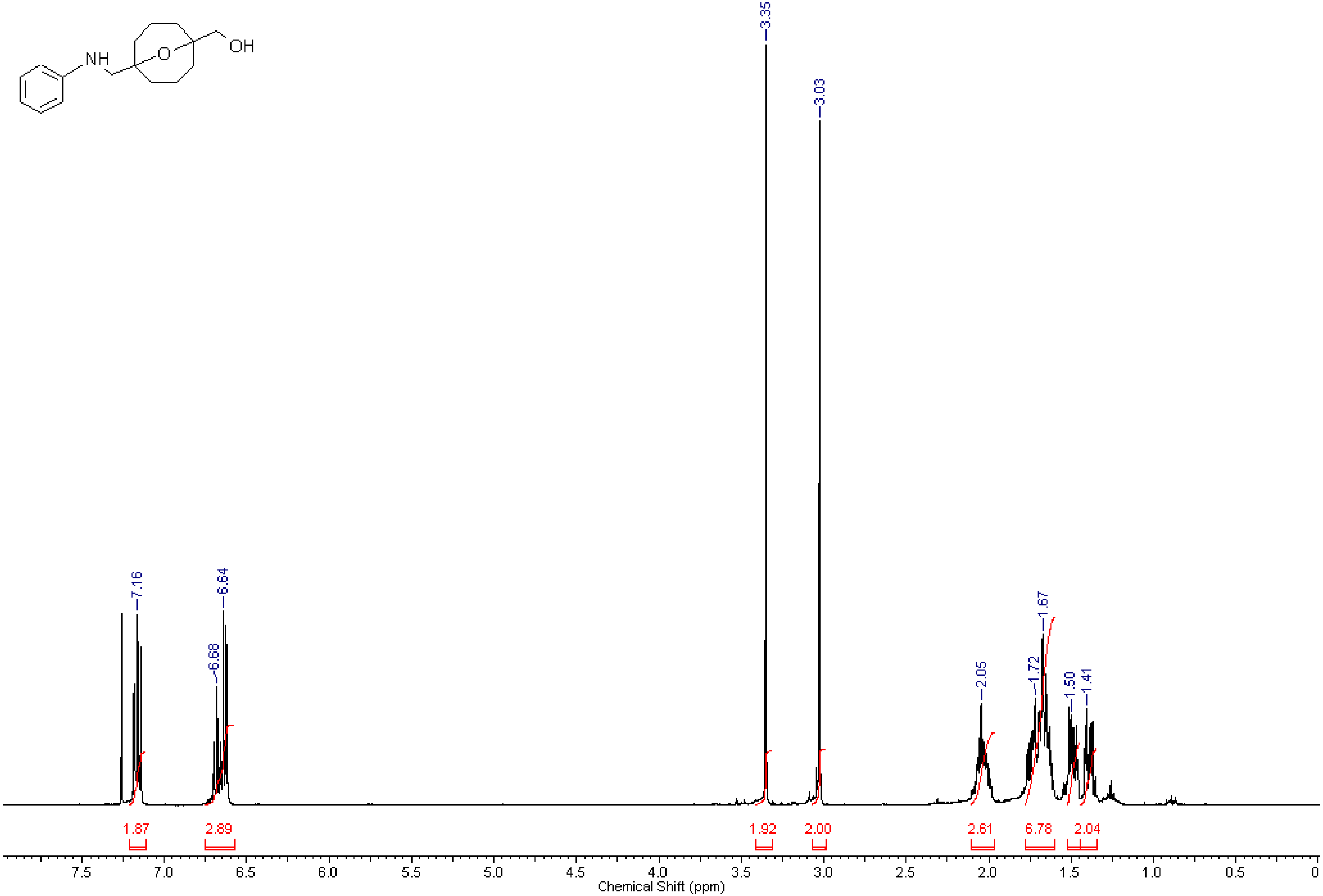

^{13}C NMR spectrum (101 MHz, $CDCl_3$) of compound **5l**

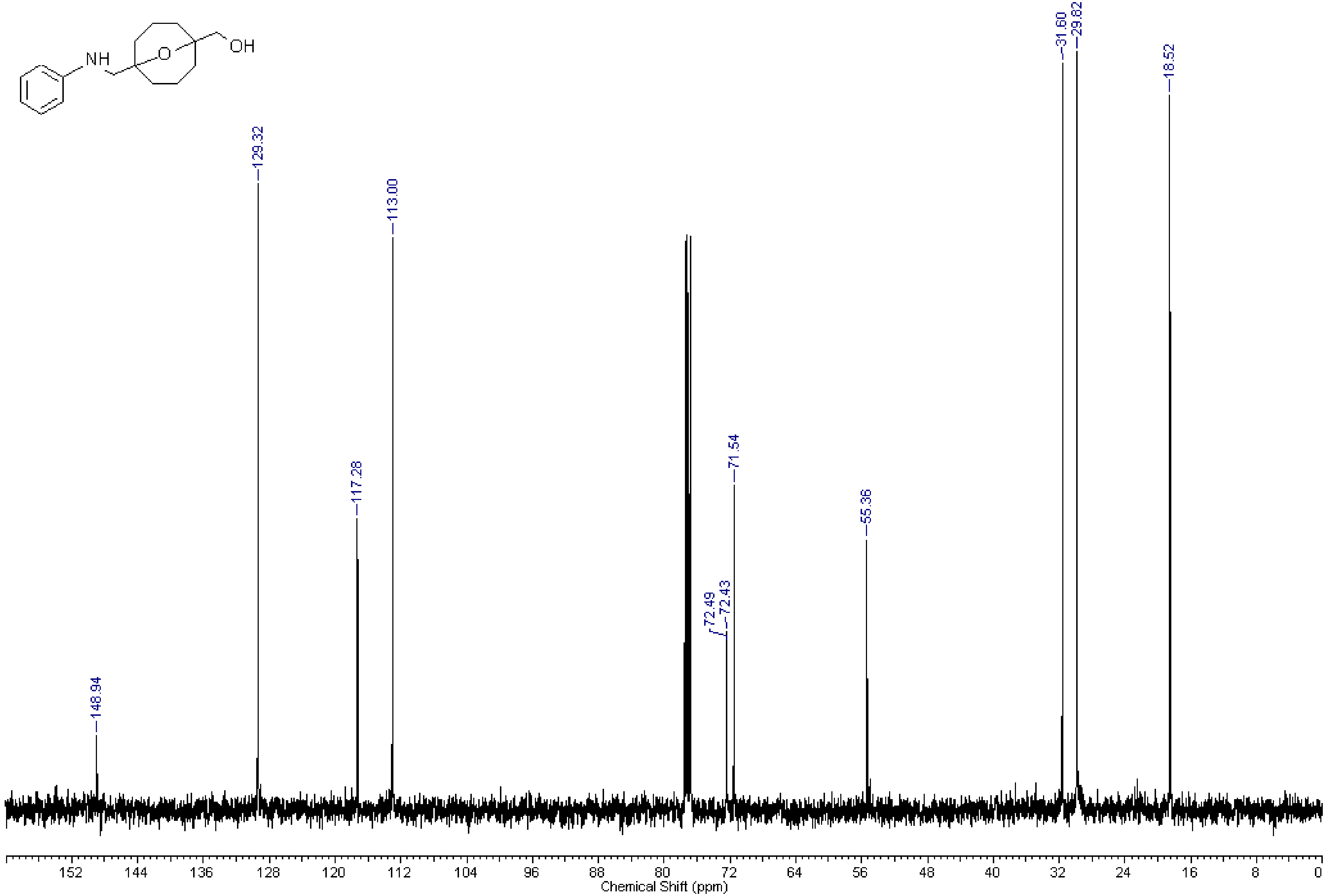

^{1}H NMR spectrum (400 MHz, $CDCl_3$) of compound **5m**

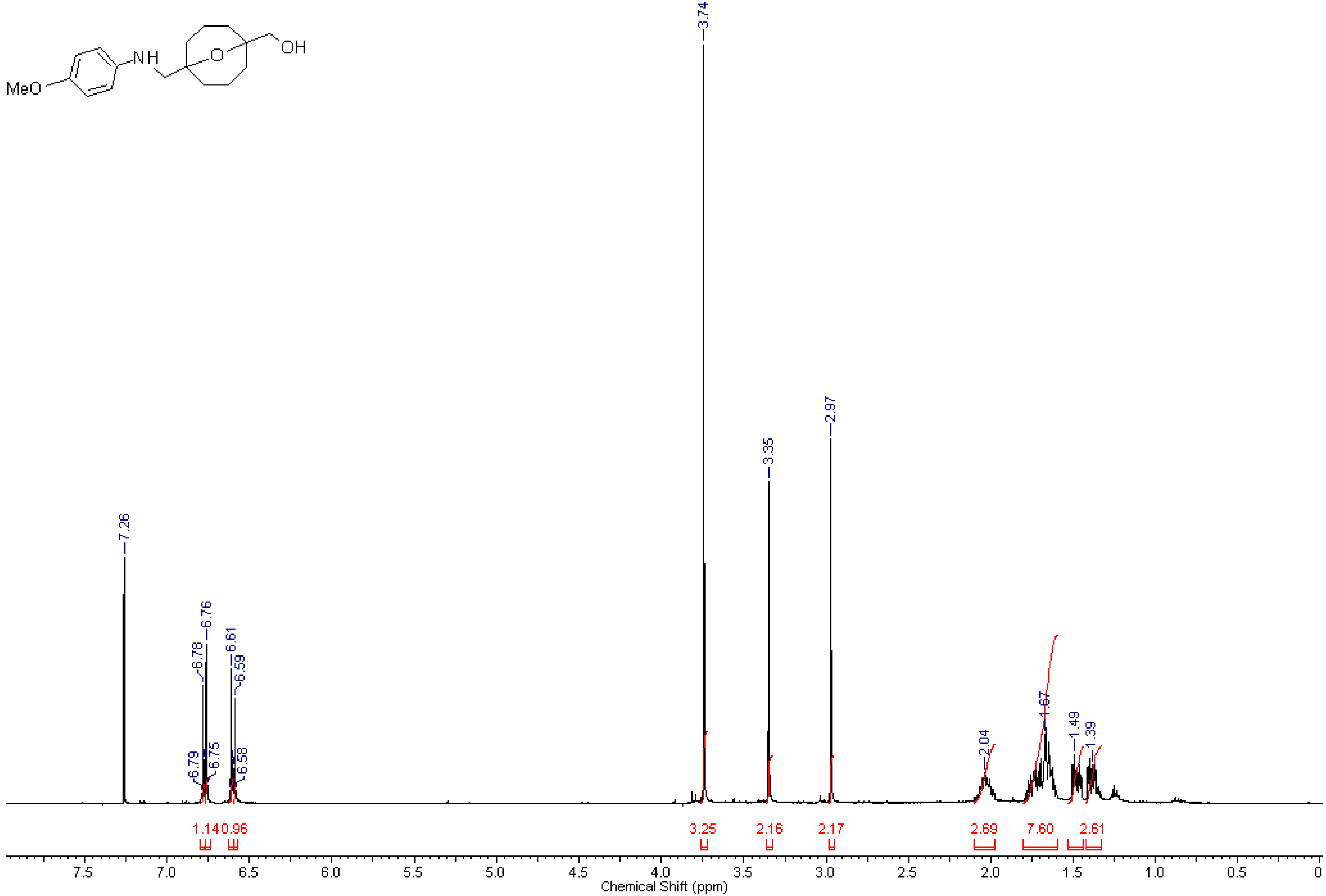

^{13}C NMR spectrum (101 MHz, $CDCl_3$) of compound **5m**

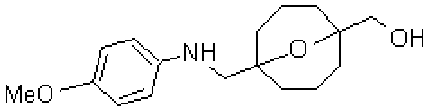

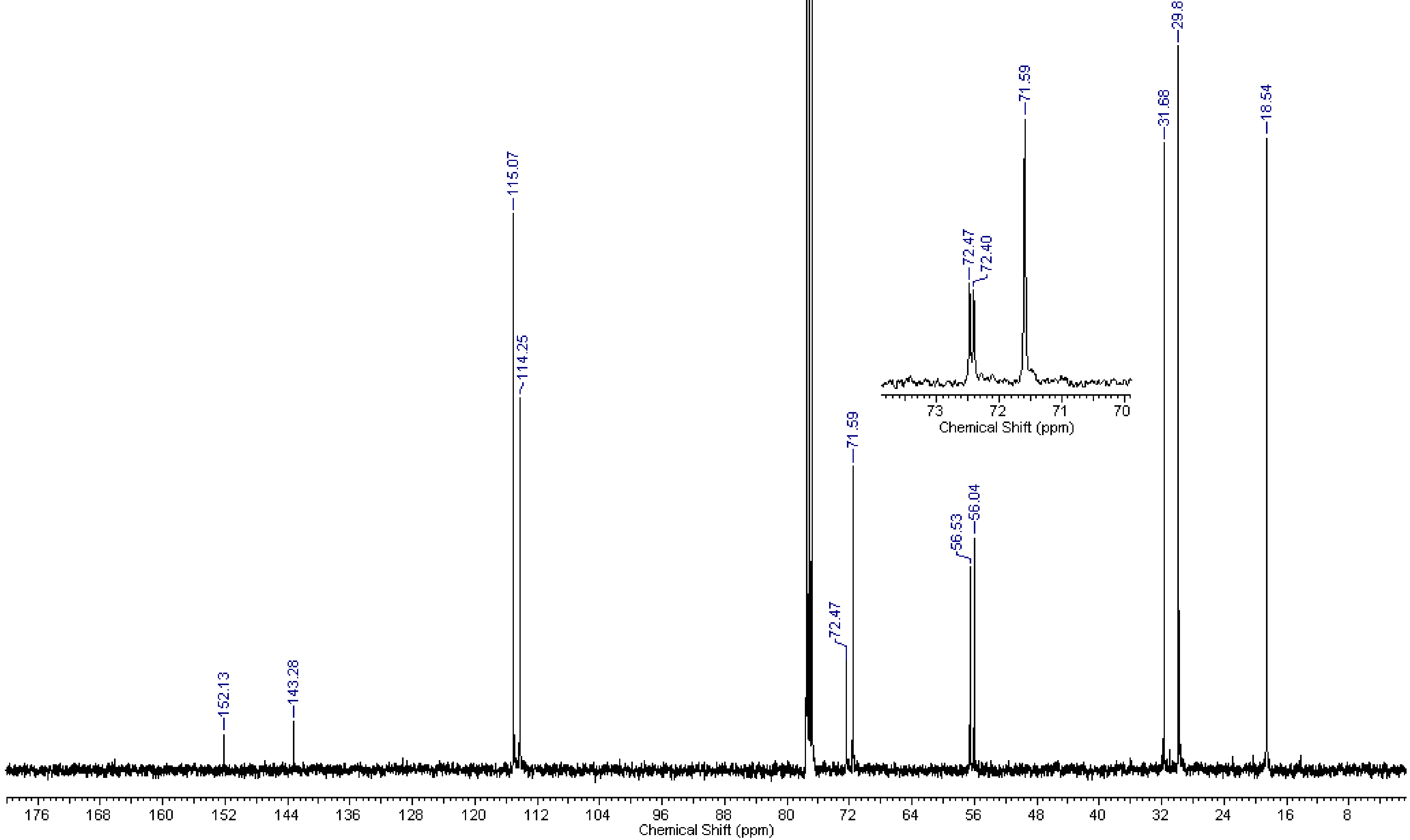

HSQC NMR spectrum of compound **5m**

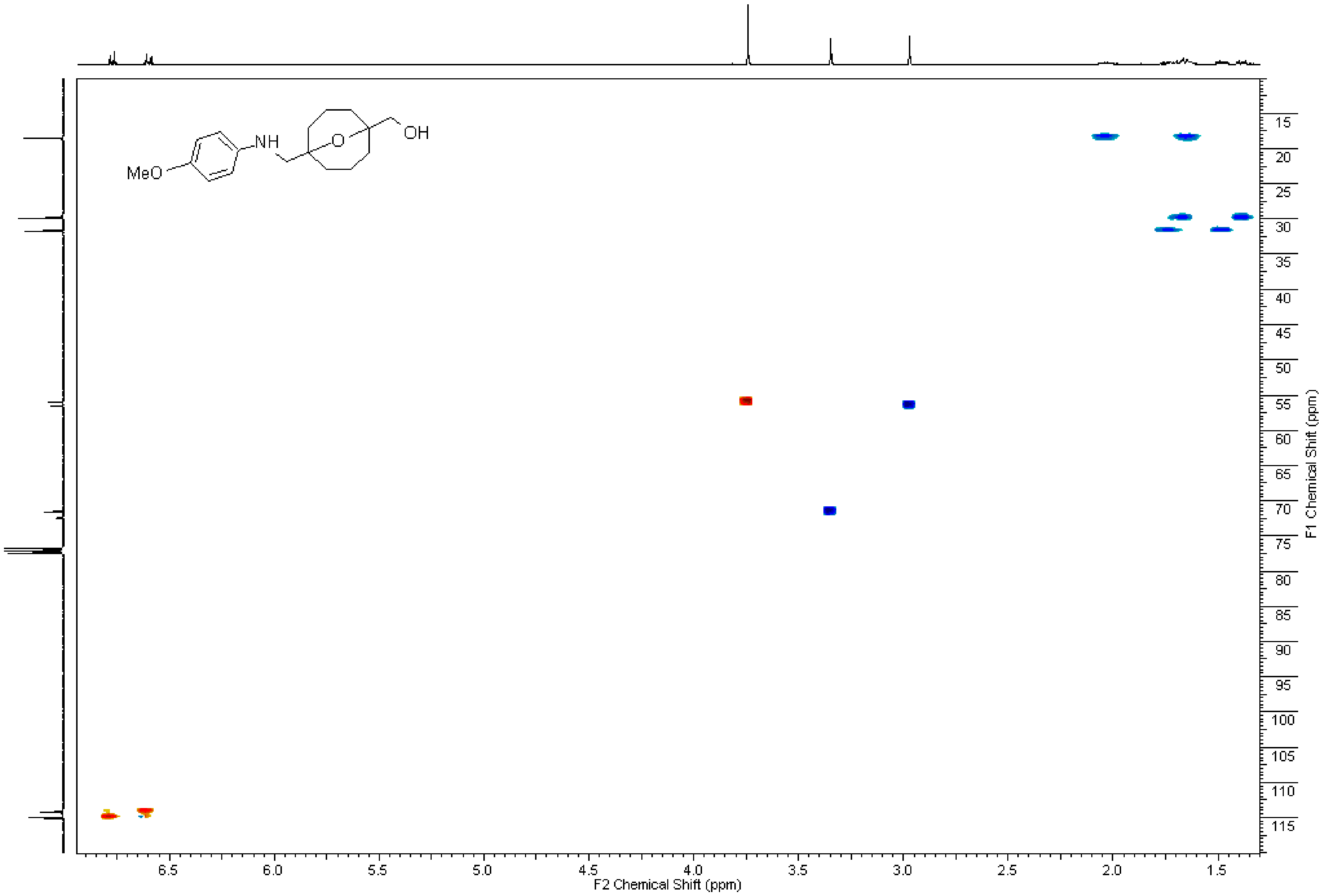

HMBC NMR spectrum of compound **5m**

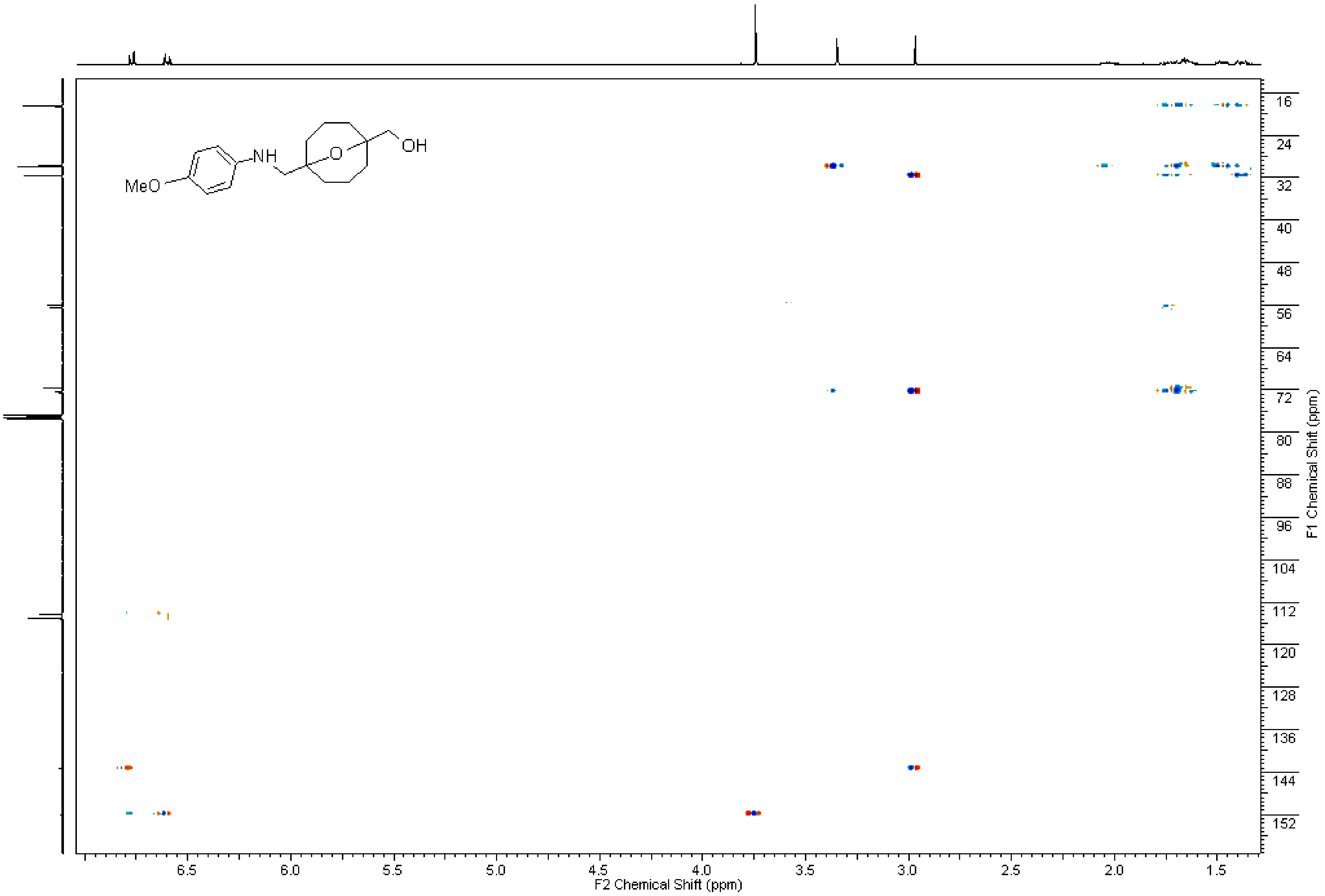

^{1}H NMR spectrum (400 MHz, $CDCl_3$) of compound **5n**

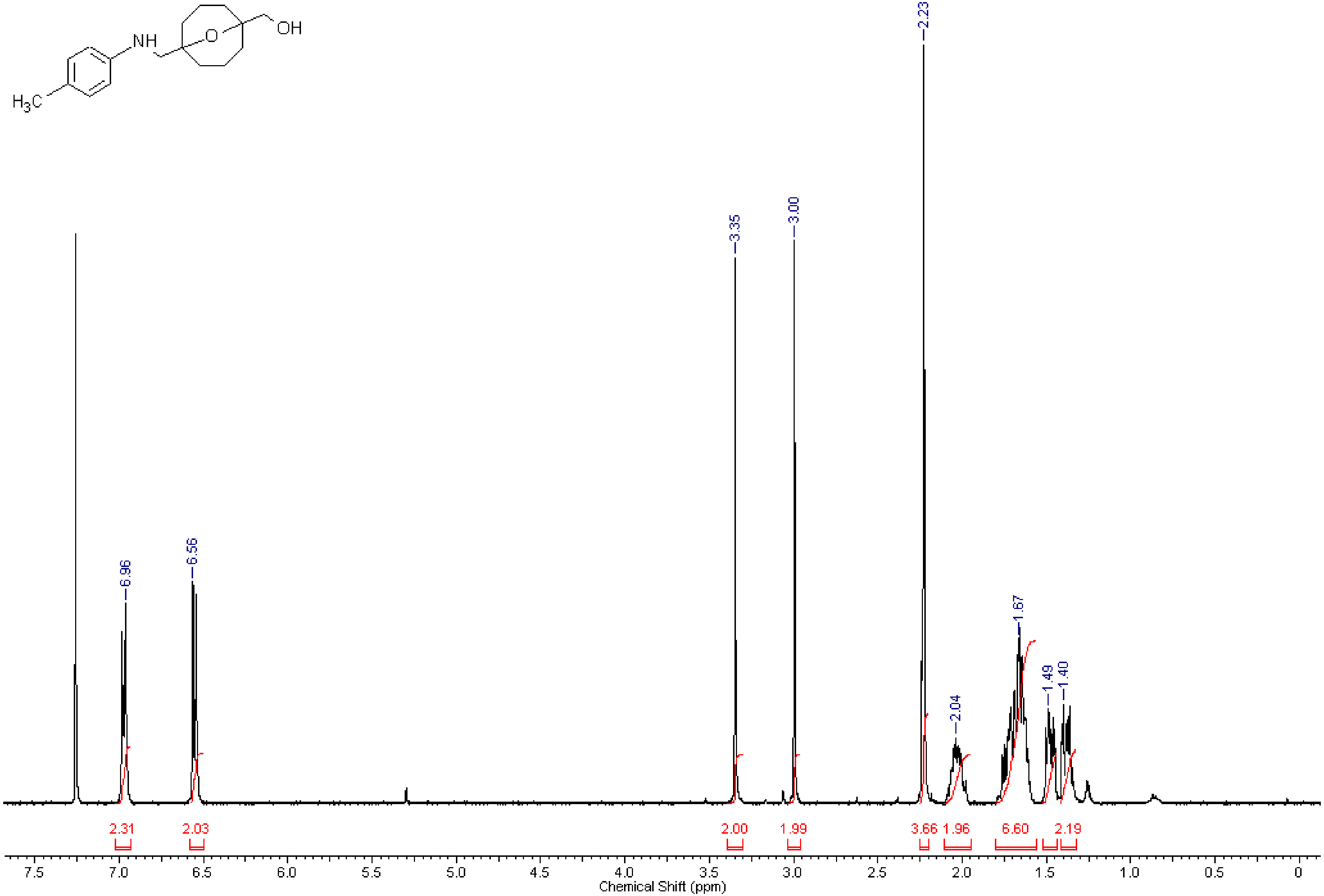

^{13}C NMR spectrum (101 MHz, $CDCl_3$) of compound **5n**

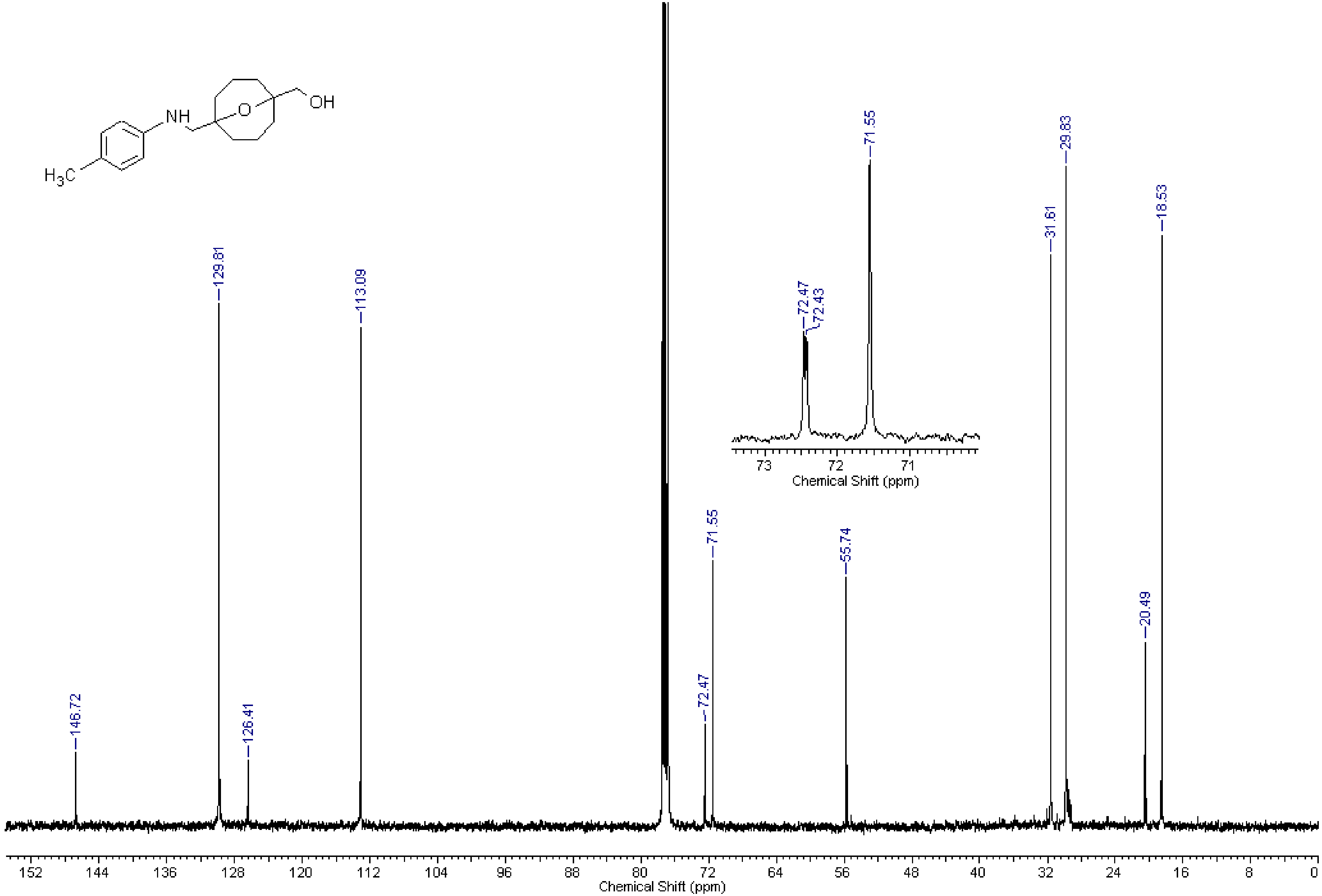

HSQC NMR spectrum of compound **5n**

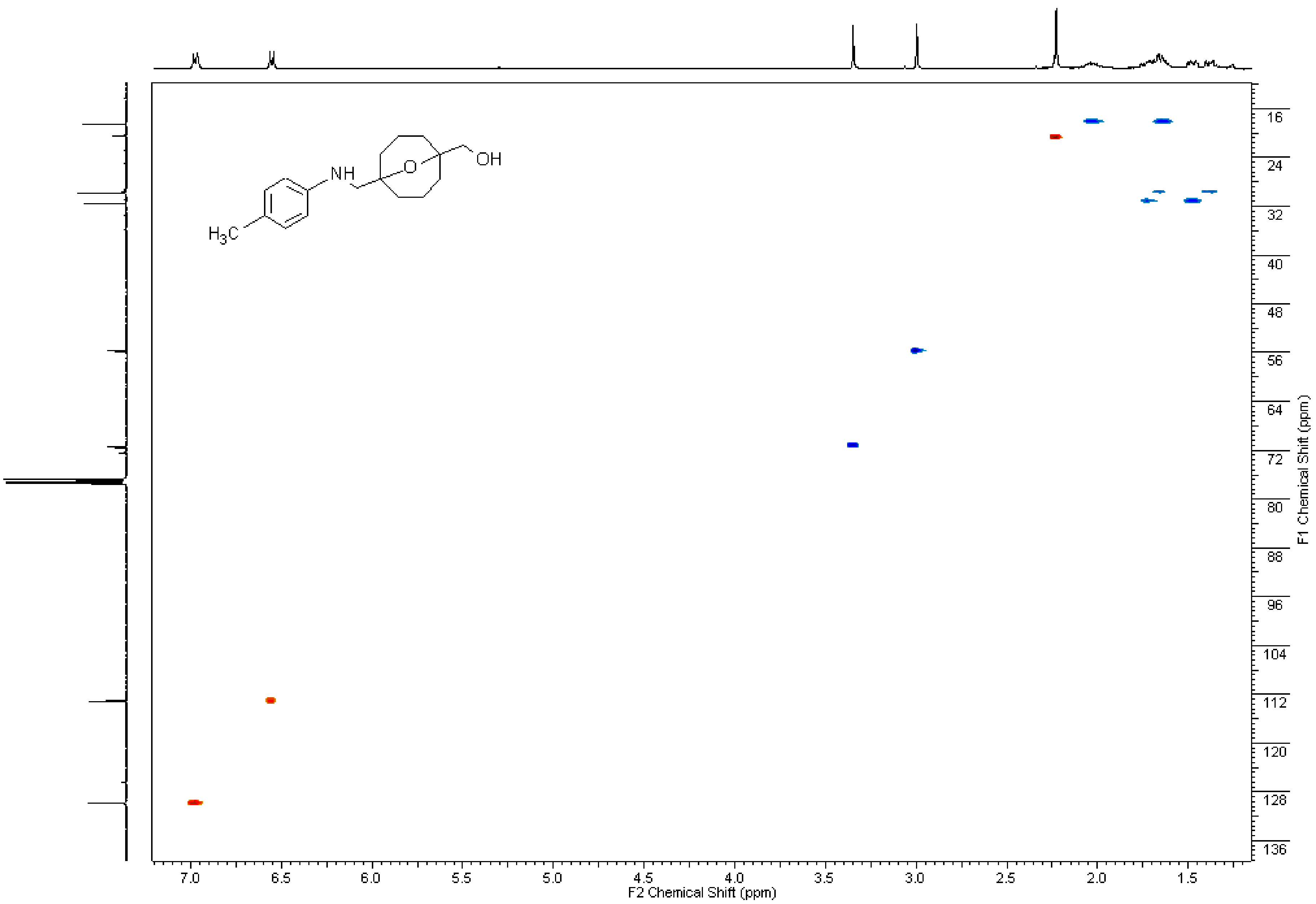

^{1}H NMR spectrum (400 MHz, $CDCl_3$) of compound **5o**

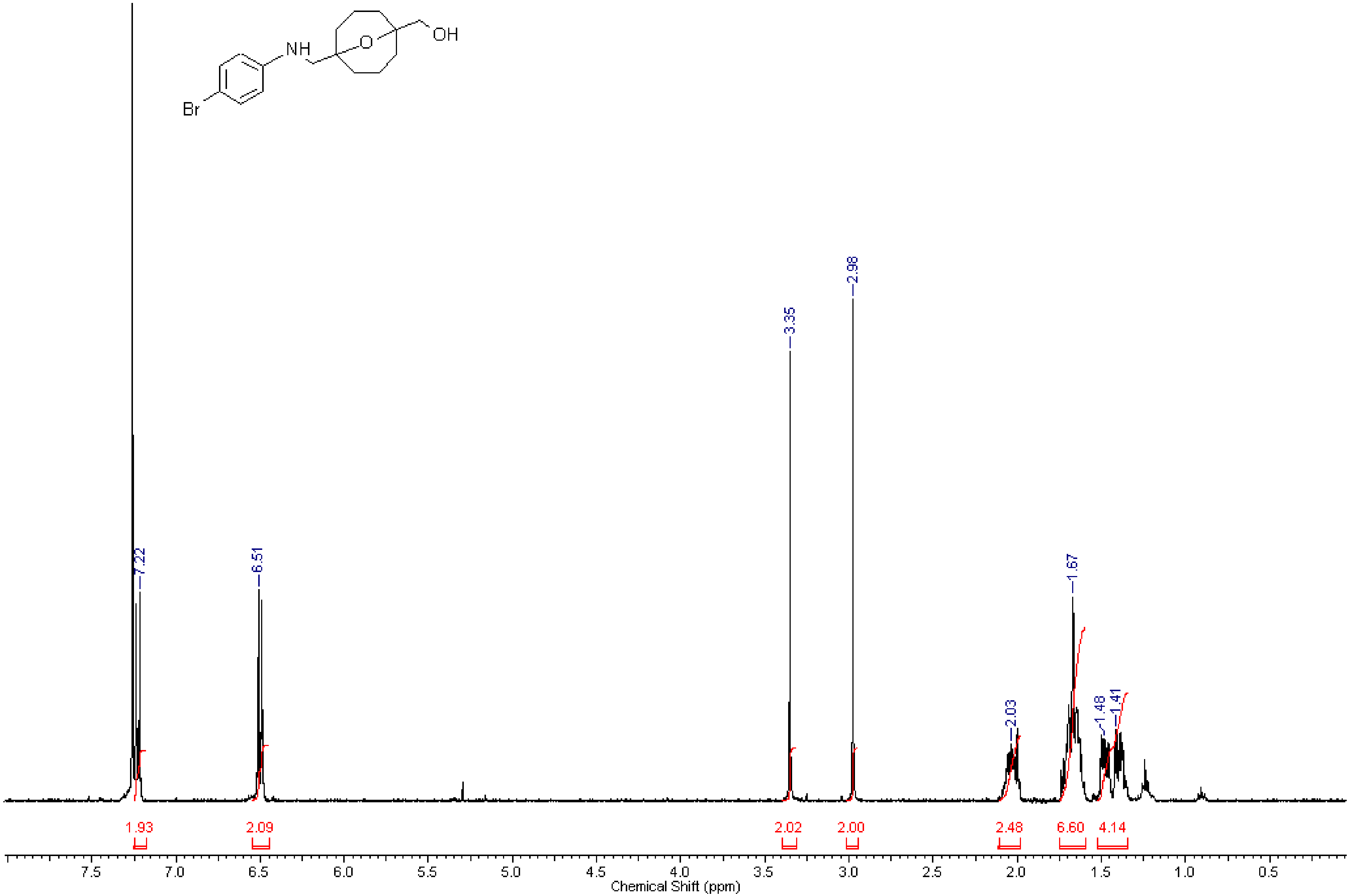

^{13}C NMR spectrum (101 MHz, $CDCl_3$) of compound **5o**

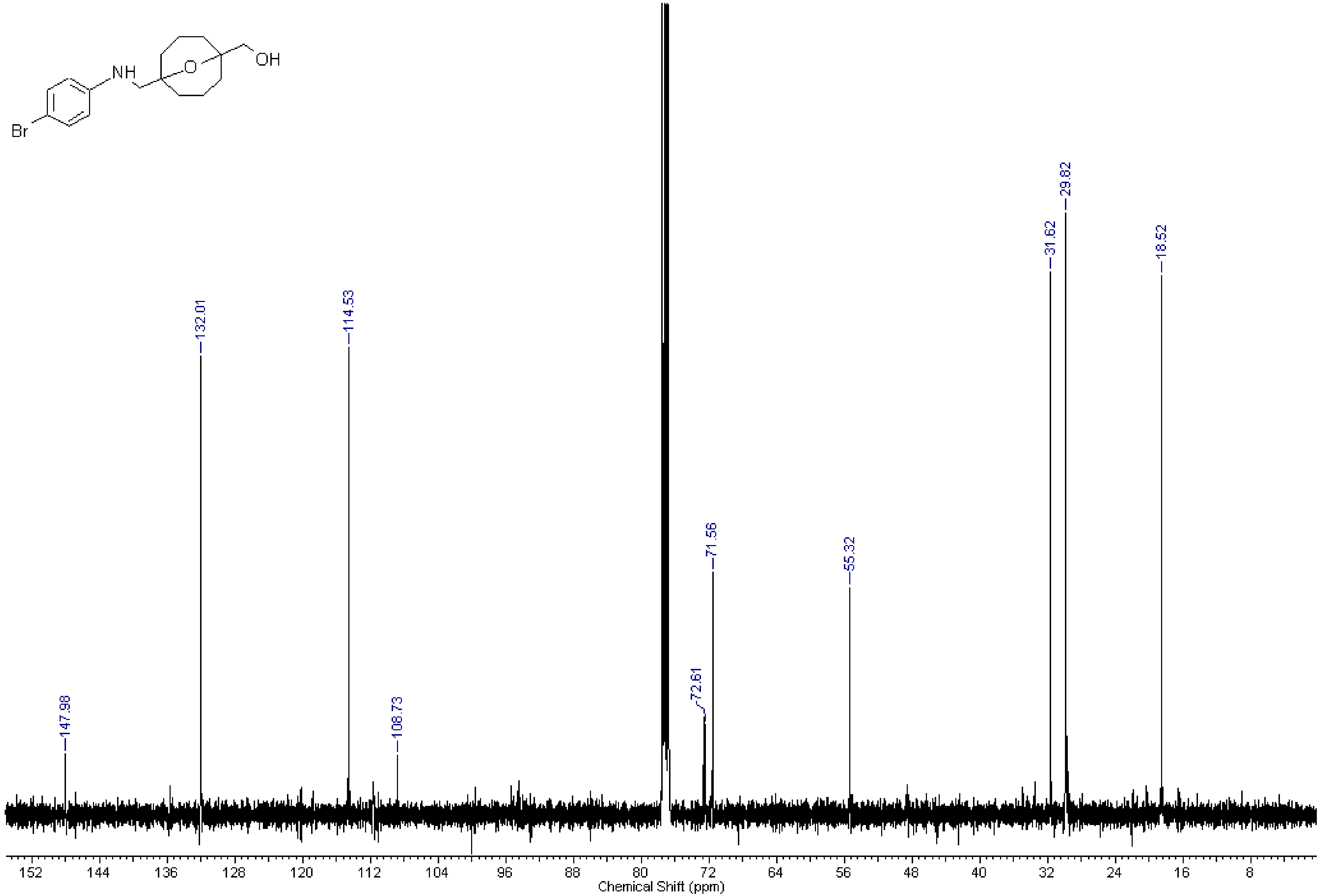

^{1}H NMR spectrum (400 MHz, $CDCl_3$) of compound **6a**

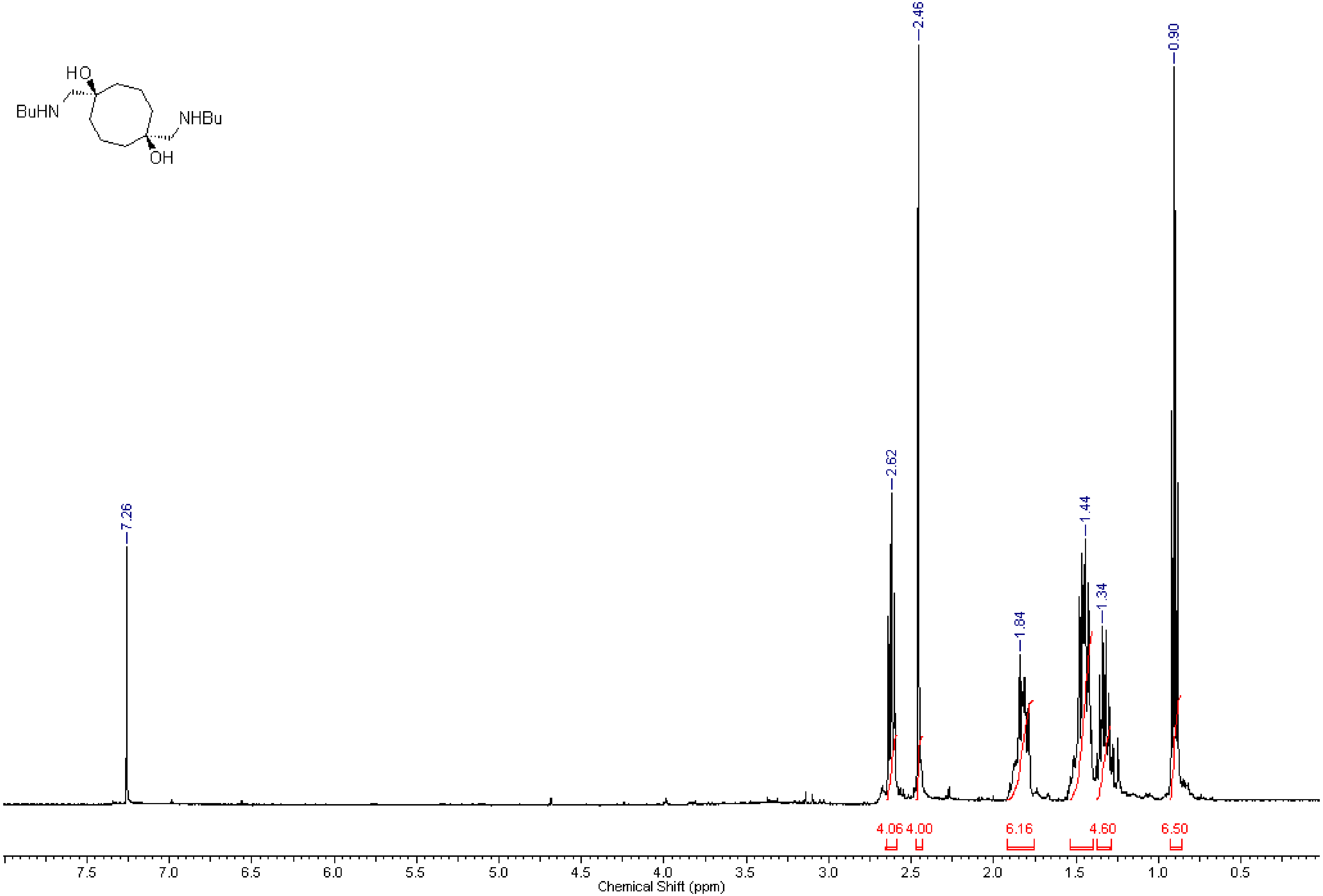

^{13}C NMR spectrum (101 MHz, $CDCl_3$) of compound **6a**

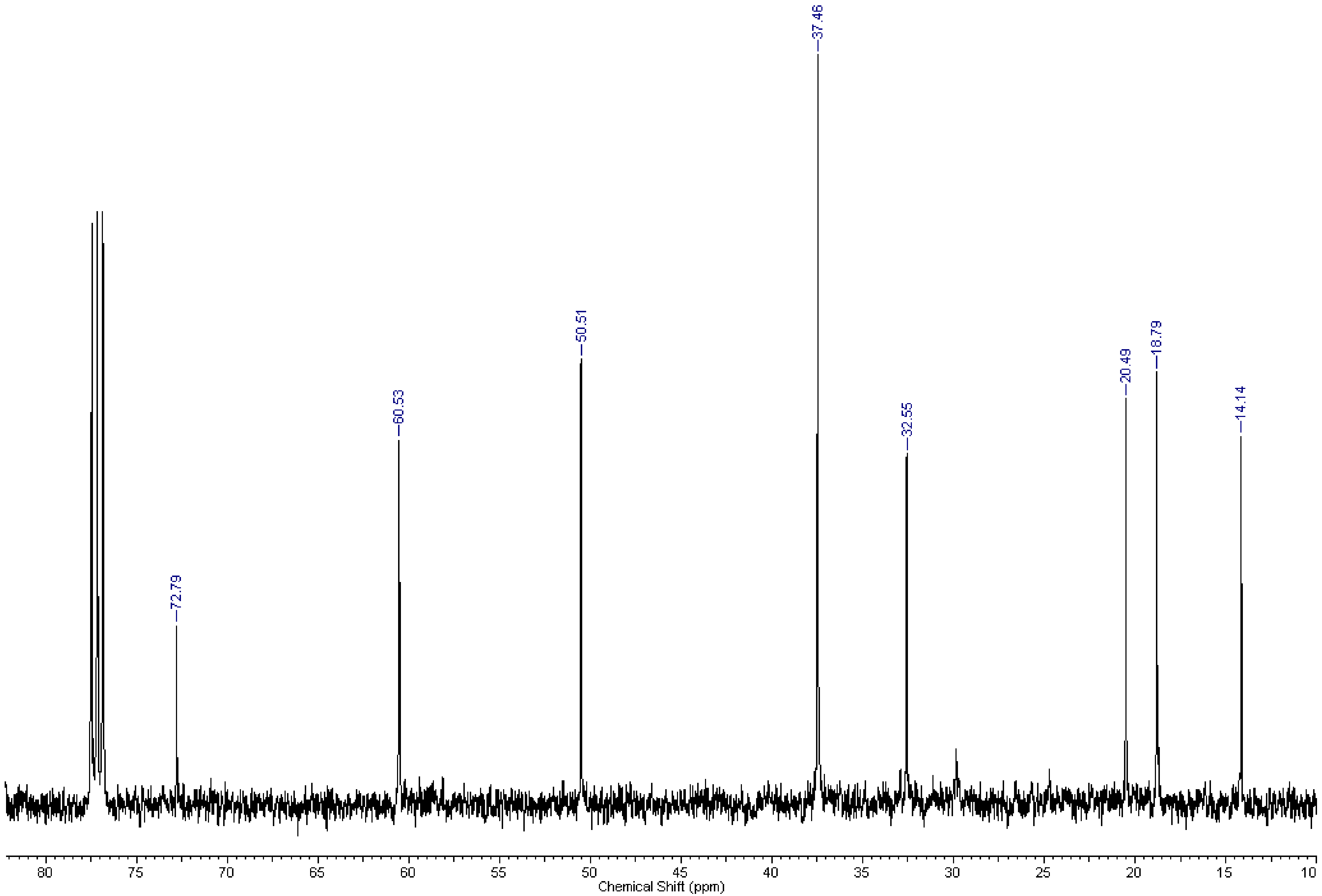

^{1}H NMR spectrum (400 MHz, $CDCl_3$) of compound **6b**

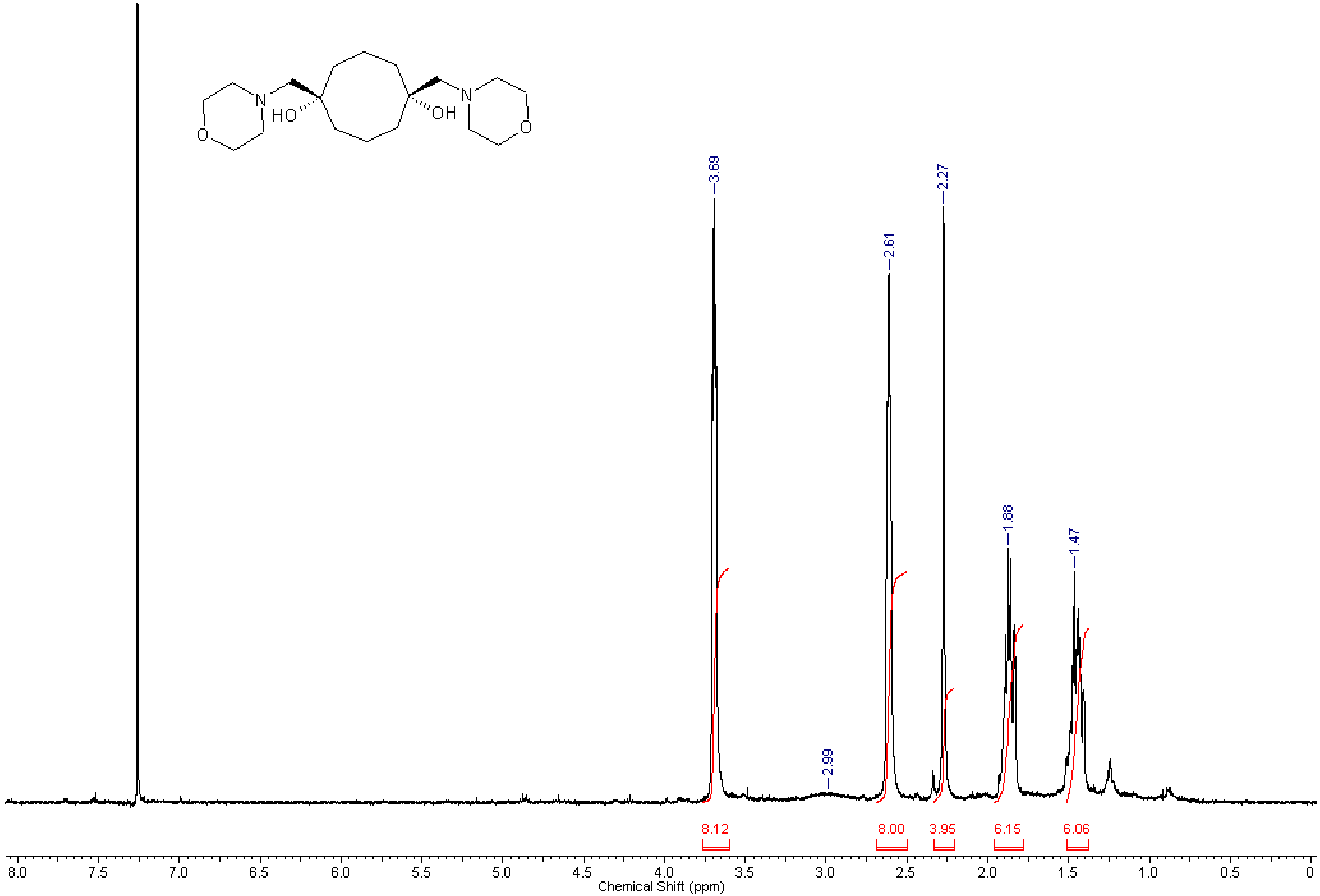

^{13}C NMR spectrum (101 MHz, $CDCl_3$) of compound **6b**

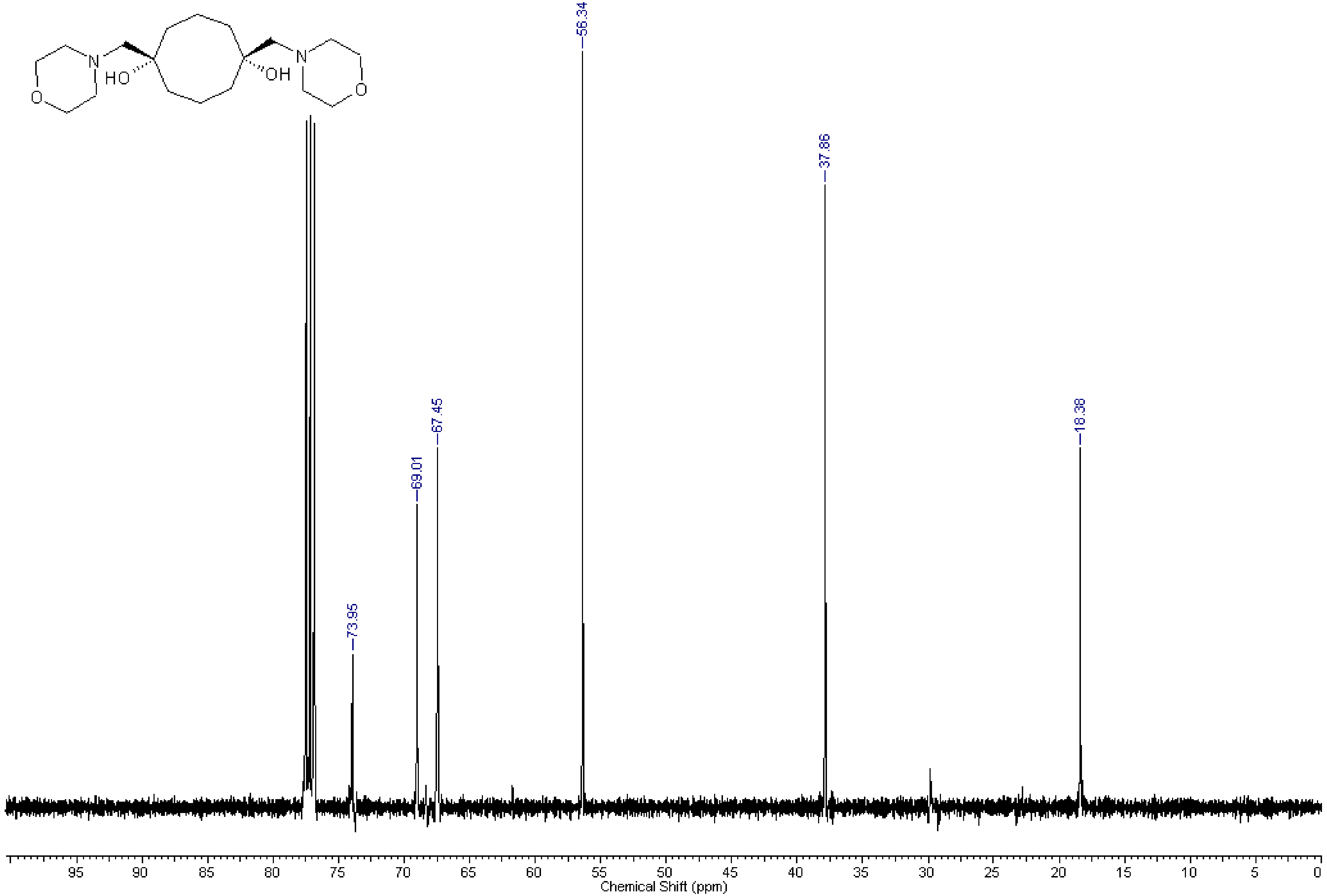

HSQC NMR spectrum of compound **6b**

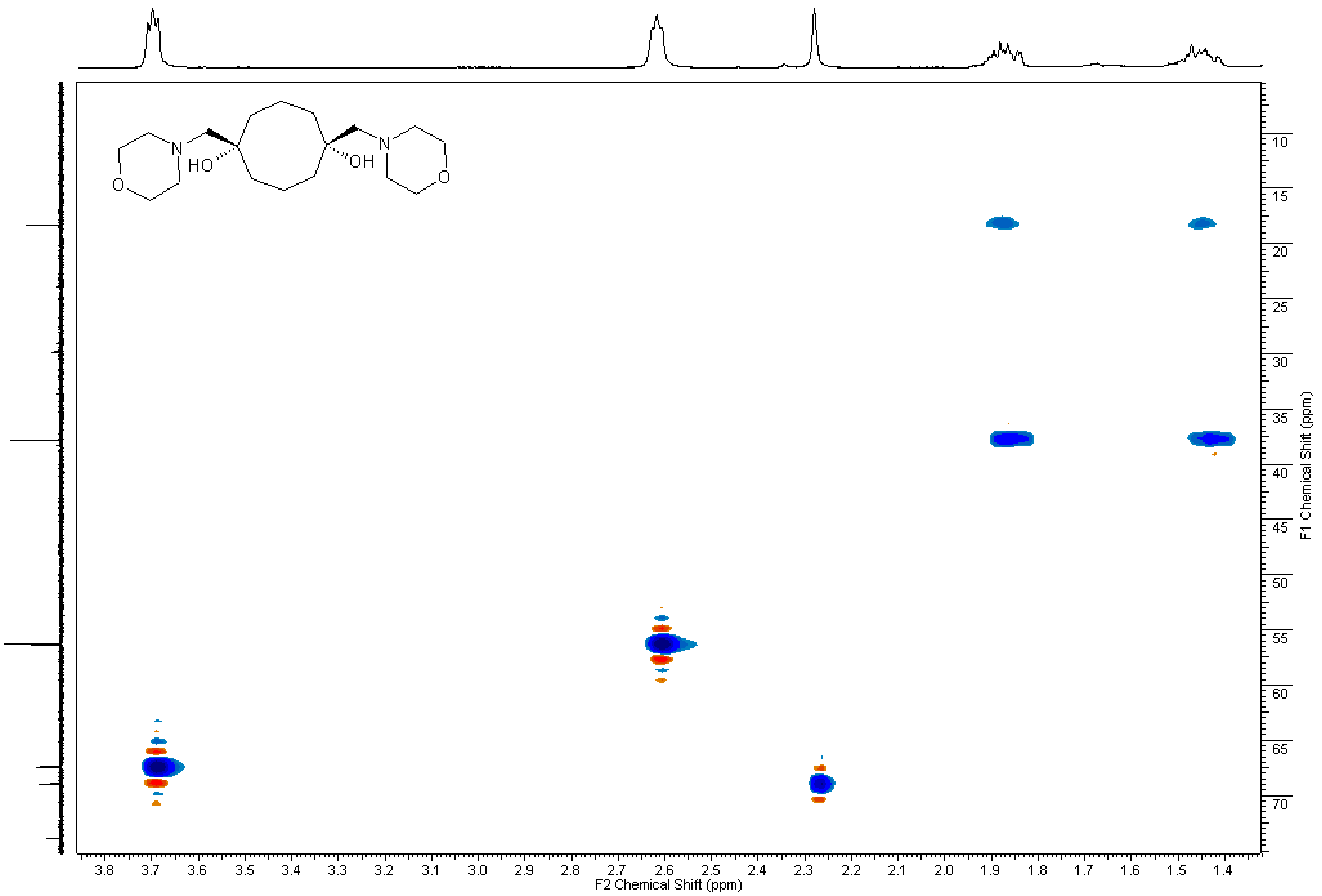

^{1}H NMR spectrum (400 MHz, $CDCl_3$) of compound **6c**

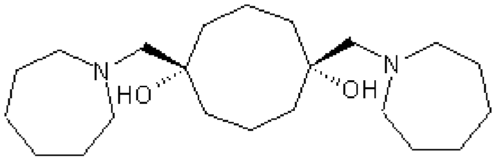

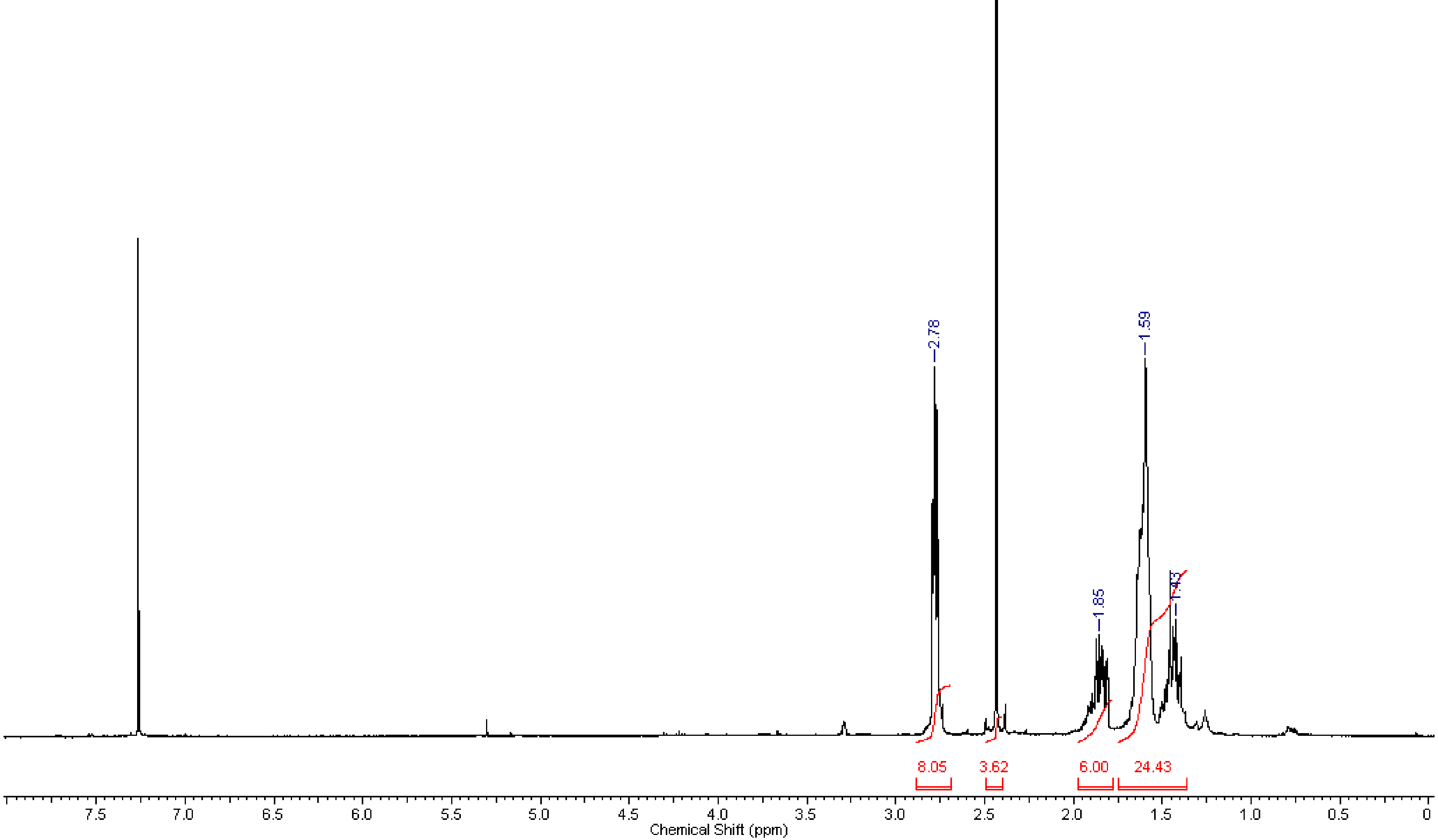

^{13}C NMR spectrum (101 MHz, $CDCl_3$) of compound **6c**

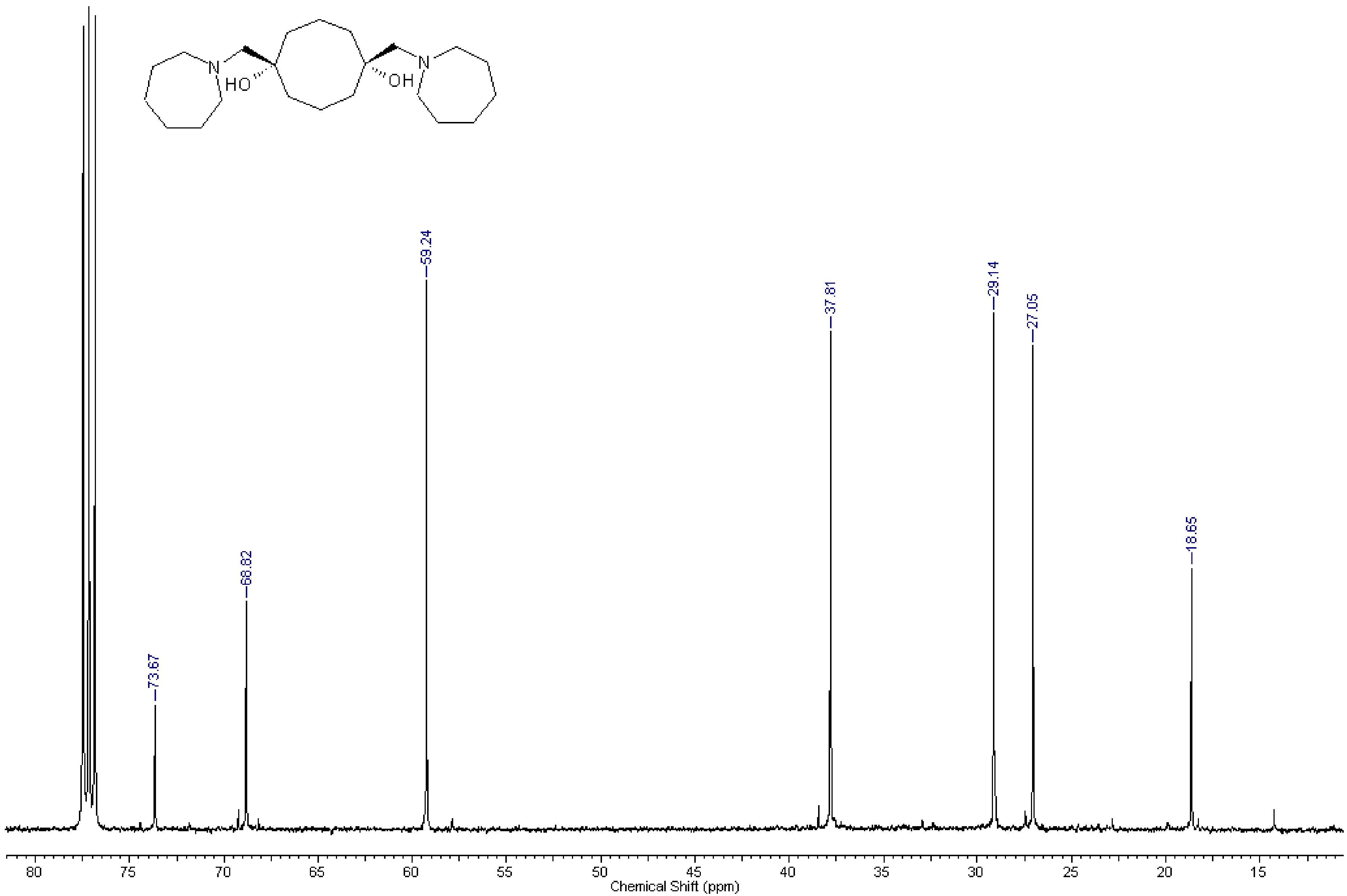

HSQC NMR spectrum of compound **6c**

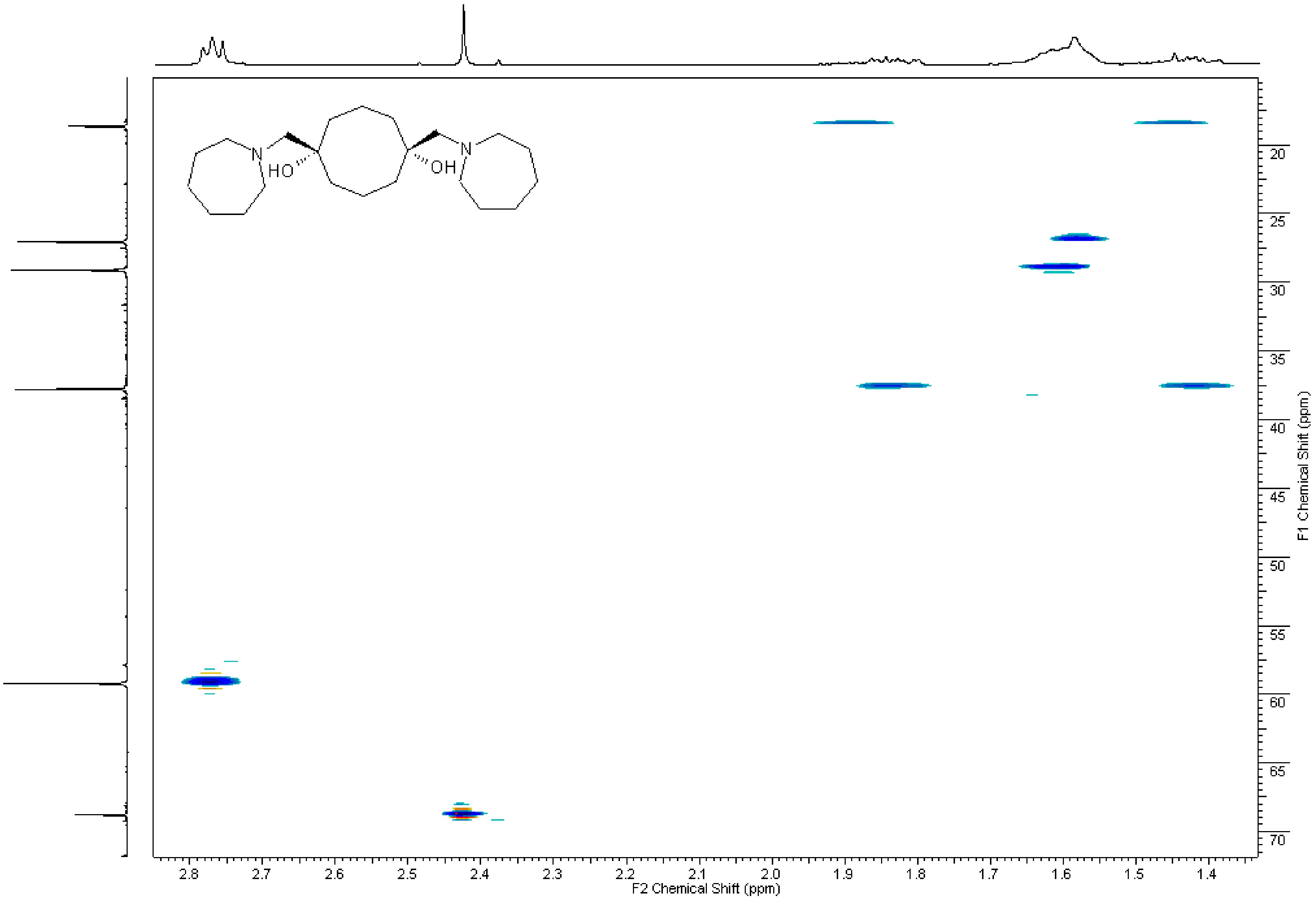

^{1}H NMR spectrum (400 MHz, $CDCl_3$) of compound **6d**

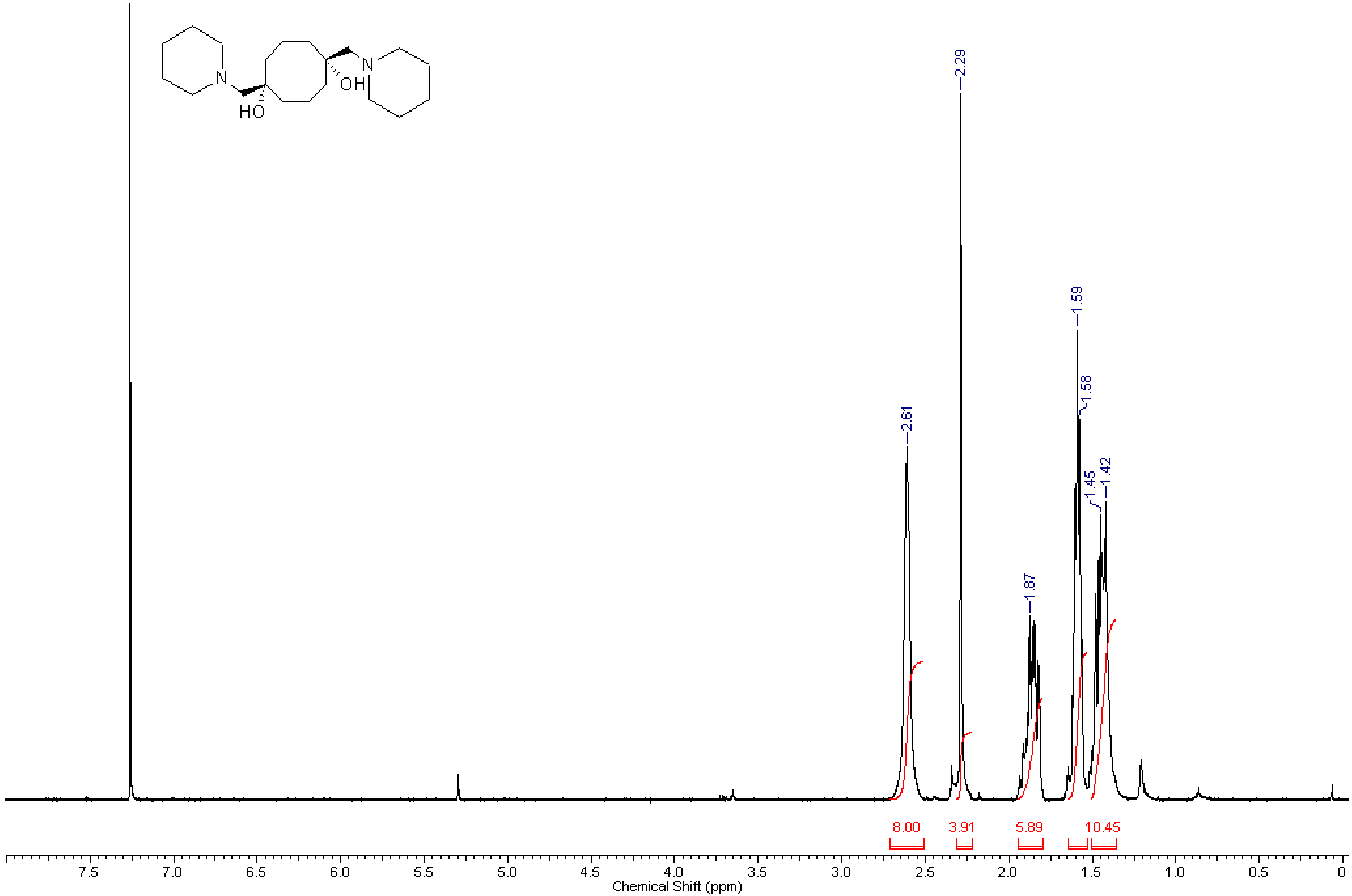

^{13}C NMR spectrum (101 MHz, CDCl$_3$) of compound **6d**

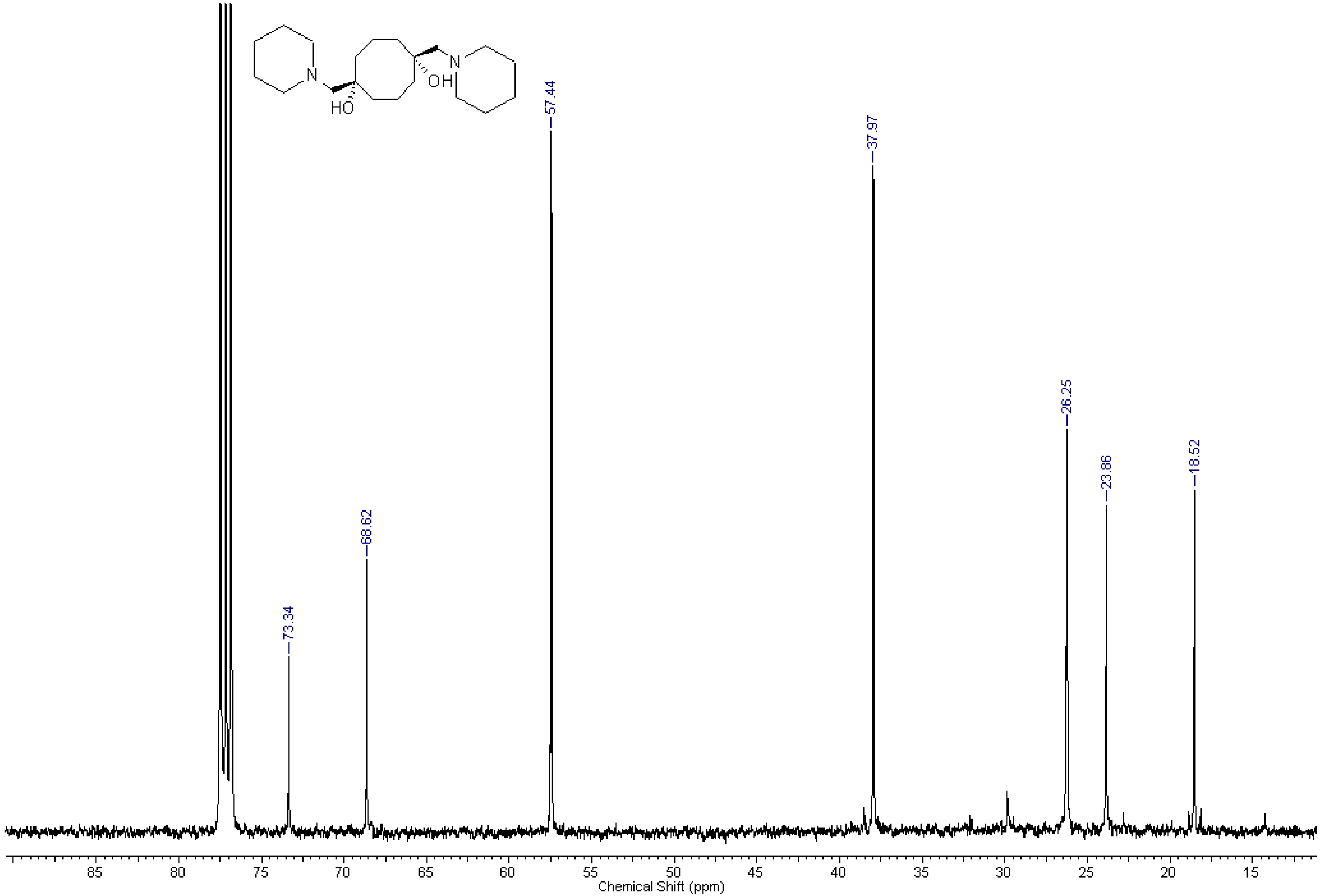

HSQC NMR spectrum of compound **6d**

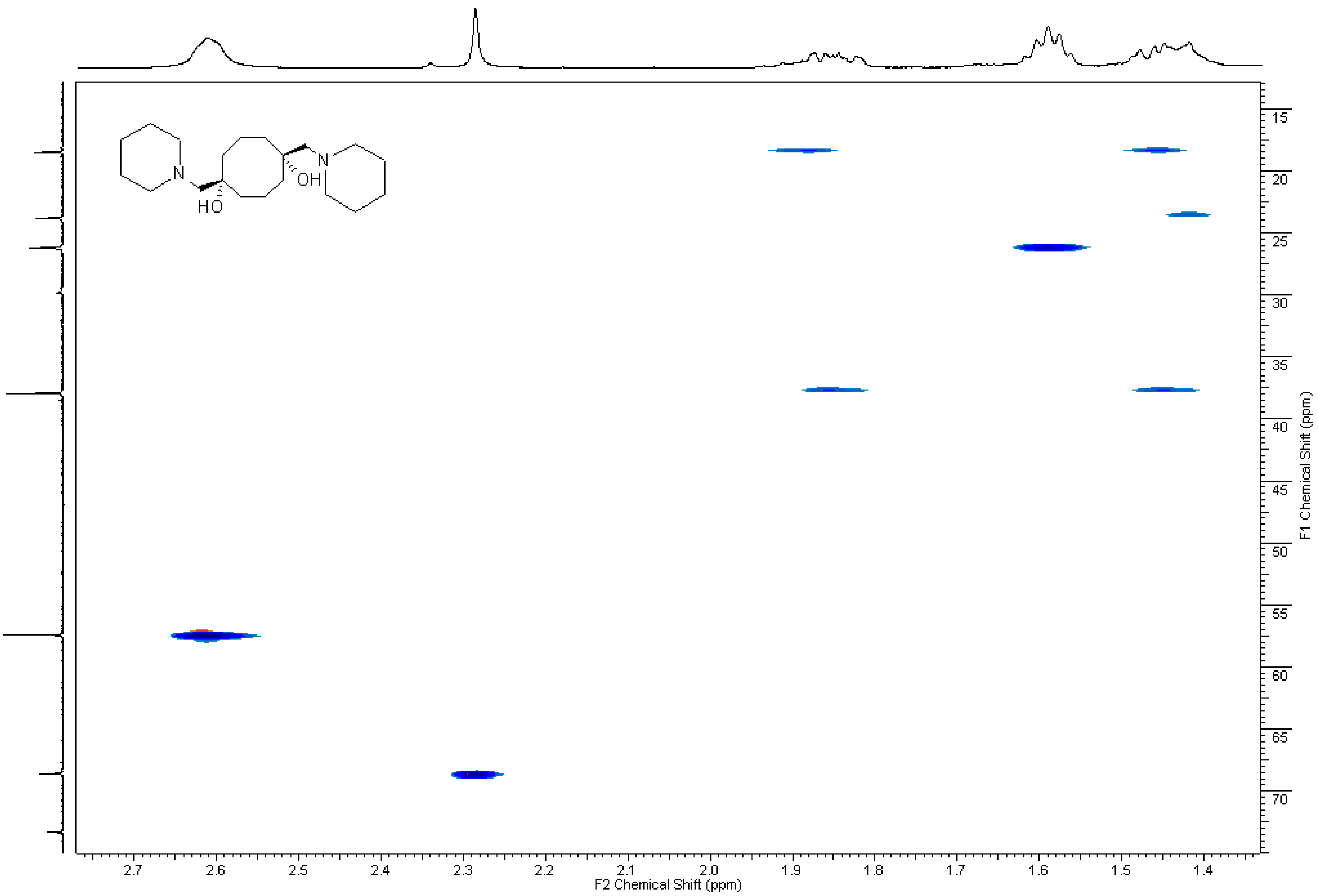

^{1}H NMR spectrum (400 MHz, $CDCl_3$) of compound **6e**

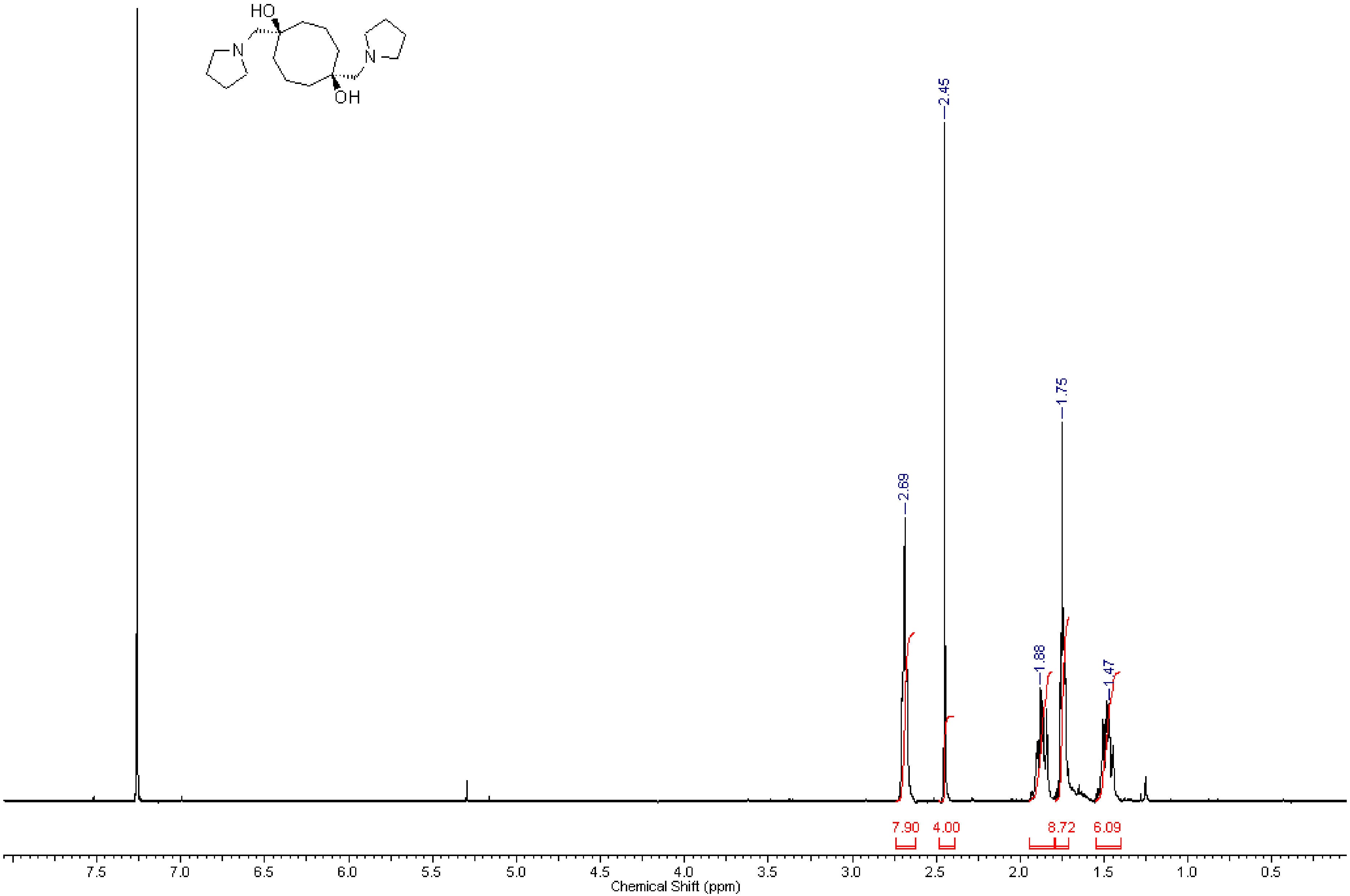

^{13}C NMR spectrum (101 MHz, $CDCl_3$) of compound **6e**

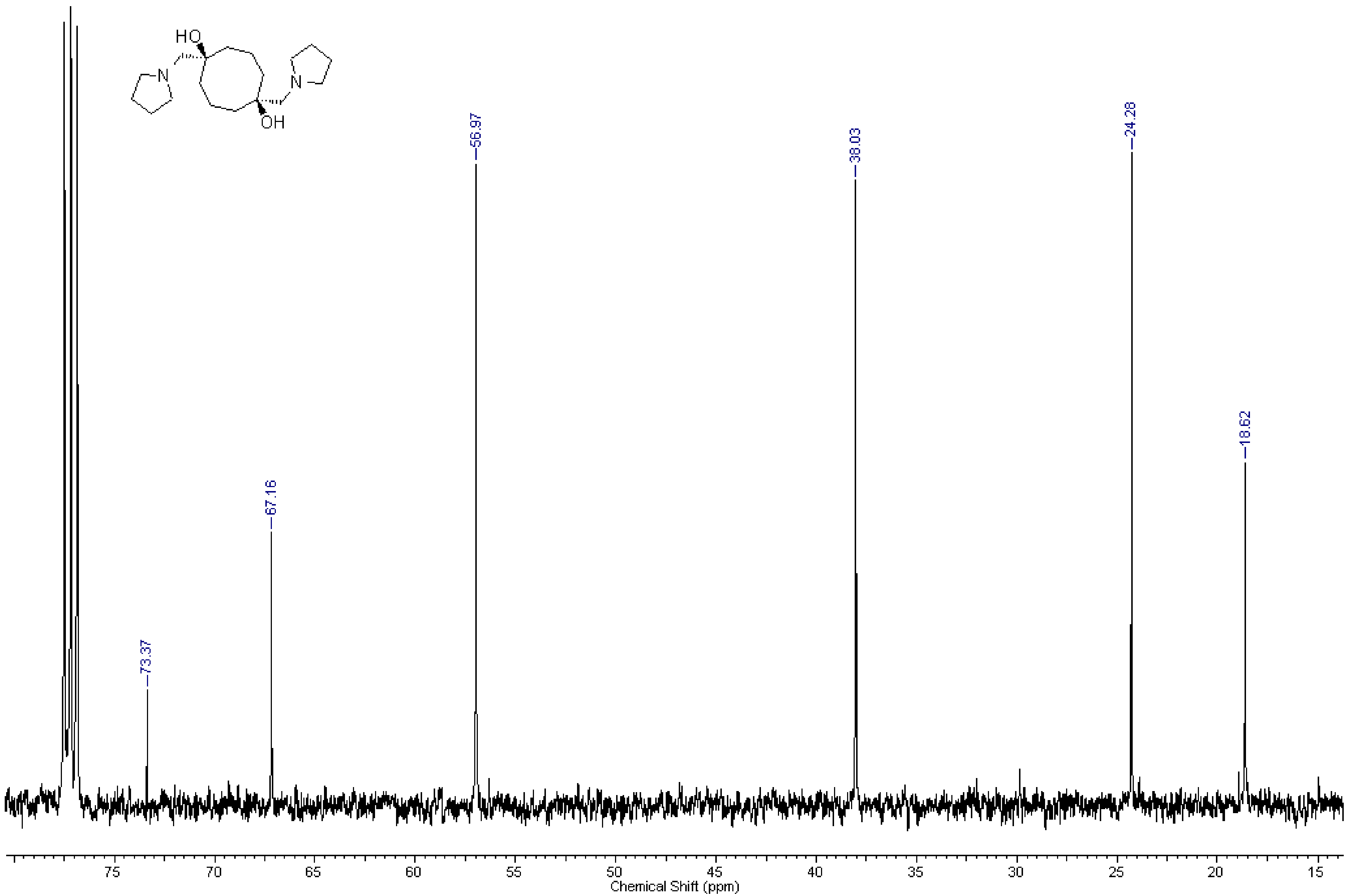

HSQC NMR spectrum of compound **6e**

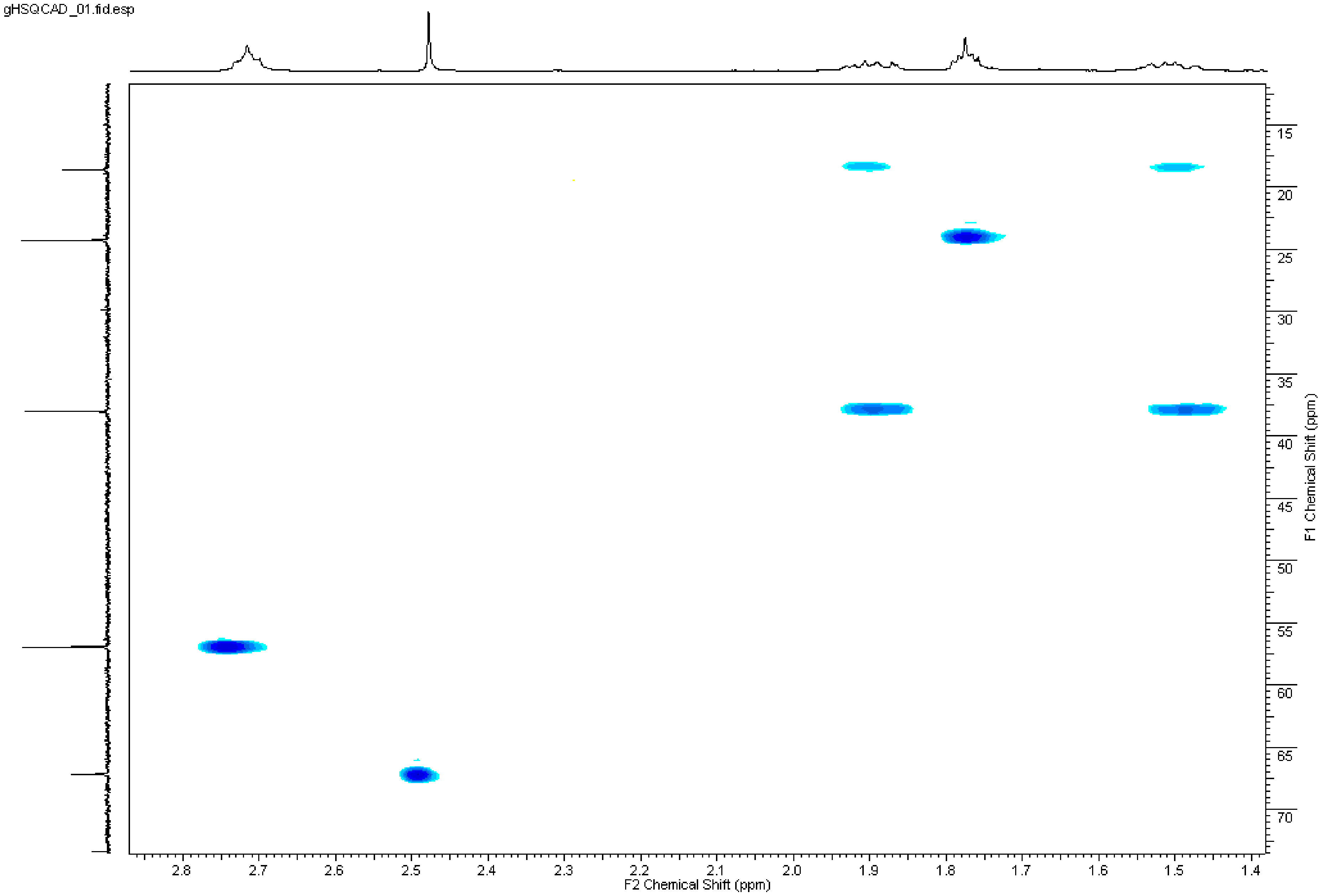

HMBC NMR spectrum of compound **6e**

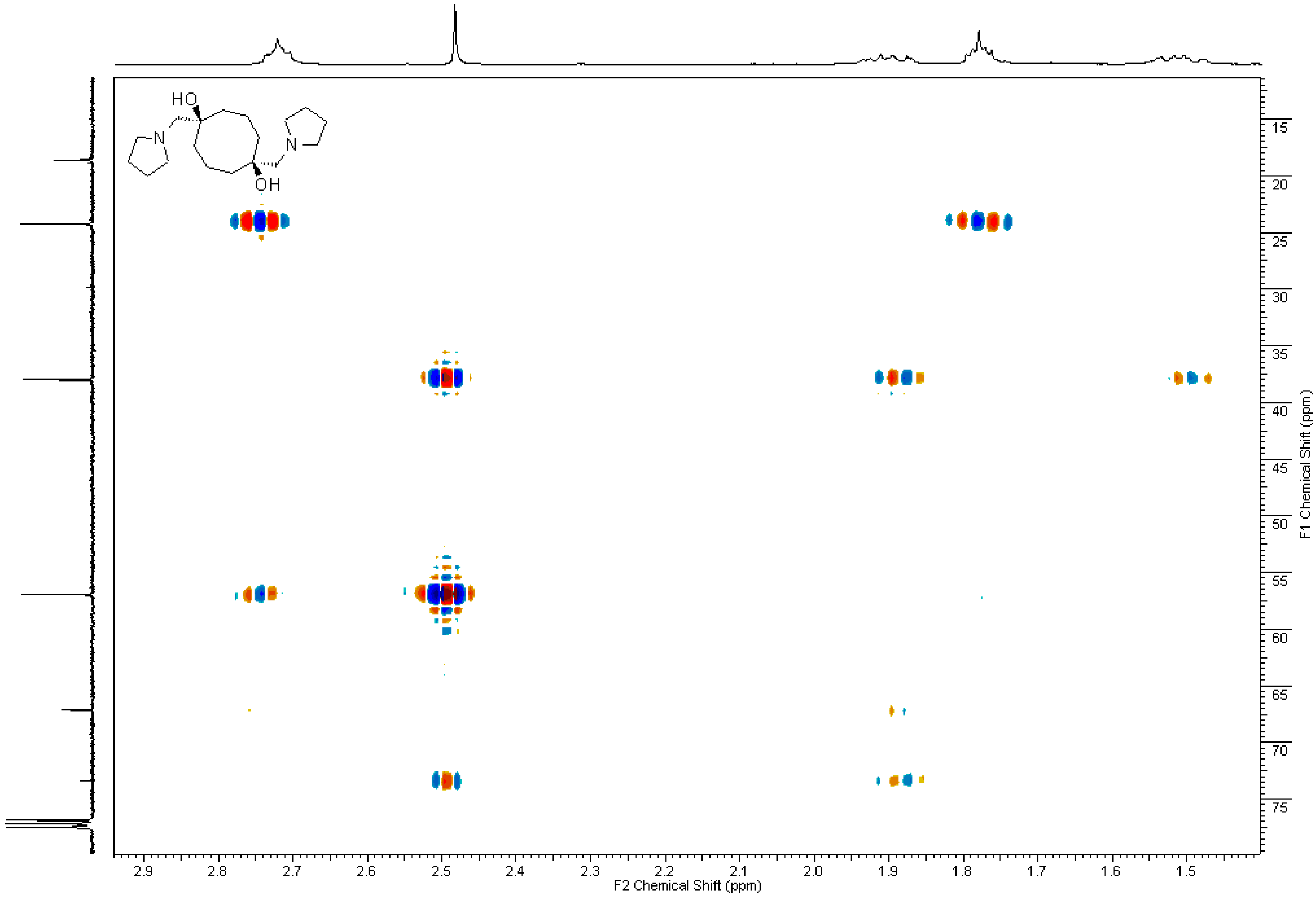

^{1}H NMR spectrum (400 MHz, CDCl$_3$) of compound **6f**

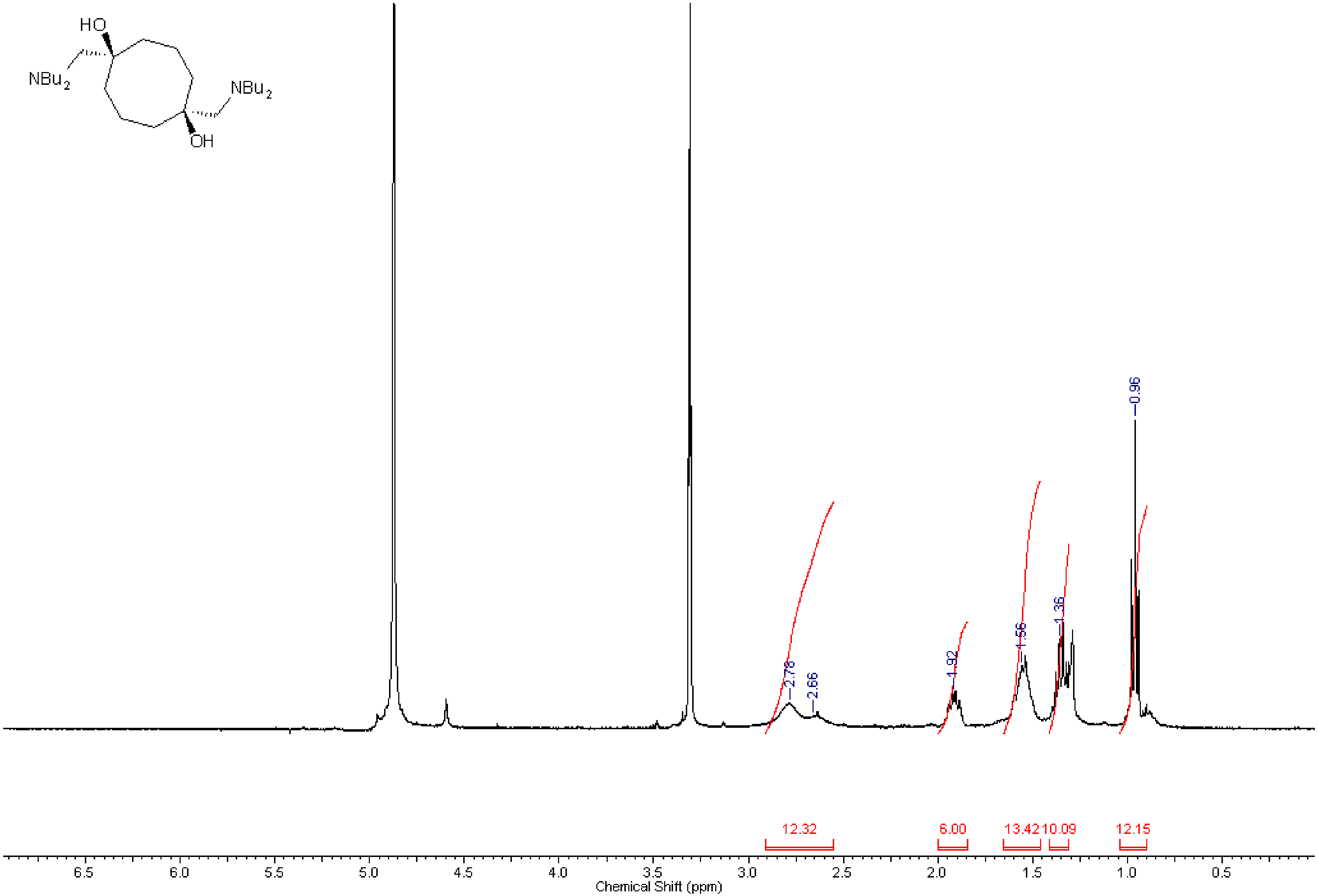

^{13}C NMR spectrum (101 MHz, $CDCl_3$) of compound **6f**

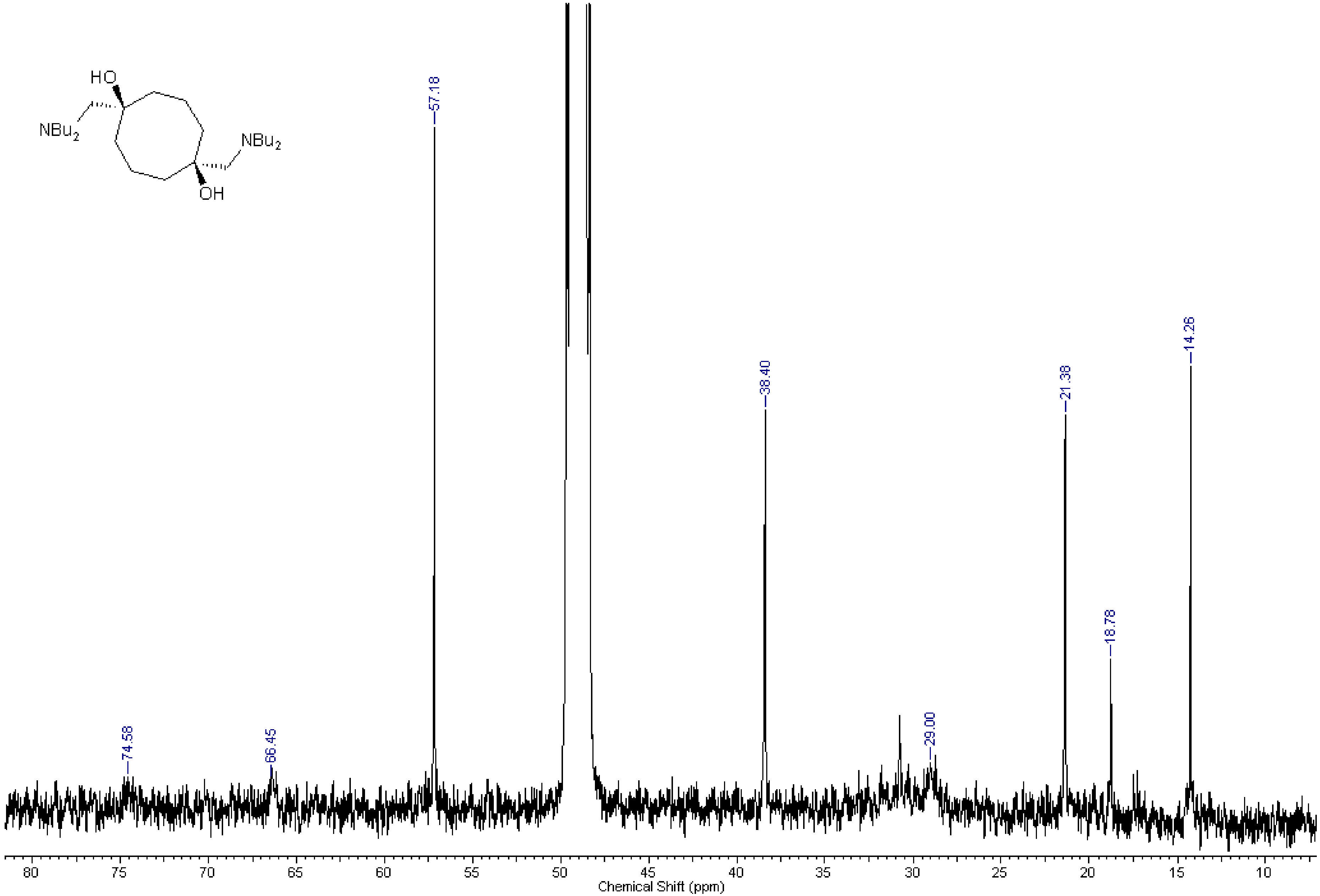

^{1}H NMR spectrum (400 MHz, $CDCl_3$) of compound **6l**

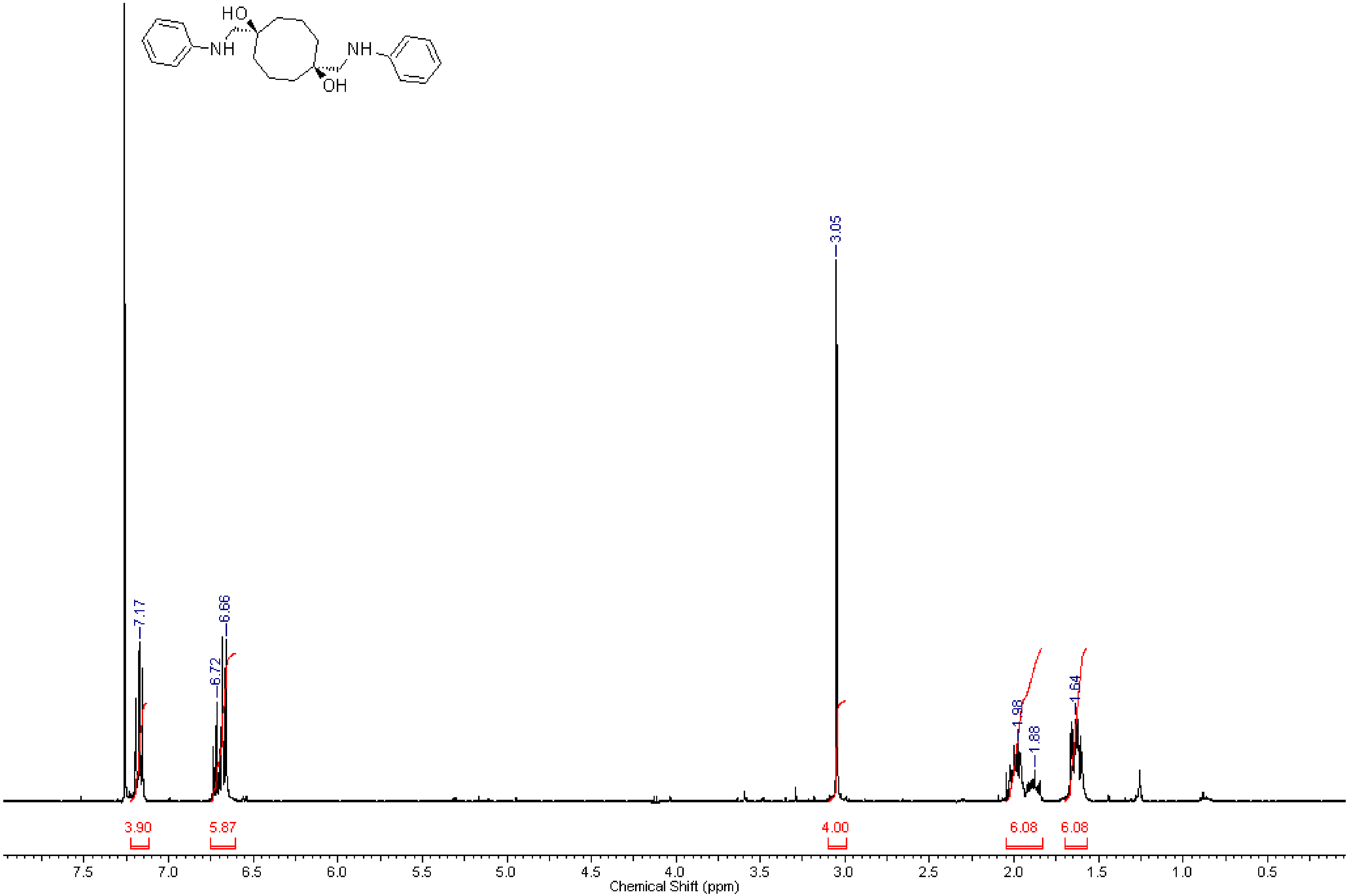

^{13}C NMR spectrum (101 MHz, $CDCl_3$) of compound **6l**

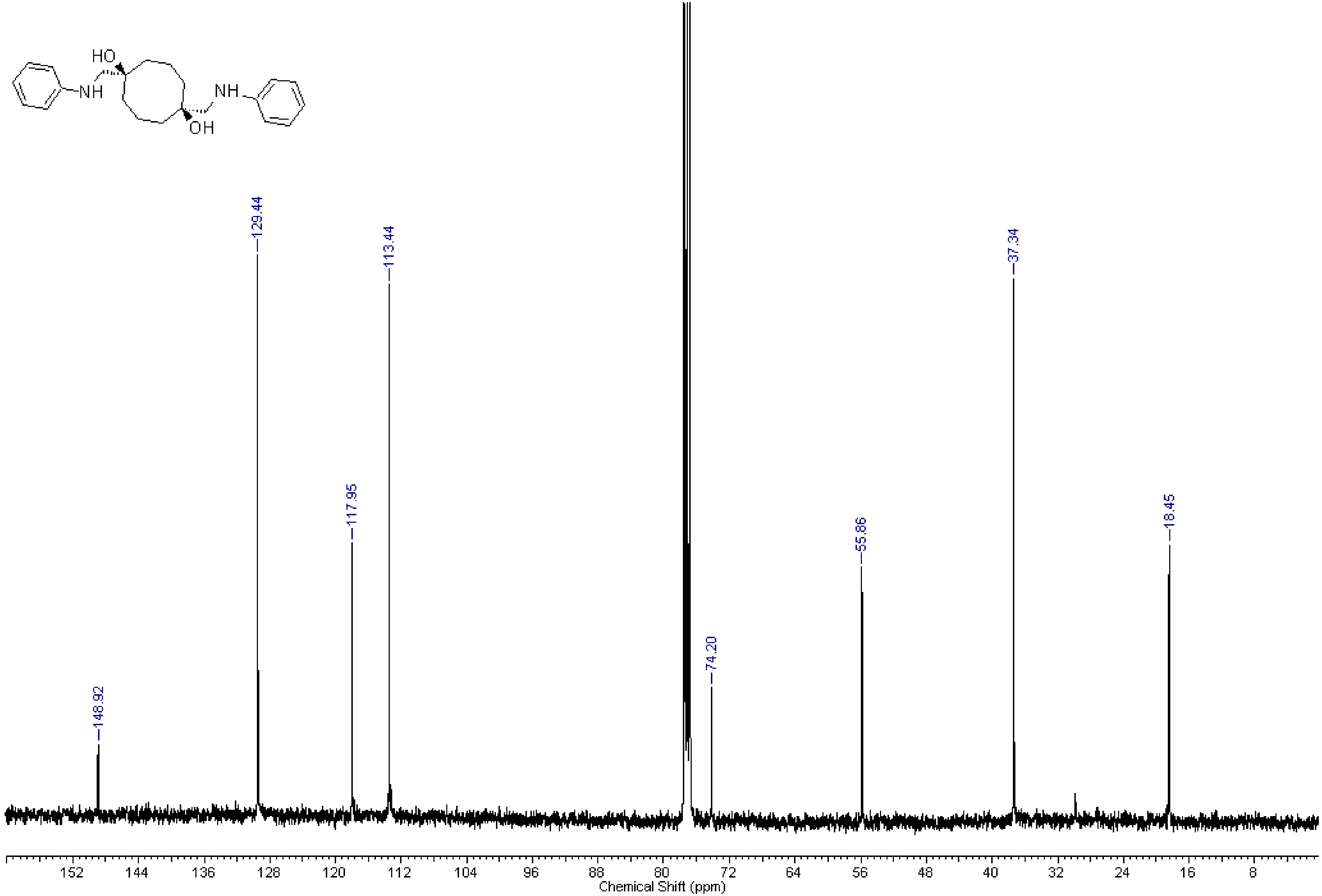

HSQC NMR spectrum of compound **6l**

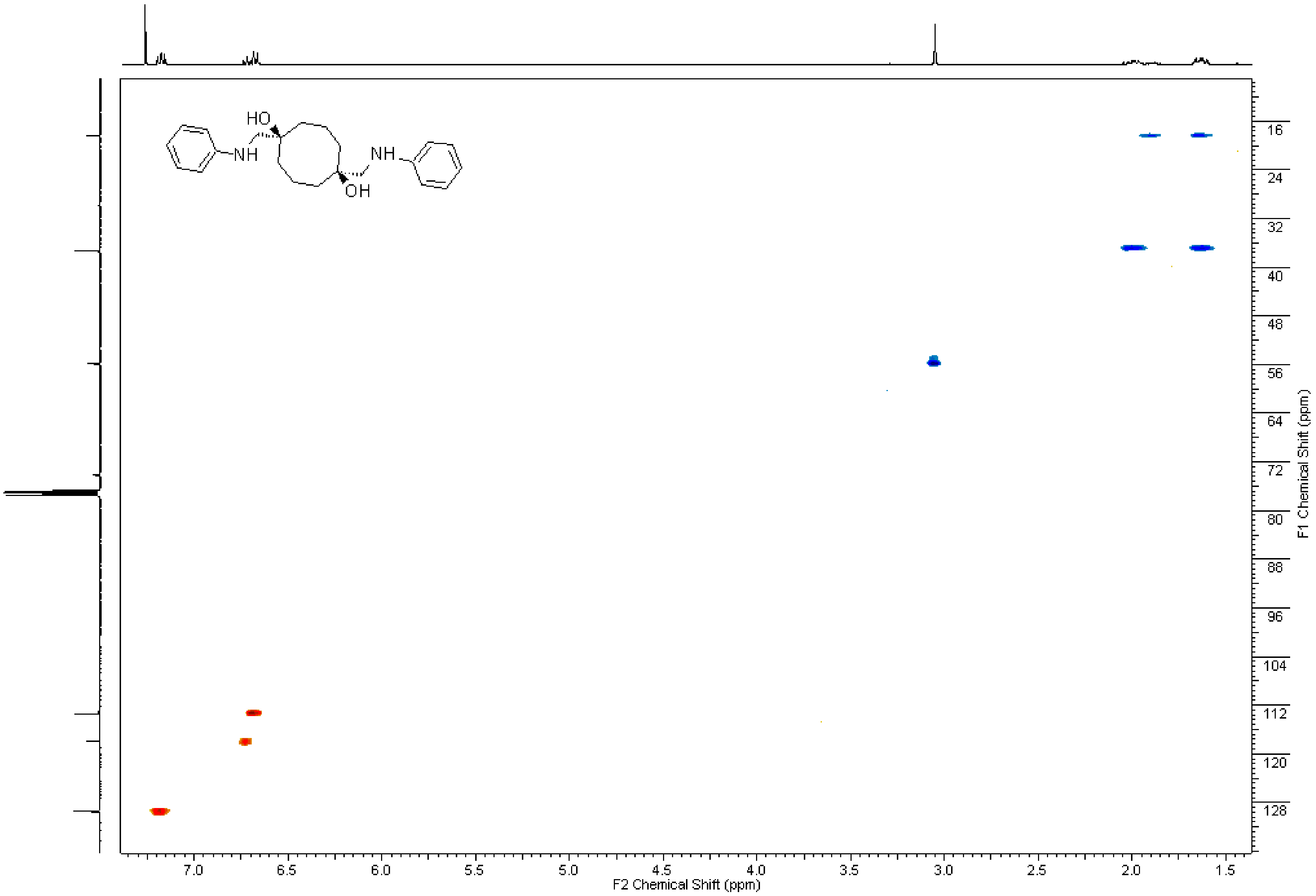

^{1}H NMR spectrum (400 MHz, $CDCl_3$) of compound **6o**

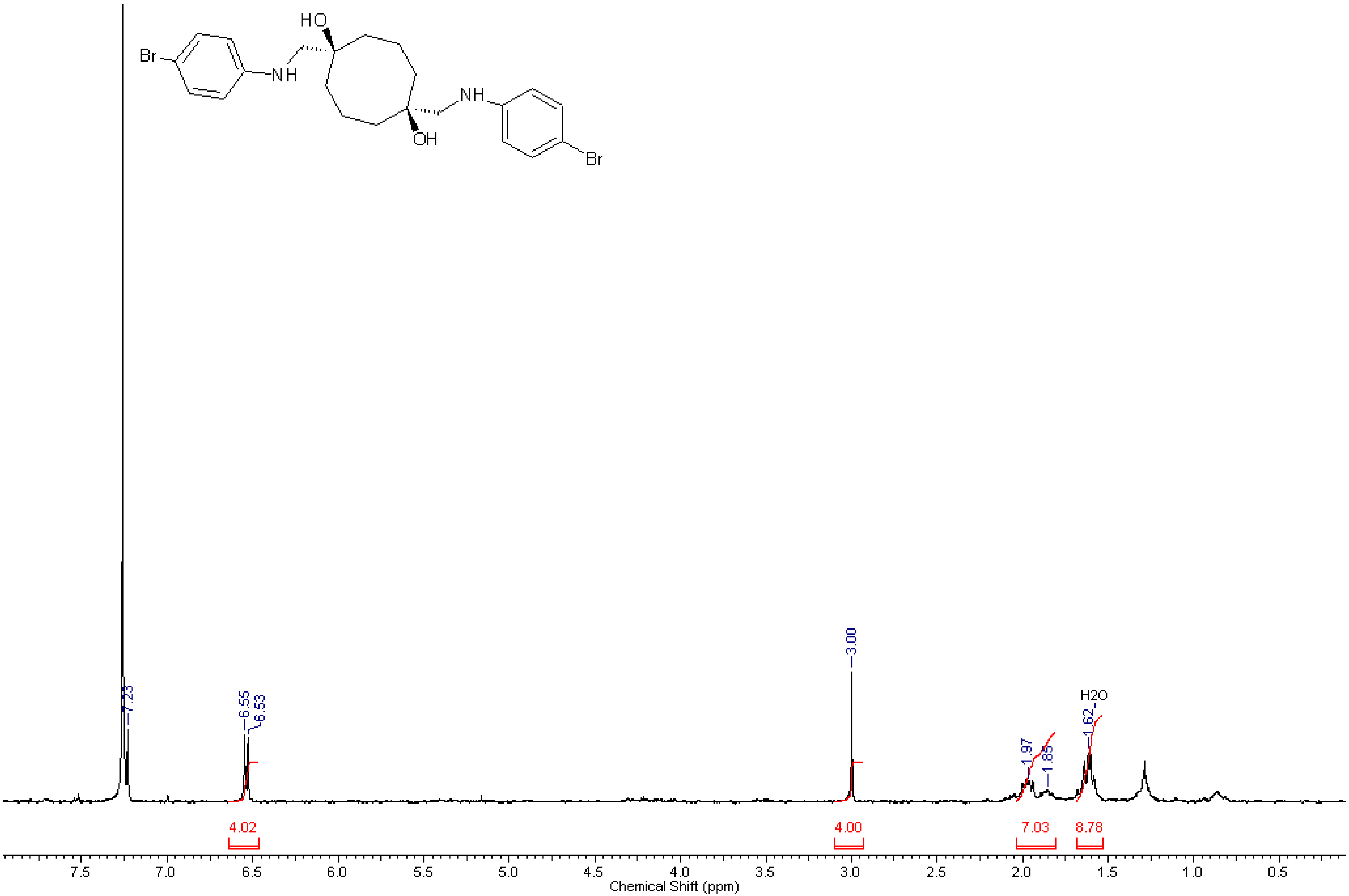

^{13}C NMR spectrum (101 MHz, $CDCl_3$) of compound **6o**

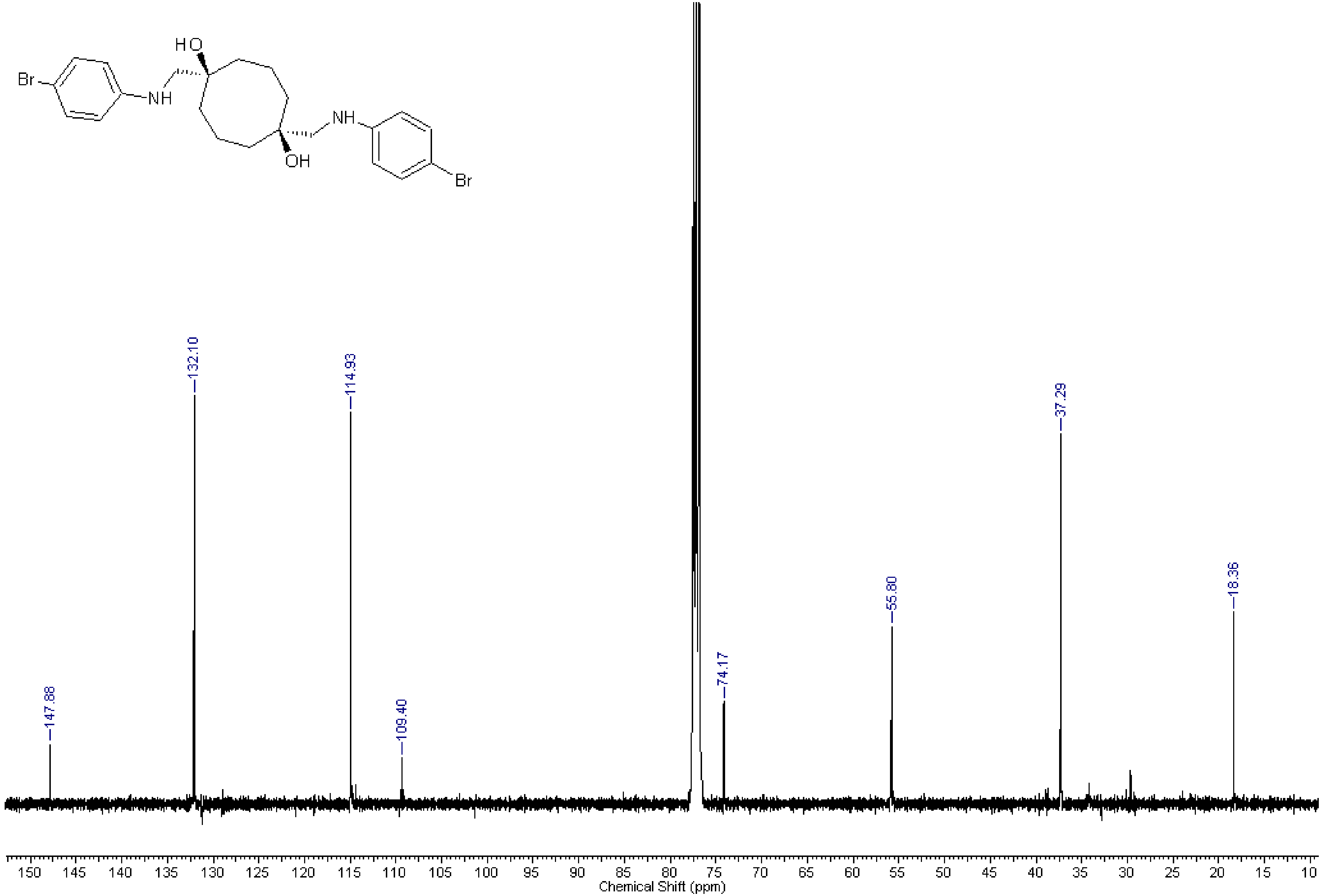

^{1}H NMR spectrum (400 MHz, $CDCl_3$) of compound **7j**

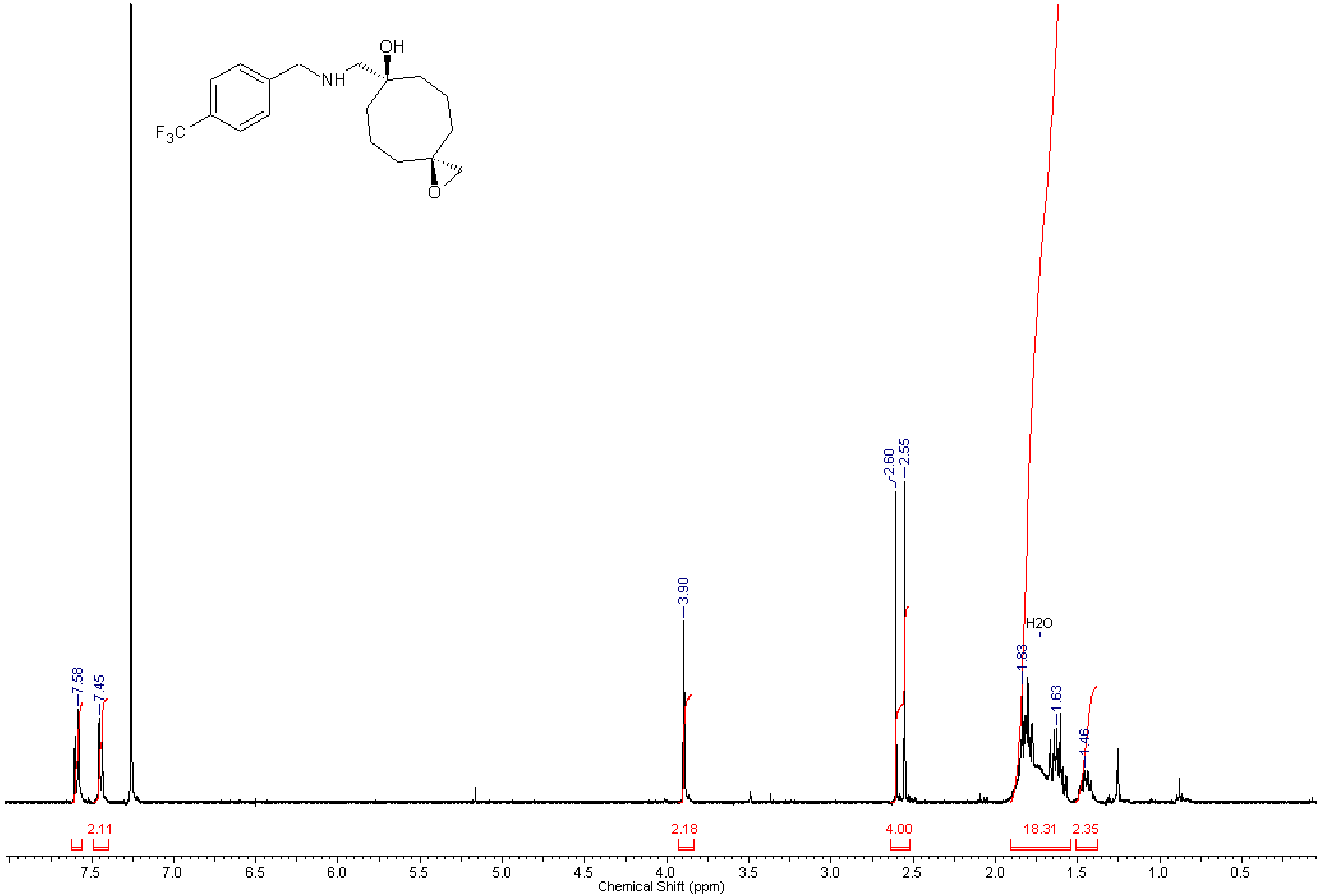

^{13}C NMR spectrum (101 MHz, $CDCl_3$) of compound **7j**

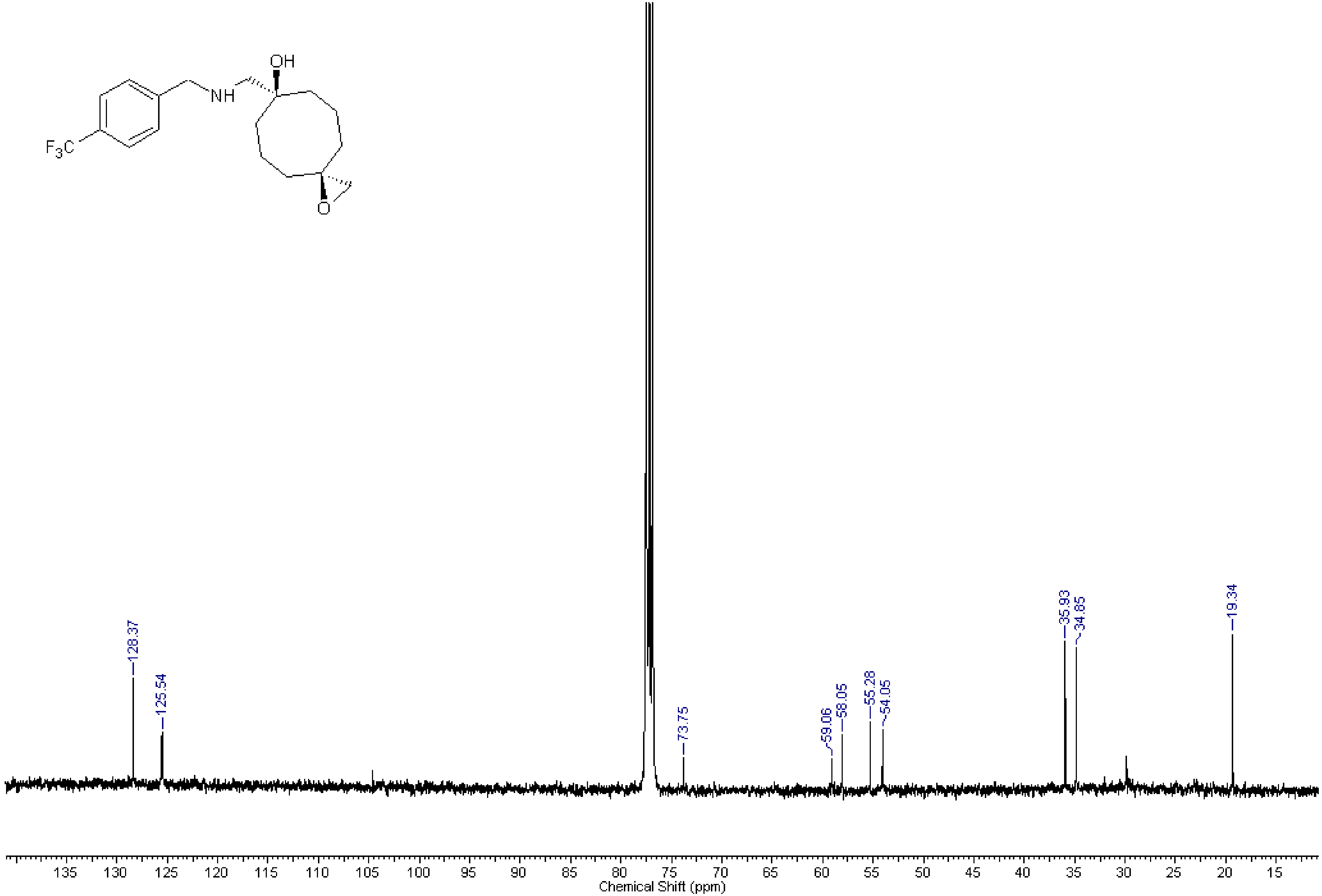

^{19}F NMR spectrum (376 MHz, $CDCl_3$) of compound **7j**

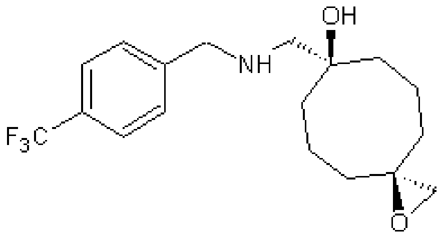

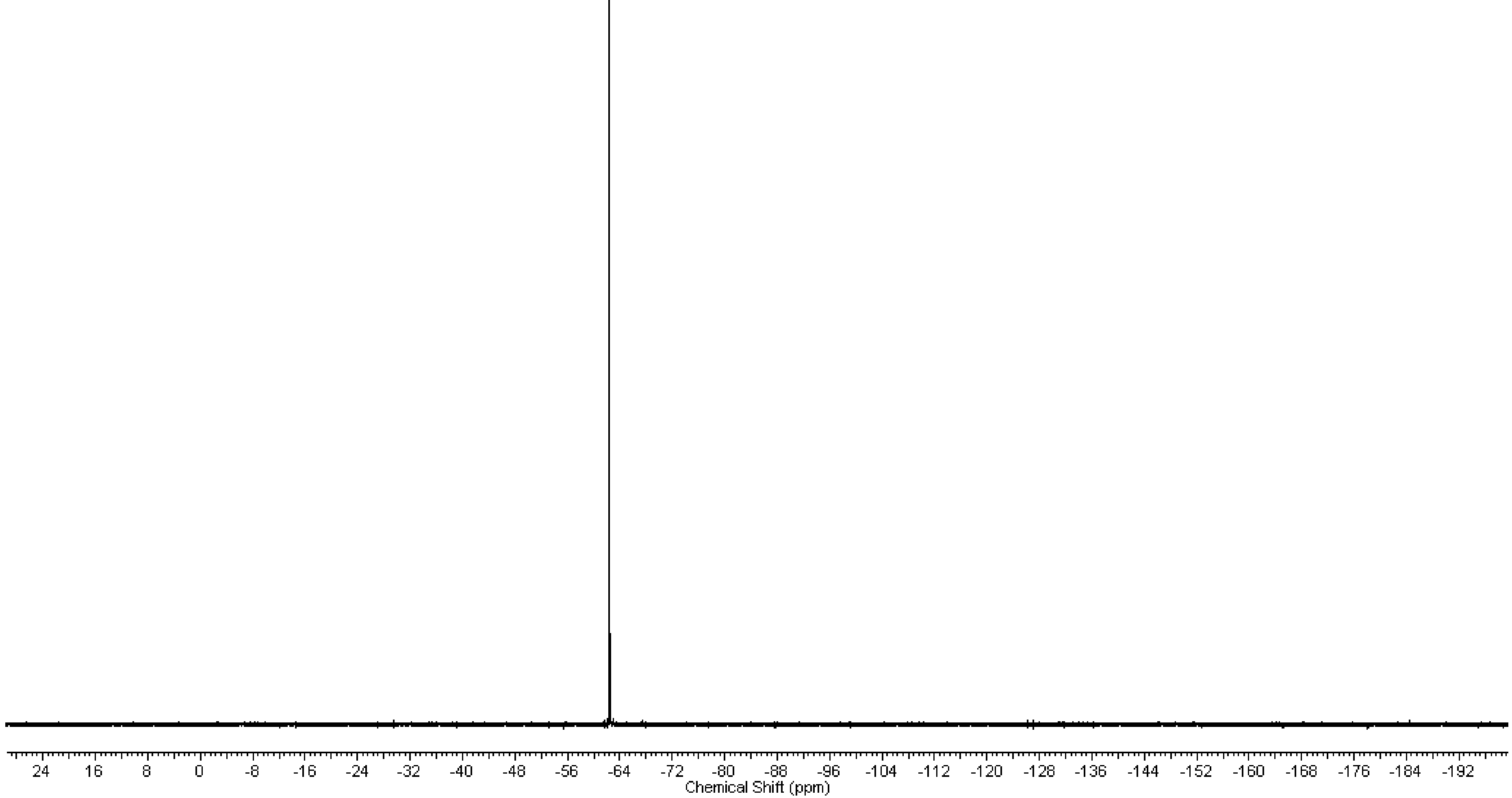

^{1}H NMR spectrum (400 MHz, $CDCl_3$) of compound **7o**

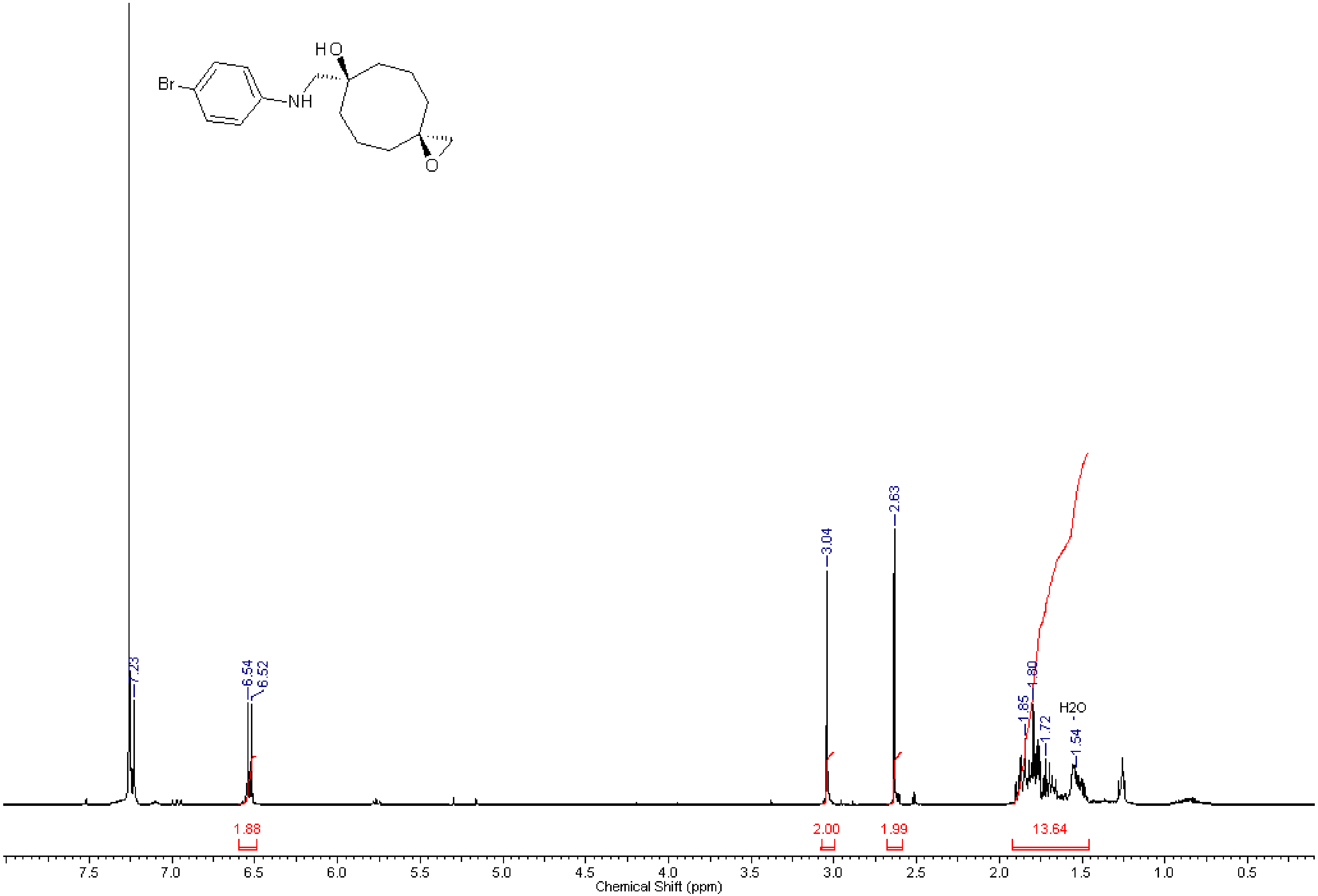

^{13}C NMR spectrum (101 MHz, $CDCl_3$) of compound **7o**

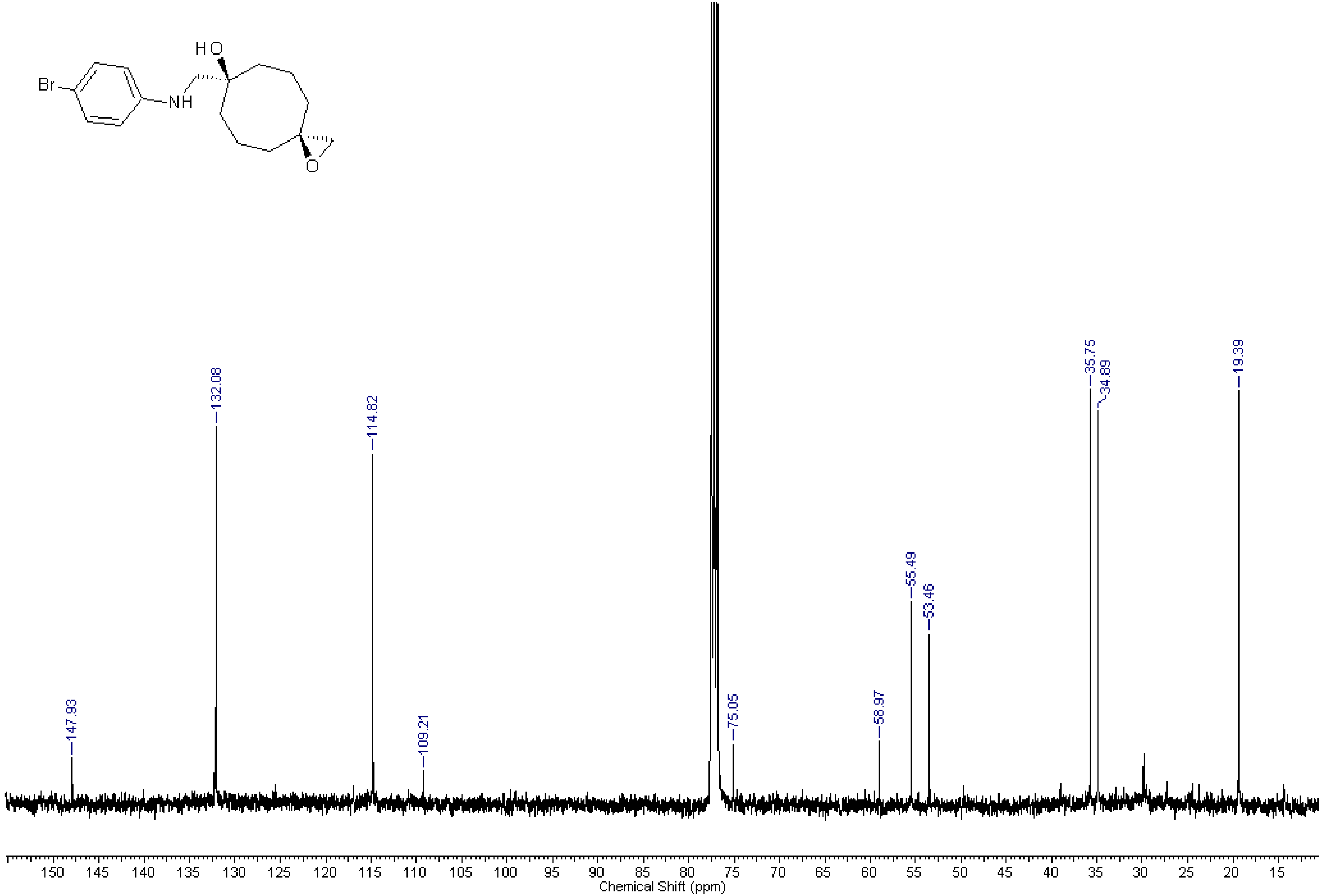

Supplement: Supplementary file 1 [file molecules-31-00252-s001.zip › molecules-4076183-supplementary.pdf]
